# Supplementary material for: A CRISPR-drug perturbational map for identifying compounds to combine with commonly used chemotherapeutics
Source: Nat Commun. 2023 Nov 13;14:7332. doi: 10.1038/s41467-023-43134-0 (PMC10643606; doi:10.1038/s41467-023-43134-0)
Supplement: Supplementary file 1 — Supplementary Information [file 41467_2023_43134_MOESM1_ESM.pdf]

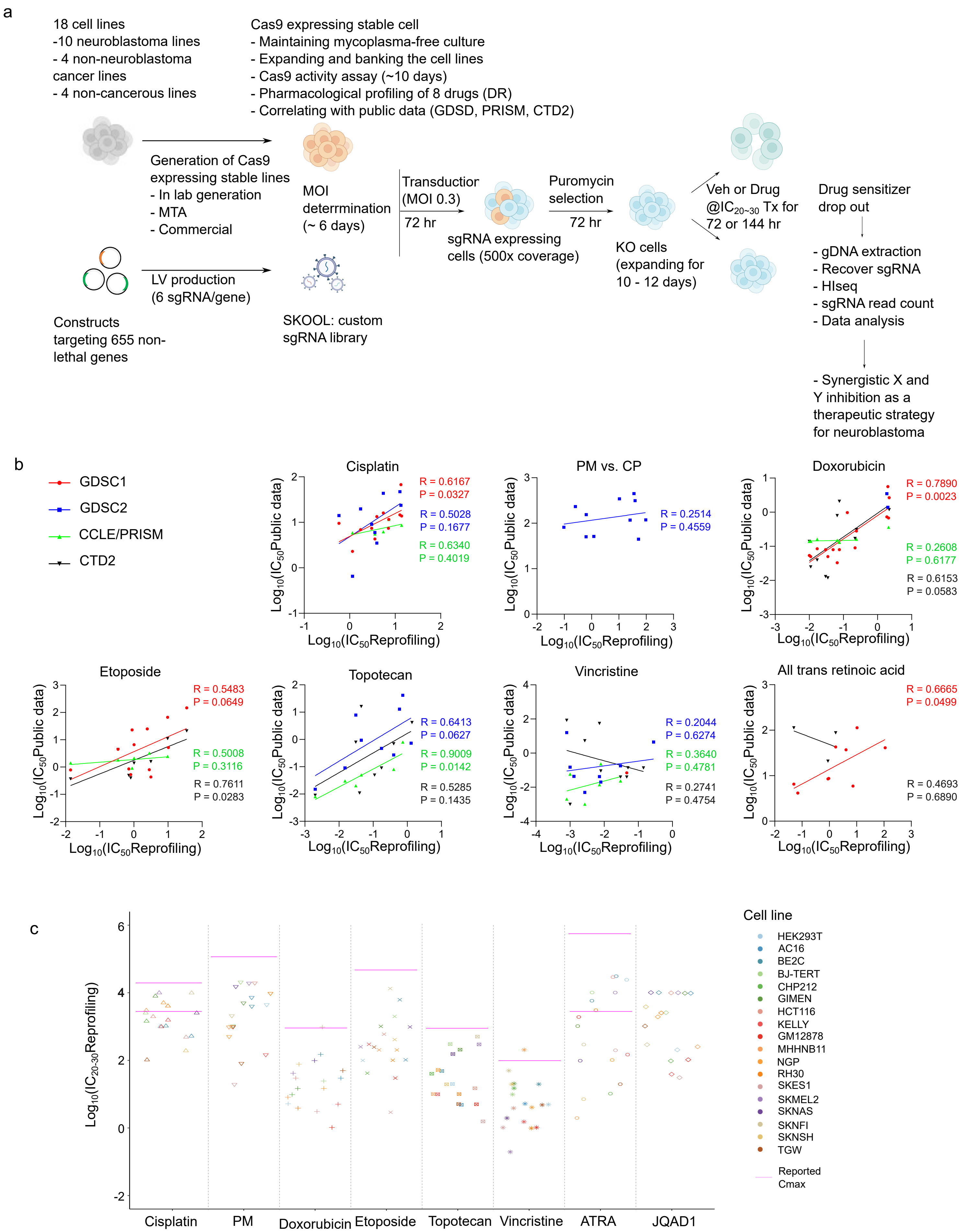
a
18 cell lines
-10 neuroblastoma lines
- 4 non-neuroblastoma cancer lines
- 4 non-cancerous lines
Cas9 expressing stable cell
- Maintaining mycoplasma-free culture
- Expanding and banking the cell lines
- Cas9 activity assay (~10 days)
- Pharmacological profiling of 8 drugs (DR)
- Correlating with public data (GDSD, PRISM, CTD2)
Generation of Cas9 expressing stable lines
- In lab generation
- MTA
- Commercial
MOI deterrmination (~ 6 days)
Transduction (MOI 0.3)
72 hr
sgRNA expressing cells (500x coverage)
Puromycin selection
72 hr
KO cells (expanding for 10 - 12 days)
Veh or Drug @IC20~30 Tx for 72 or 144 hr
Drug sensitizer drop out
- gDNA extraction
- Recover sgRNA
- HIseq
- sgRNA read count
- Data analysis
- Synergistic X and Y inhibition as a therapeutic strategy for neuroblastoma
LV production (6 sgRNA/gene)
Constructs targeting 655 non-lethal genes
SKOOL: custom sgRNA library
b
GDSC1
GDSC2
CCLE/PRISM
CTD2
Cisplatin
R = 0.6167
P = 0.0327
R = 0.5028
P = 0.1677
R = 0.6340
P = 0.4019
PM vs. CP
R = 0.2514
P = 0.4559
Doxorubicin
R = 0.7890
P = 0.0023
R = 0.2608
P = 0.6177
R = 0.6153
P = 0.0583
Etoposide
R = 0.5483
P = 0.0649
R = 0.5008
P = 0.3116
R = 0.7611
P = 0.0283
Topotecan
R = 0.6413
P = 0.0627
R = 0.9009
P = 0.0142
R = 0.5285
P = 0.1435
Vincristine
R = 0.2044
P = 0.6274
R = 0.3640
P = 0.4781
R = 0.2741
P = 0.4754
All trans retinoic acid
R = 0.6665
P = 0.0499
R = 0.4693
P = 0.6890
Log10(IC50Public data)
Log10(IC50Reprofiling)
c
Log10(IC20-30Reprofiling)
Cisplatin
PM
Doxorubicin
Etoposide
Topotecan
Vincristine
ATRA
JQAD1
Cell line
HEK293T
AC16
BE2C
BJ-TERT
CHP212
GIMEN
HCT116
KELLY
GM12878
MHHNB11
NGP
RH30
SKES1
SKMEL2
SKNAS
SKNFI
SKNSH
TGW
Reported Cmax

Supplementary Figure 1. Drug screening details.

(a) Detailed schematic of CRISPR-drug screens.
(b) Scatterplots showing the correlation of IC50 values obtained for each of our 8 drugs plotted against IC50 values reported in the public GDSC/CCLE/CTD[2] screening datasets from the Sanger/ Broad Institutes. P value and r were calculated by Pearson correlation.
(c) A table with all IC20-30 used across all screens (the pink lines show known Cmax values, note in resistant cells we generally capped our screening concentrations approximately at these Cmax values)

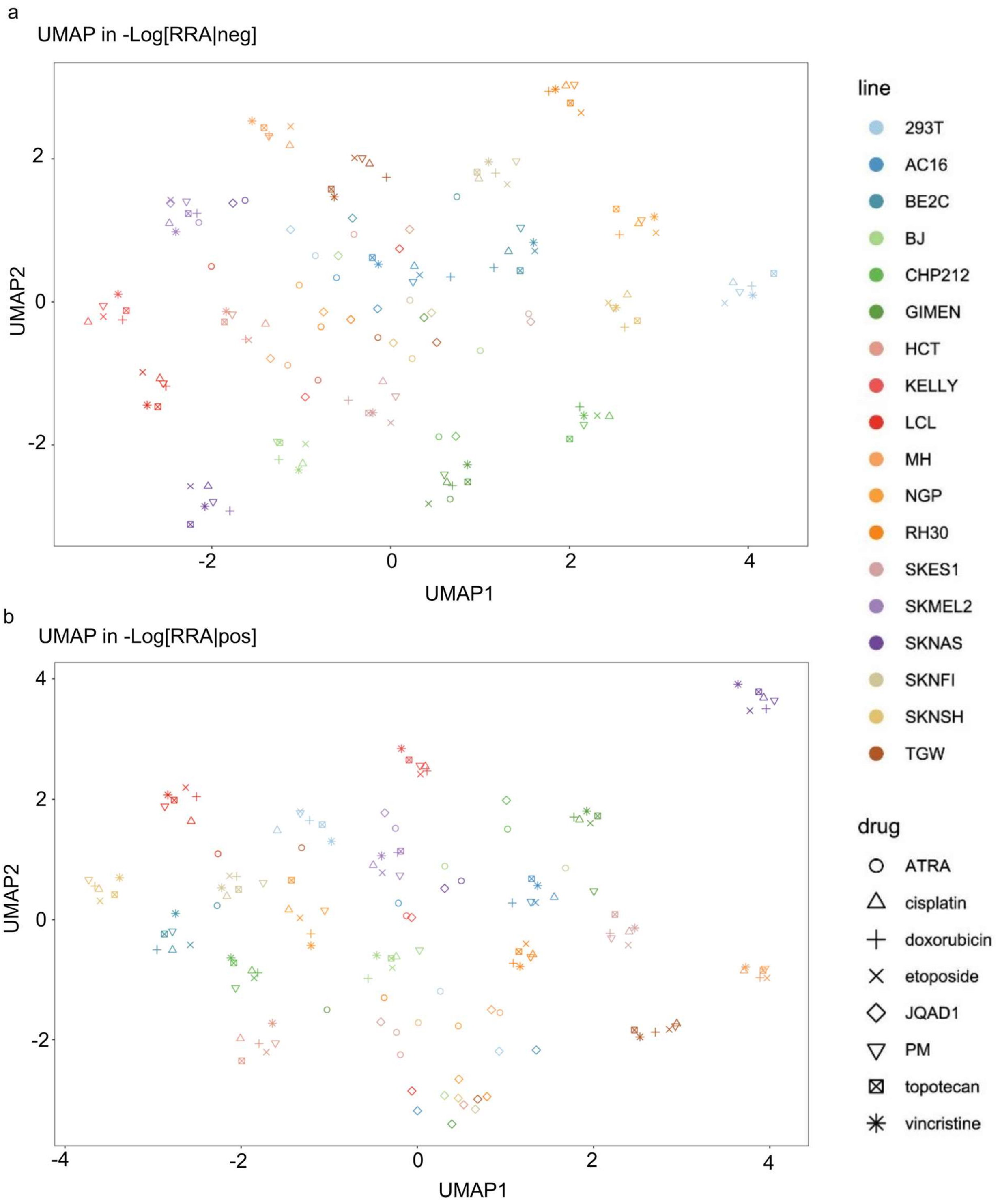
a
UMAP in -Log[RRA|neg]
UMAP1
UMAP2
b
UMAP in -Log[RRA|pos]
UMAP1
UMAP2
line
293T
AC16
BE2C
BJ
CHP212
GIMEN
HCT
KELLY
LCL
MH
NGP
RH30
SKES1
SKMEL2
SKNAS
SKNFI
SKNSH
TGW
drug
ATRA
cisplatin
doxorubicin
etoposide
JQAD1
PM
topotecan
vincristine

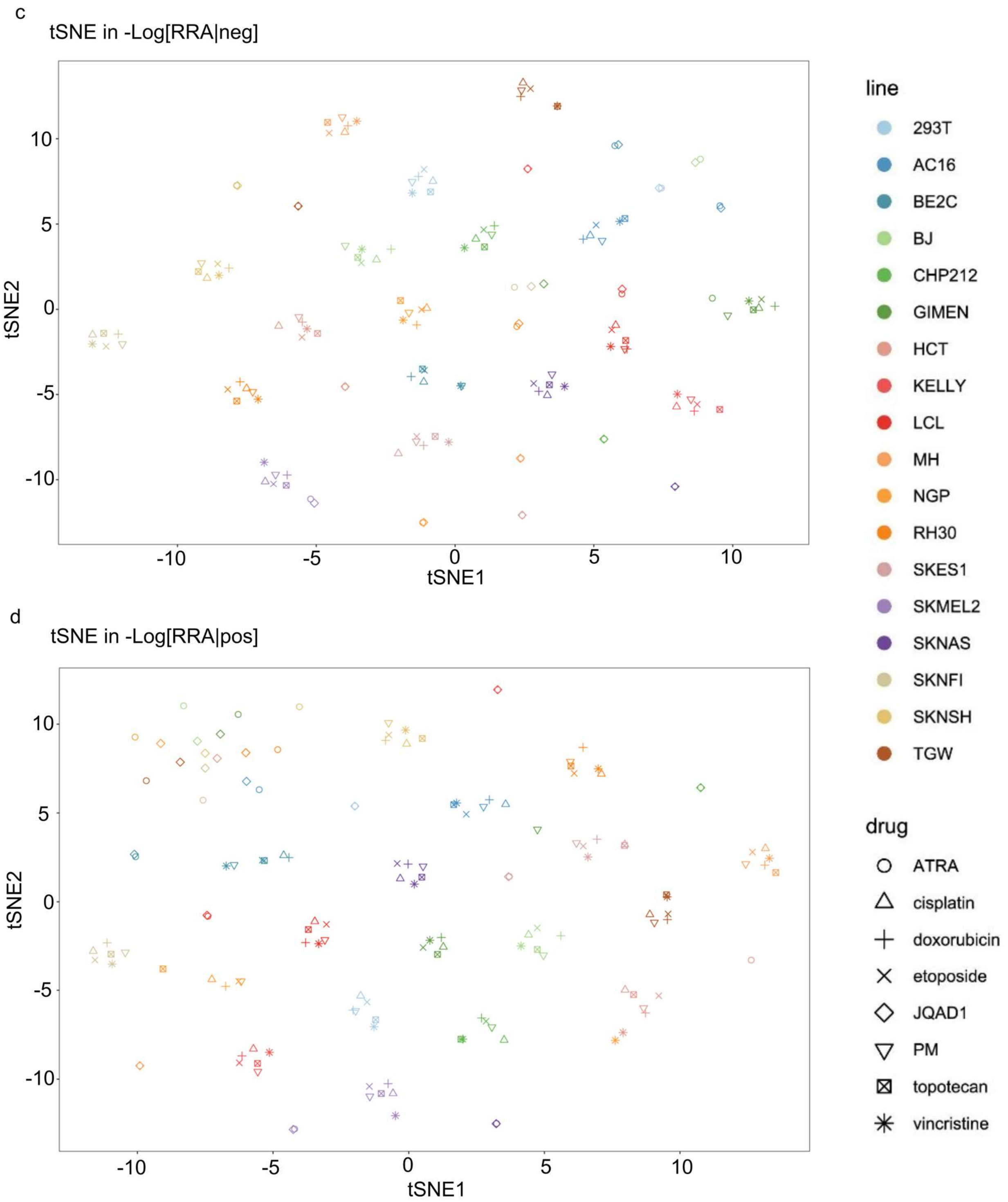
c
tSNE in -Log[RRA|neg]
tSNE2
tSNE1
d
tSNE in -Log[RRA|pos]
tSNE2
tSNE1
line
293T
AC16
BE2C
BJ
CHP212
GIMEN
HCT
KELLY
LCL
MH
NGP
RH30
SKES1
SKMEL2
SKNAS
SKNFI
SKNSH
TGW
drug
ATRA
cisplatin
doxorubicin
etoposide
JQAD1
PM
topotecan
vincristine

e

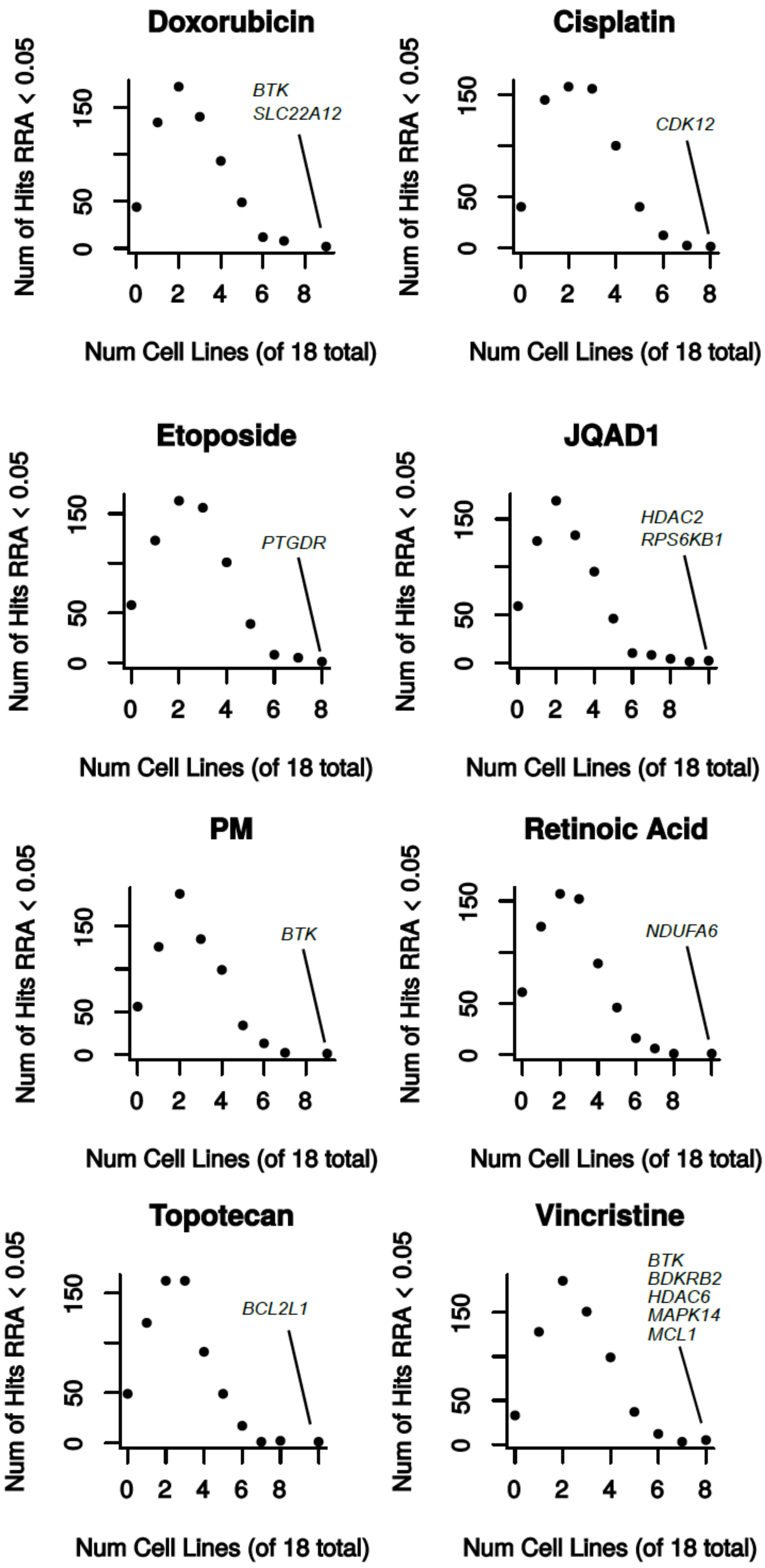
Doxorubicin
BTK
SLC22A12
Cisplatin
CDK12
Etoposide
PTGDR
JQAD1
HDAC2
RPS6KB1
PM
BTK
Retinoic Acid
NDUFA6
Topotecan
BCL2L1
Vincristine
BTK
BDKRB2
HDAC6
MAPK14
MCL1
Num of Hits RRA < 0.05
Num Cell Lines (of 18 total)

f

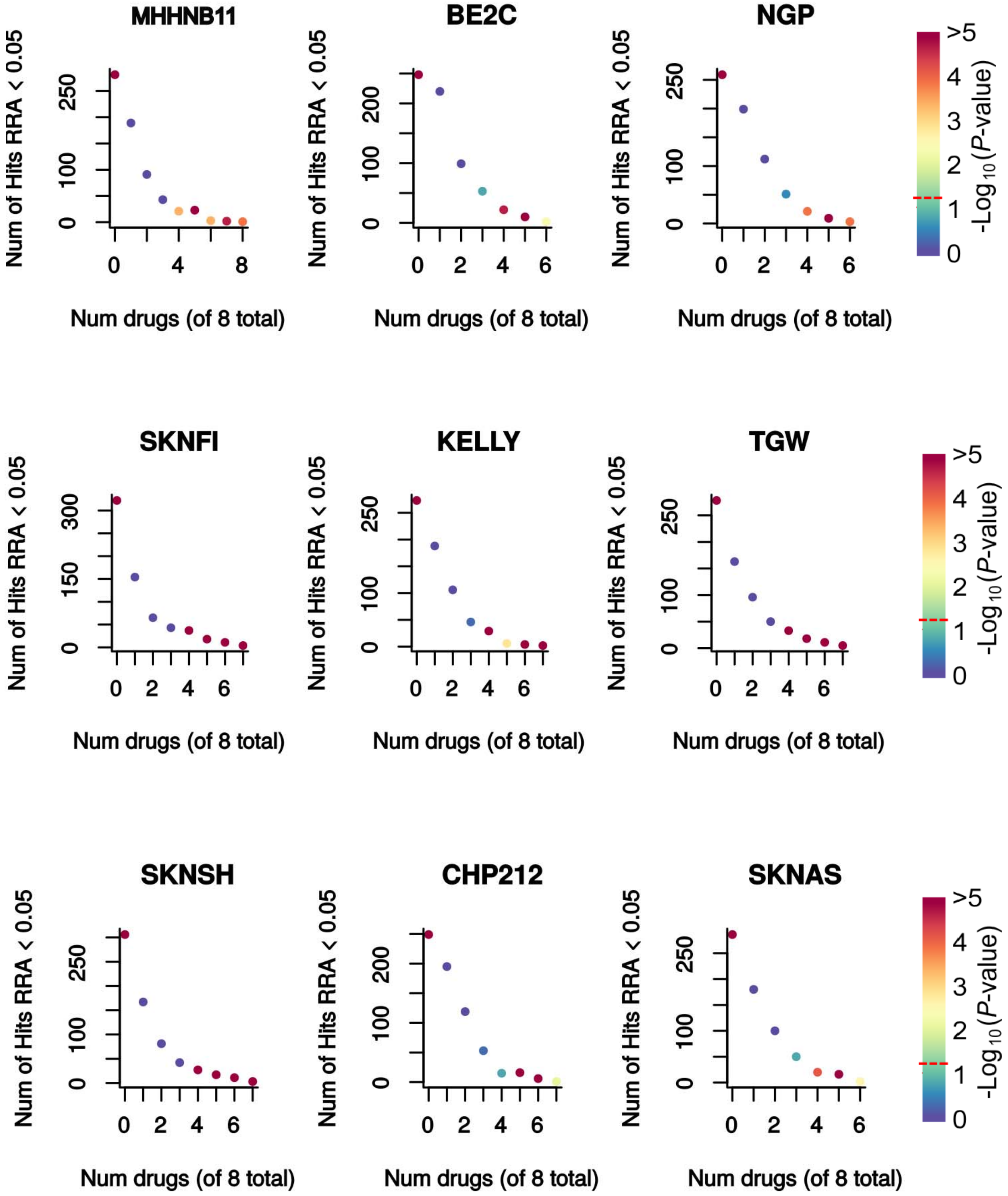
MHHNB11
BE2C
NGP
SKNFI
KELLY
TGW
SKNSH
CHP212
SKNAS
Num of Hits RRA < 0.05
Num drugs (of 8 total)
-Log10(P-value)
>5

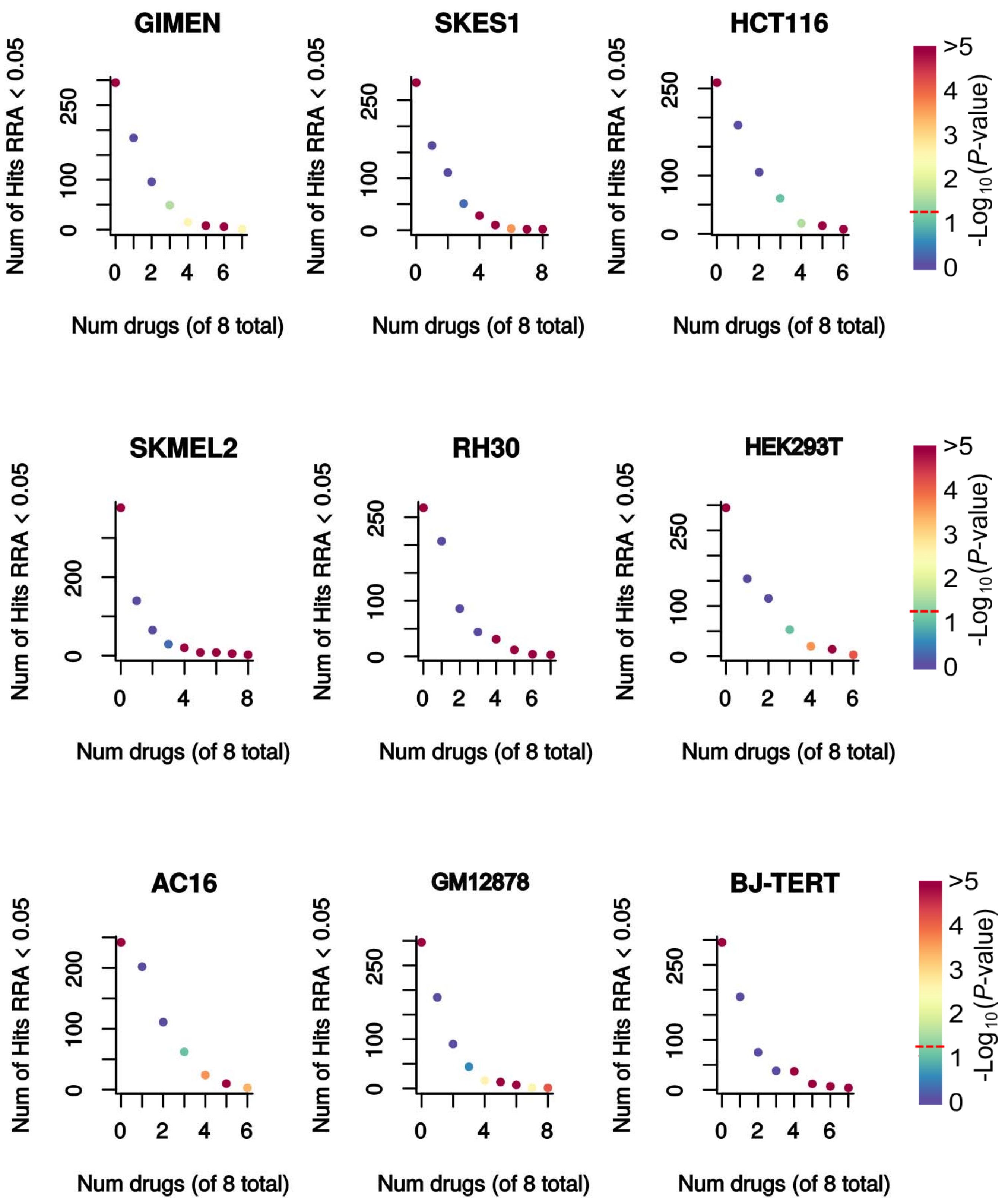

GIMEN
SKES1
HCT116
SKMEL2
RH30
HEK293T
AC16
GM12878
BJ-TERT
Num of Hits RRA < 0.05
Num drugs (of 8 total)
-Log10(P-value)
>5
4
3
2
1
0

Supplementary Figure 2: Details of systematic behaviors in screen.

(a, b) Extra UMAP plots of all RRA scores for sensitization (a) and resistance (b) in screen.
(c, d) tSNE plots of all RRA scores for sensitization (c) and resistance (d) in screen.
(e) Scatterplot showing the number of cell lines (x axis) that are sensitized to some gene knockout (RRA < 0.05; y axis) for 8 drugs screened.
(f) Scatterplot showing the number of drugs (x axis) that are sensitized to some gene knockout (RRA < 0.05; y axis) for all 18 cell lines screened. P-values (colors) were calculated by permutation.

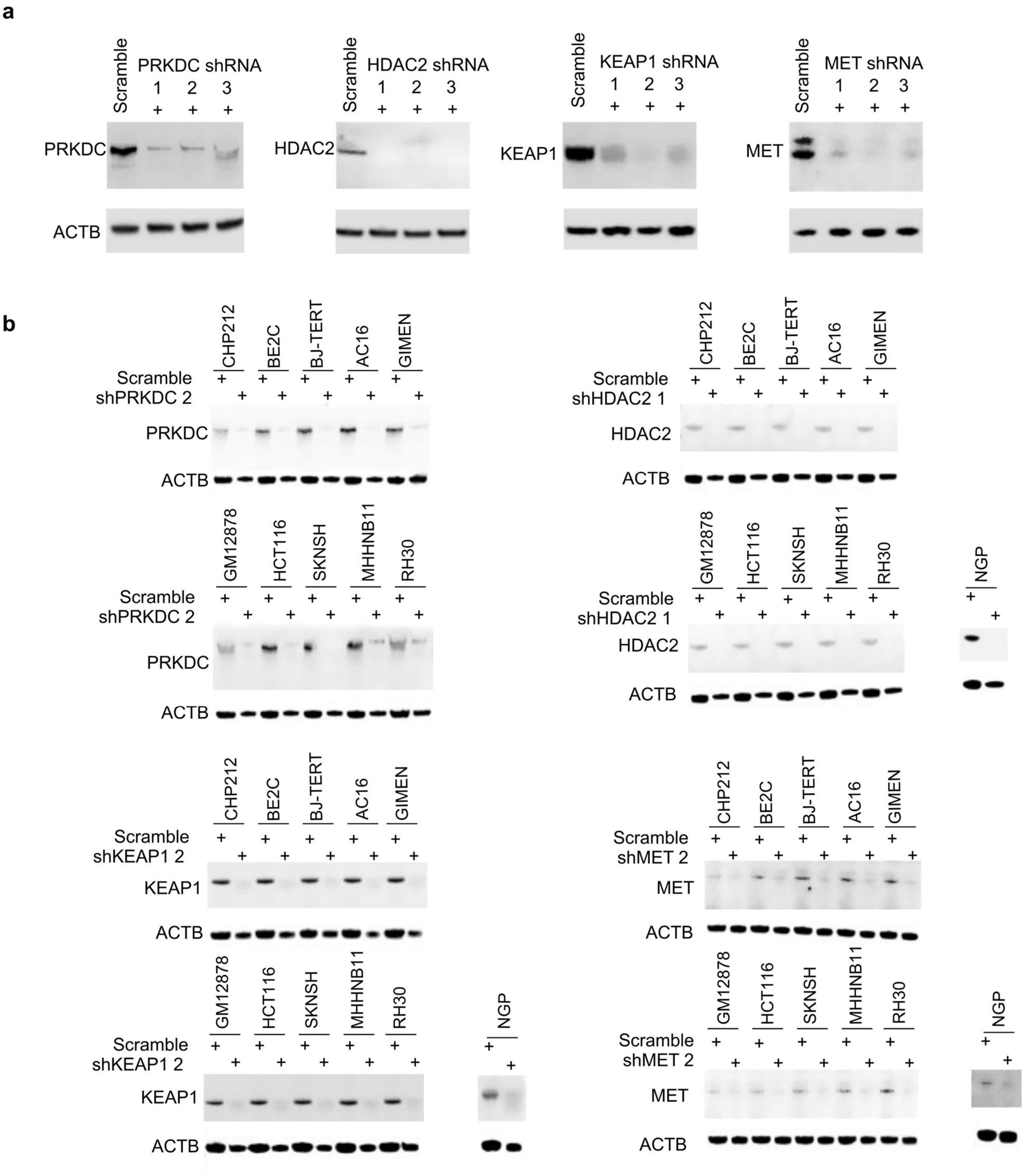
a
Scramble
PRKDC shRNA
1 2 3
PRKDC
ACTB
HDAC2 shRNA
HDAC2
KEAP1 shRNA
KEAP1
MET shRNA
MET
b
CHP212
BE2C
BJ-TERT
AC16
GIMEN
Scramble
shPRKDC 2
shHDAC2 1
GM12878
HCT116
SKNSH
MHHNB11
RH30
NGP
shKEAP1 2
shMET 2

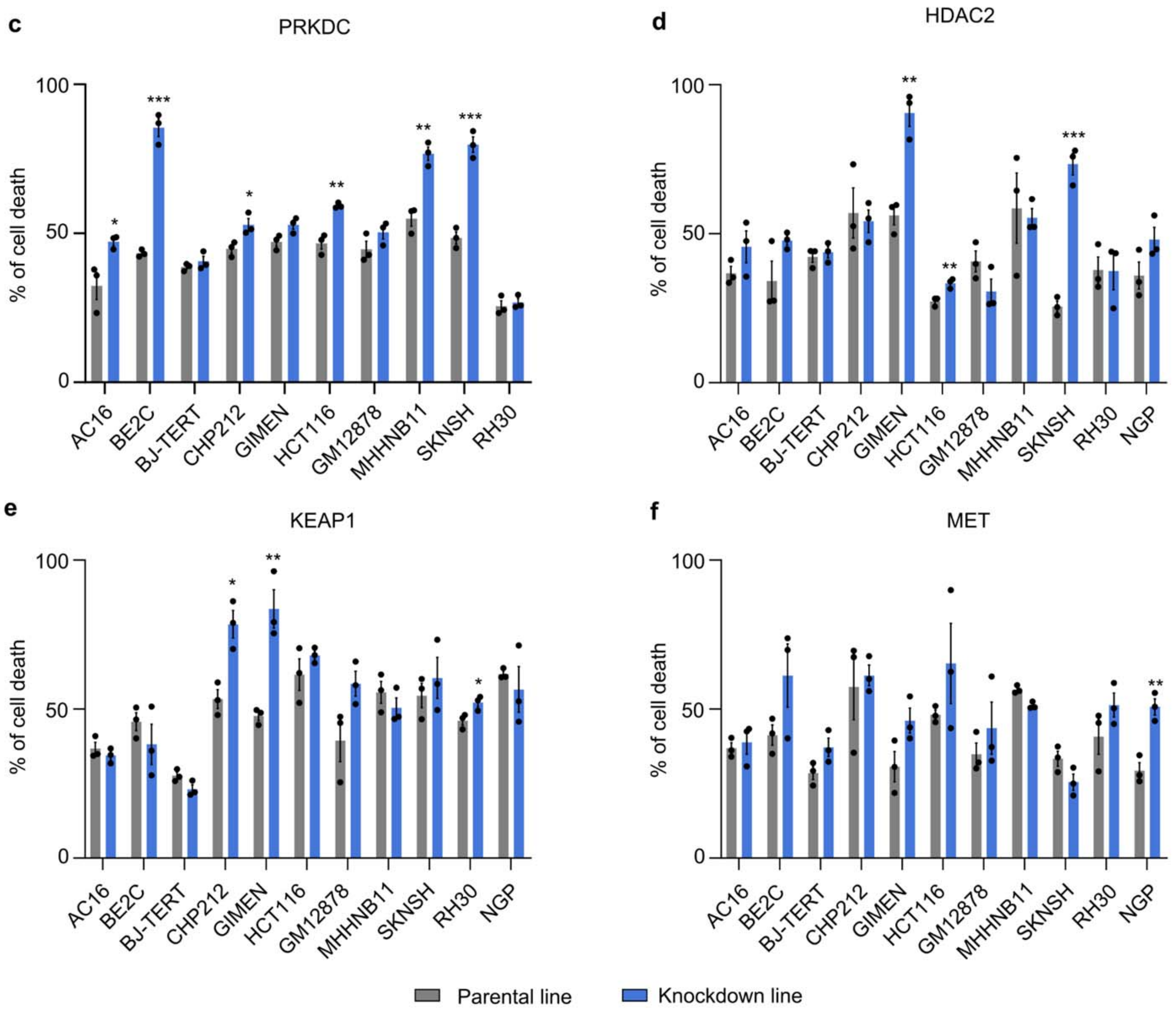

Supplementary Figure 3. Drug responses in selected parental and associated shRNA knockdown cell lines.

(a) Knockdown efficiency of individual shRNAs per gene. shPRKDC #1, shHDAC2 #1, shKEAP1 #2, and shMET #2 were used for subsequent drug response profiling (c), (d), (e), and (f), respectively. Uncroppbed blots are provided as a Source file.

(b) Knockdown of individual genes in individual cell lines. PRKDC (top left, corresponding (c)), HDAC2 (top right, corresponding to (d)), KEPA1 (bottom left, corresponding to (e)), MET (bottom right, corresponding to (f)). Uncropped blots are provided as a Source Data file. Confirmed knockdown cell lines were directly used for subsequent drug response profiling.

(c) The response of 10 parental lines (grey) and associated PRKDC knockdown cell lines (blue) to doxorubicin (~IC50). Six knockdown cell lines were sensitized to doxorubicin, suggesting synergy. AC16: P=0.034, BE2C: P=0.00016, CHP212: P=0.033, HCT116: P=0.0033, MHHNB11: P=0.0032, SKNSH: P=0.00065 (Data presented as mean ±SEM, unpaired t-test (two-side), n=3 per group, two independent. *** P < 0.001, ** P < 0.01, * P < 0.05).
(d) The response of 11 parental lines (grey) and associated HDAC2 knockdown cell lines (blue) to JQAD1 (~IC50 or capped 10μM). Three knockdown cell lines were sensitized JQAD1, suggesting synergy. GIMEN: P=0.0033, HCT116: P=0.0096, SKNSH: P=0.00028 (Data presented as mean ±SEM, unpaired t-test (two-side), n=3 per group, two independent. *** P < 0.001, ** P < 0.01, * P < 0.05).
(e) The response of 11 parental lines (grey) and associated KEAP1 knockdown cell lines (blue) to topotecan (~IC50). Three knockdown cell lines were sensitized to topotecan, suggesting synergy. CHP212: P=0.011, GIMEN: P=0.0054, RH30: P=0.04 (Data presented as mean ±SEM, unpaired t-test (two-side), n=3 per group, two independent. ** P < 0.01, * P < 0.05).
(f) The response of 11 parental lines (grey) and associated MET knockdown cell lines (blue) to cisplatin (~IC50 or capped 10μM). One knockdown cell line was sensitized to cisplatin, suggesting synergy. NGP: P=0.0048 (Data presented as mean ±SEM, unpaired t-test (two-side), n=3 per group, two independent.** P < 0.01). In (c-f) x-axis indicates cell lines and y-axis indicates % of cell death obtained by Cell-Titer Glo assay.

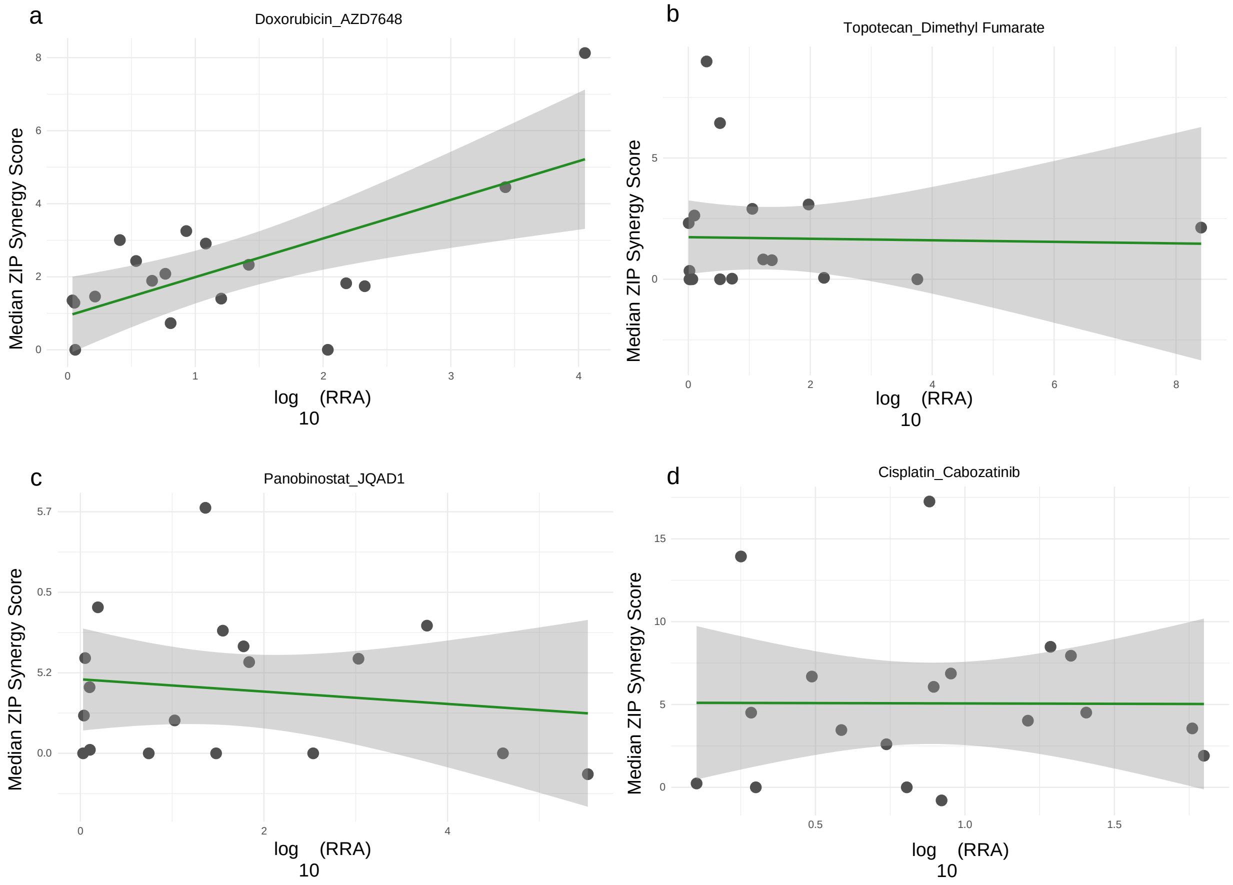

Supplementary Figure 4. Comparison of the effect of knockout in pooled CRISPR screen to synergy scores in drug combinations

Pearson correlation between -Log10(RRA|neg) and median ZIP in PRKDCi (a), KEAP1i (b), HDAC2i (c), and METi (d). Associated data sets are “Extended Data Table 11 for F5k and others”. Green line was plotted based on Pearson correlation. Shade bands are pointwise 95% CI.

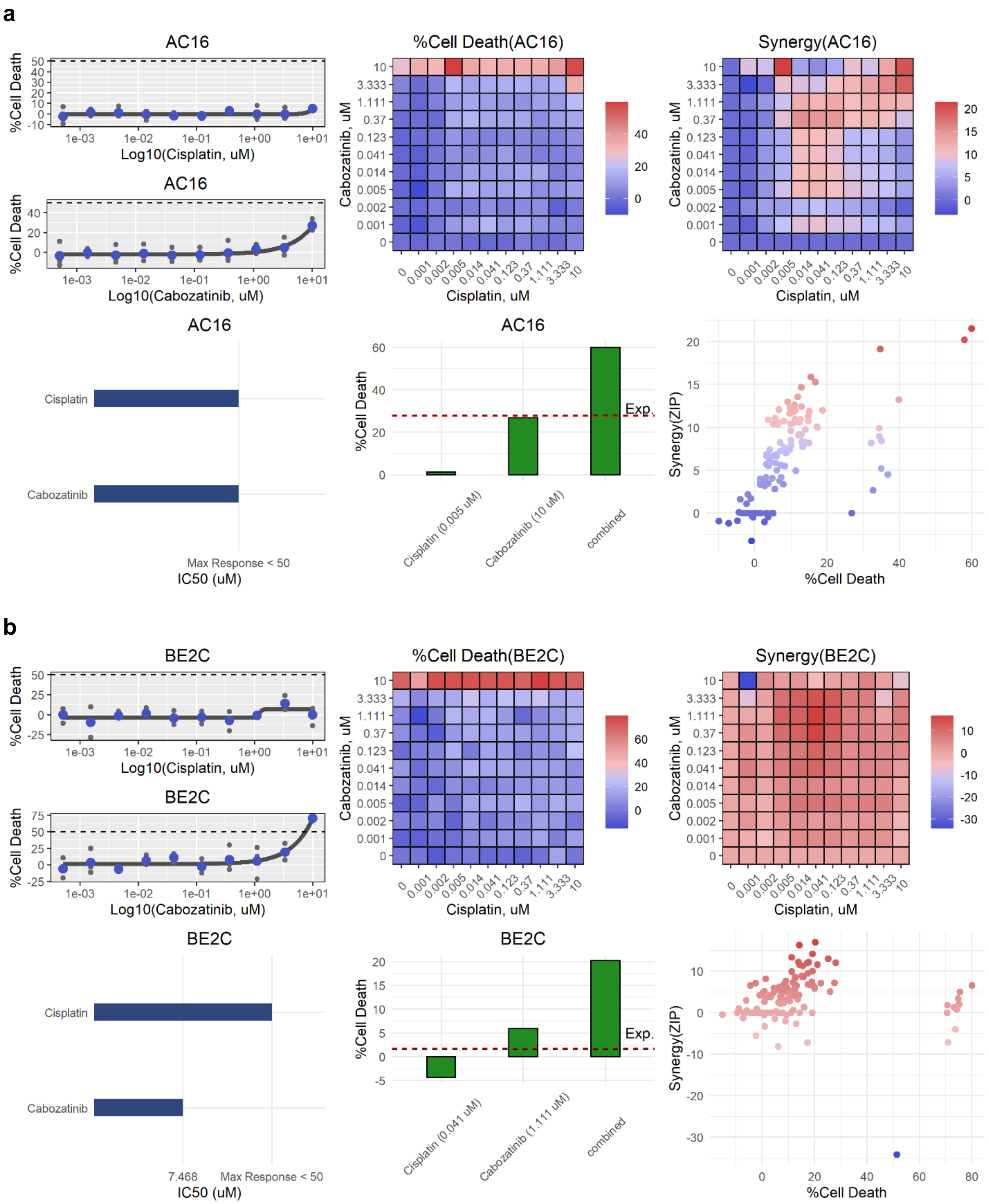
a
AC16
%Cell Death
Log10(Cisplatin, uM)
Log10(Cabozatinib, uM)
%Cell Death(AC16)
Synergy(AC16)
Cabozatinib, uM
Cisplatin, uM
IC50 (uM)
Max Response < 50
Cisplatin
Cabozatinib
Cisplatin (0.005 uM)
Cabozatinib (10 uM)
combined
Exp.
Synergy(ZIP)
%Cell Death
b
BE2C
%Cell Death(BE2C)
Synergy(BE2C)
Cisplatin (0.041 uM)
Cabozatinib (1.111 uM)
7.468
Max Response < 50

c

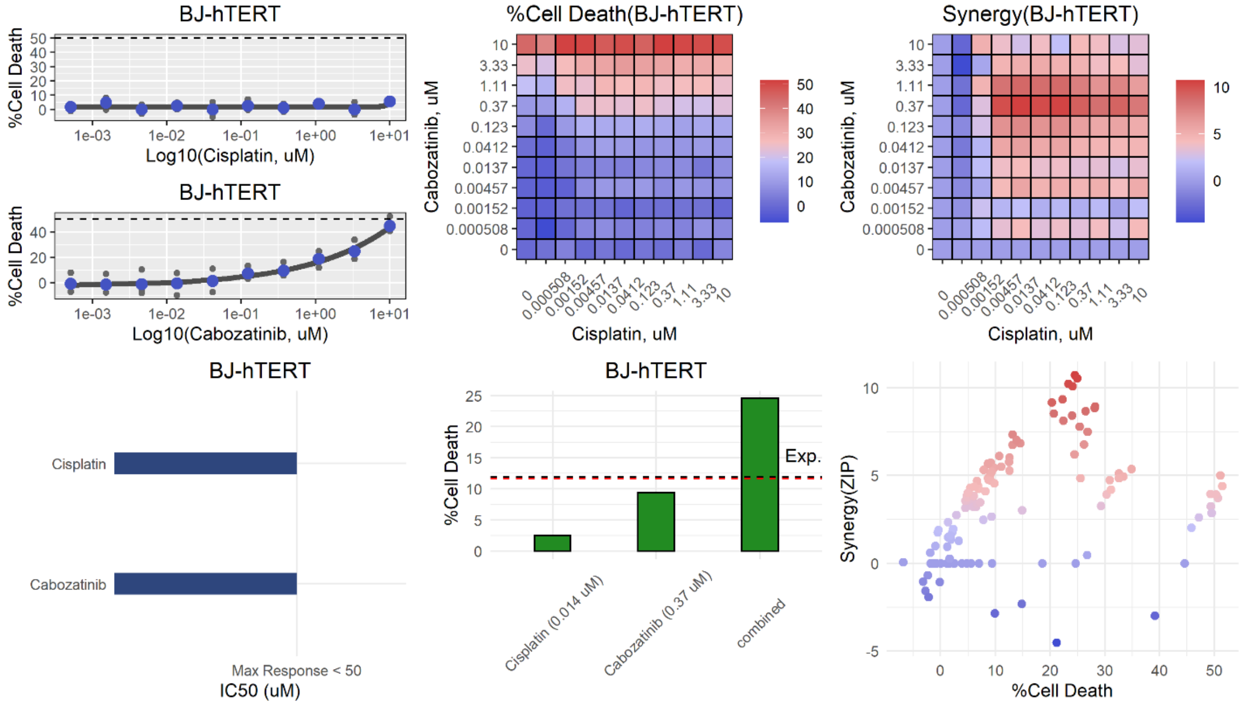
BJ-hTERT
%Cell Death
Log10(Cisplatin, uM)
BJ-hTERT
%Cell Death
Log10(Cabozatinib, uM)
%Cell Death(BJ-hTERT)
Cabozatinib, uM
Cisplatin, uM
Synergy(BJ-hTERT)
Cabozatinib, uM
Cisplatin, uM
BJ-hTERT
Cisplatin
Cabozatinib
Max Response < 50
IC50 (uM)
BJ-hTERT
%Cell Death
Exp.
Cisplatin (0.014 uM)
Cabozatinib (0.37 uM)
combined
Synergy(ZIP)
%Cell Death

d

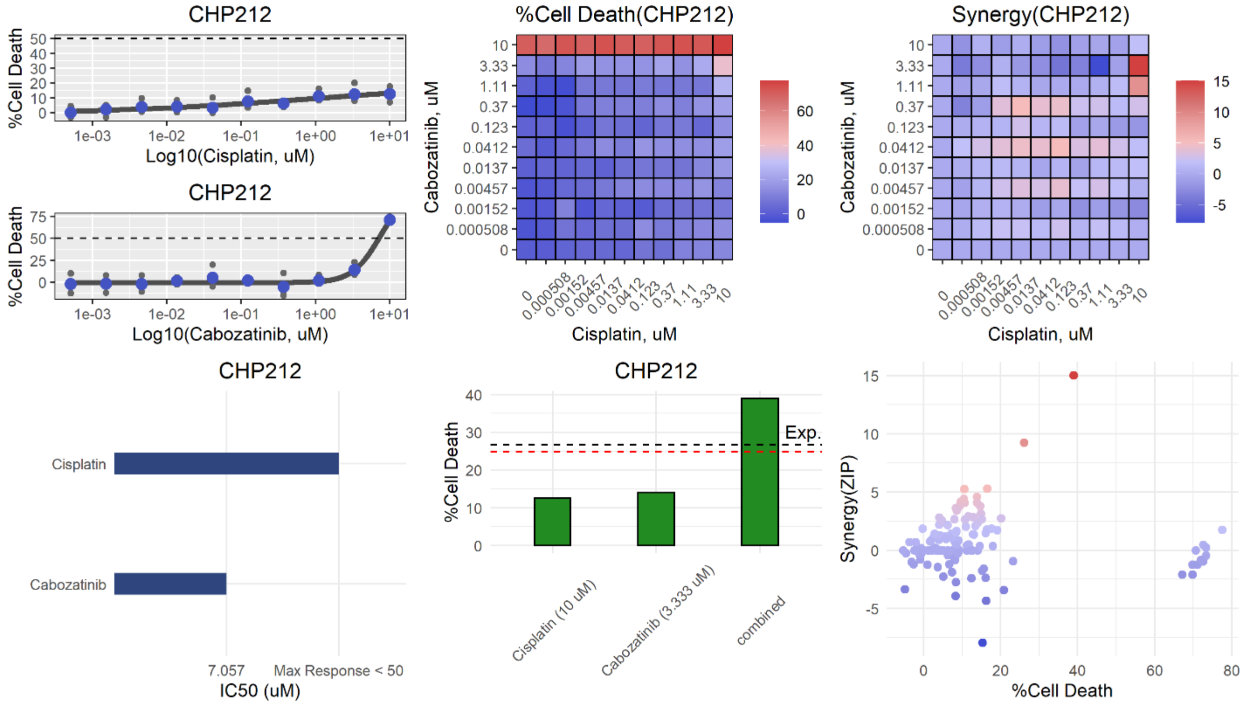
CHP212
%Cell Death
Log10(Cisplatin, uM)
CHP212
%Cell Death
Log10(Cabozatinib, uM)
%Cell Death(CHP212)
Cabozatinib, uM
Cisplatin, uM
Synergy(CHP212)
Cabozatinib, uM
Cisplatin, uM
CHP212
Cisplatin
Cabozatinib
7.057
Max Response < 50
IC50 (uM)
CHP212
%Cell Death
Exp.
Cisplatin (10 uM)
Cabozatinib (3.333 uM)
combined
Synergy(ZIP)
%Cell Death

e

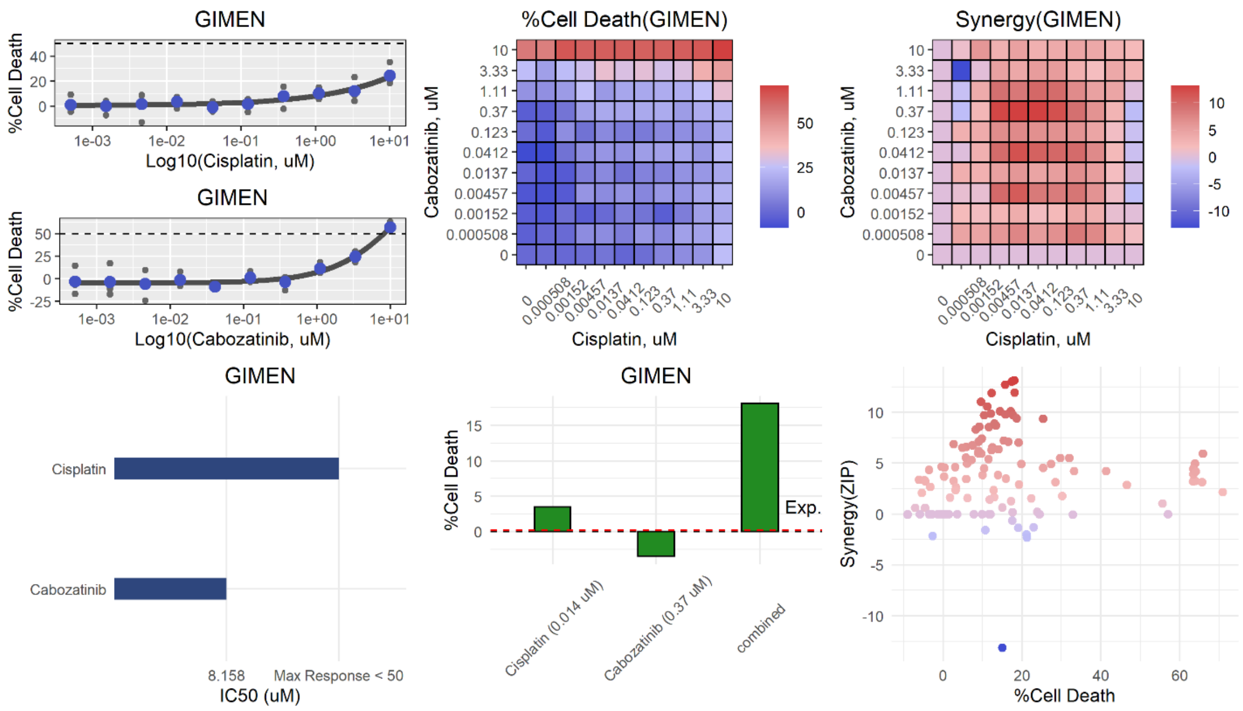
GIMEN
%Cell Death
Log10(Cisplatin, uM)
Log10(Cabozatinib, uM)
%Cell Death(GIMEN)
Synergy(GIMEN)
Cabozatinib, uM
Cisplatin, uM
Cisplatin
Cabozatinib
8.158
Max Response < 50
IC50 (uM)
Cisplatin (0.014 uM)
Cabozatinib (0.37 uM)
combined
Exp.
Synergy(ZIP)
%Cell Death

f

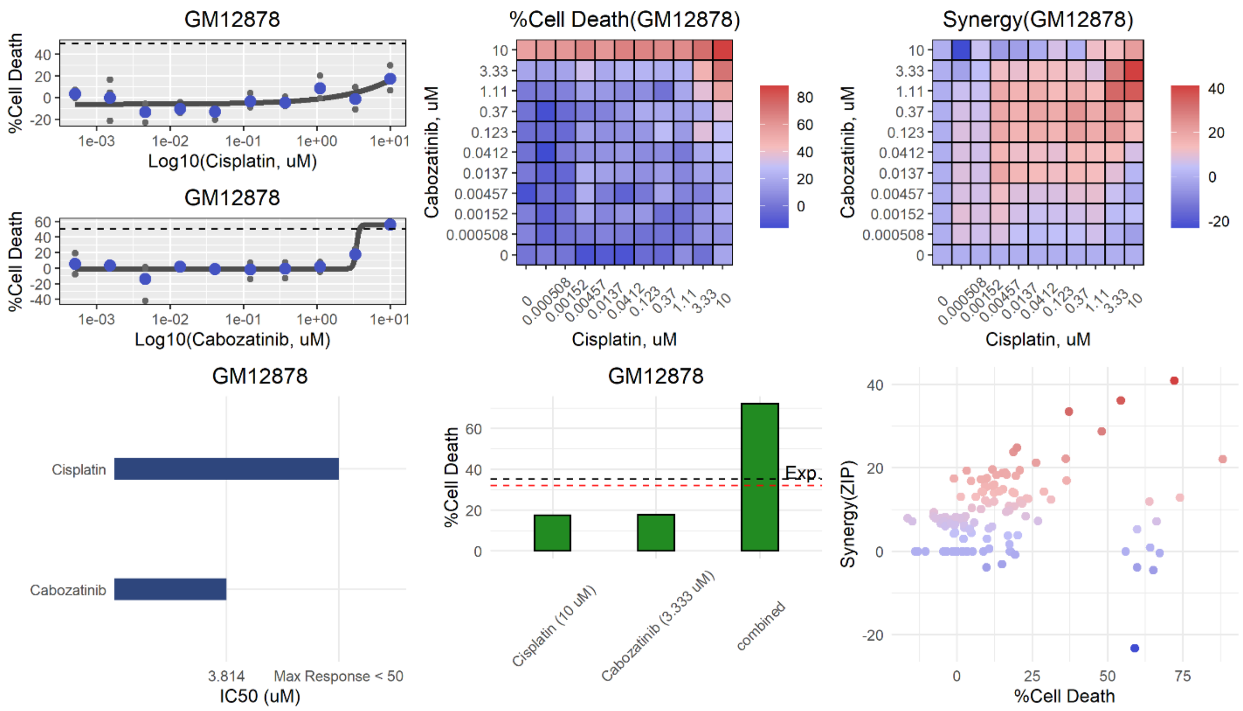
GM12878
%Cell Death
Log10(Cisplatin, uM)
Log10(Cabozatinib, uM)
%Cell Death(GM12878)
Synergy(GM12878)
Cabozatinib, uM
Cisplatin, uM
Cisplatin
Cabozatinib
3.814
Max Response < 50
IC50 (uM)
Cisplatin (10 uM)
Cabozatinib (3.333 uM)
combined
Exp.
Synergy(ZIP)
%Cell Death

g

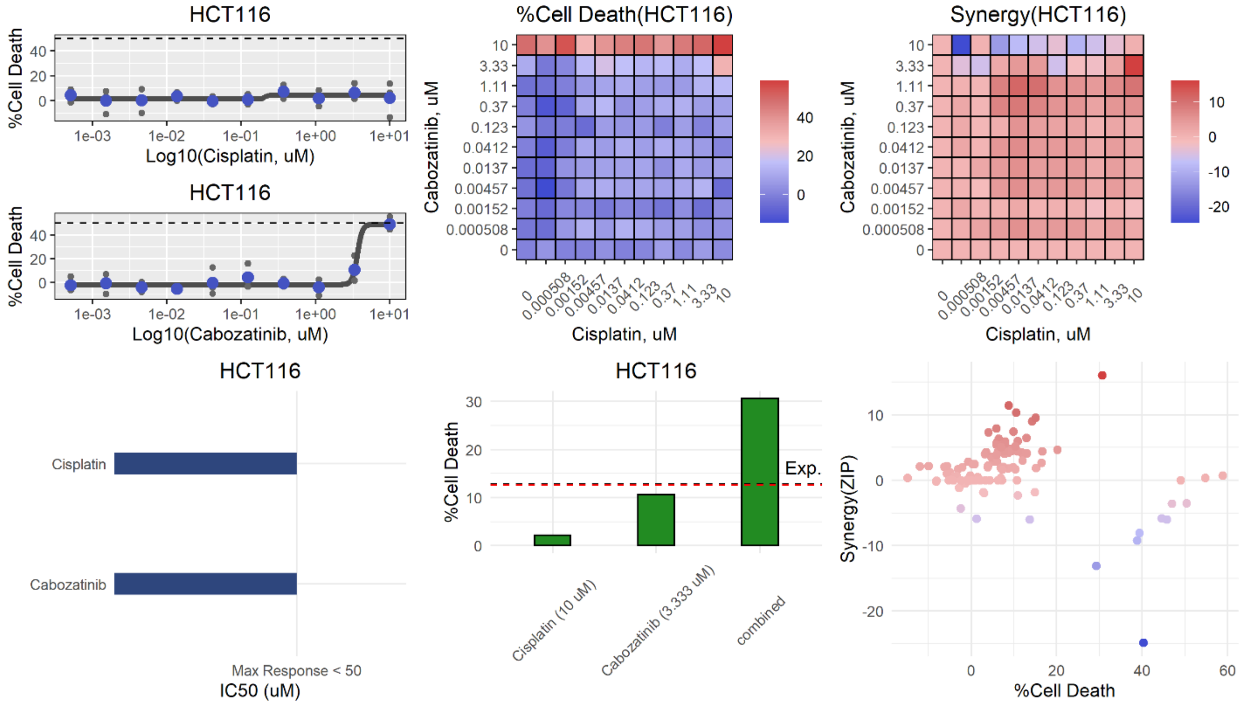
HCT116
%Cell Death
Log10(Cisplatin, uM)
HCT116
%Cell Death
Log10(Cabozatinib, uM)
%Cell Death(HCT116)
Cabozatinib, uM
Cisplatin, uM
Synergy(HCT116)
Cabozatinib, uM
Cisplatin, uM
HCT116
Cisplatin
Cabozatinib
Max Response < 50
IC50 (uM)
HCT116
%Cell Death
Exp.
Cisplatin (10 uM)
Cabozatinib (3.333 uM)
combined
Synergy(ZIP)
%Cell Death

h

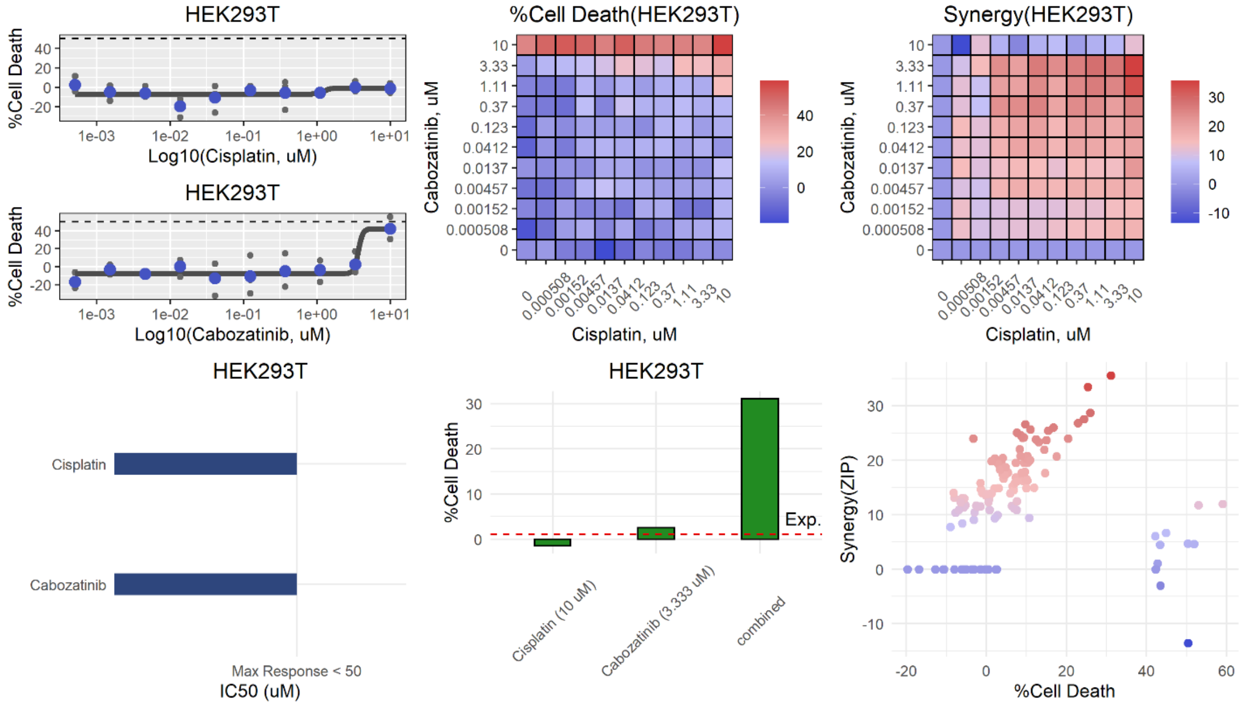
HEK293T
%Cell Death
Log10(Cisplatin, uM)
HEK293T
%Cell Death
Log10(Cabozatinib, uM)
%Cell Death(HEK293T)
Cabozatinib, uM
Cisplatin, uM
Synergy(HEK293T)
Cabozatinib, uM
Cisplatin, uM
HEK293T
Cisplatin
Cabozatinib
Max Response < 50
IC50 (uM)
HEK293T
%Cell Death
Exp.
Cisplatin (10 uM)
Cabozatinib (3.333 uM)
combined
Synergy(ZIP)
%Cell Death

i

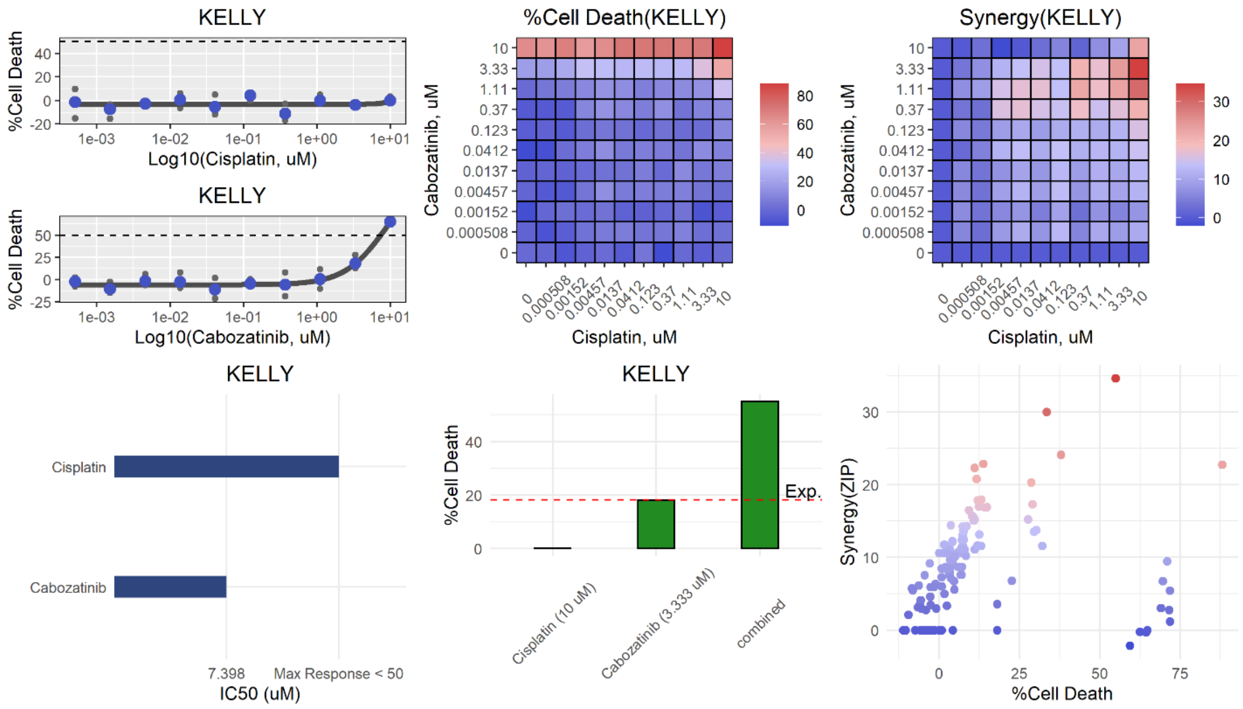

KELLY
%Cell Death
Log10(Cisplatin, uM)
Log10(Cabozatinib, uM)
1e-03
1e-02
1e-01
1e+00
1e+01
%Cell Death(KELLY)
Synergy(KELLY)
Cabozatinib, uM
Cisplatin, uM
0
0.000508
0.00152
0.00457
0.0137
0.0412
0.123
0.37
1.11
3.33
10
Cisplatin
Cabozatinib
7.398
Max Response < 50
IC50 (uM)
Cisplatin (10 uM)
Cabozatinib (3.333 uM)
combined
Exp.
Synergy(ZIP)
%Cell Death

j

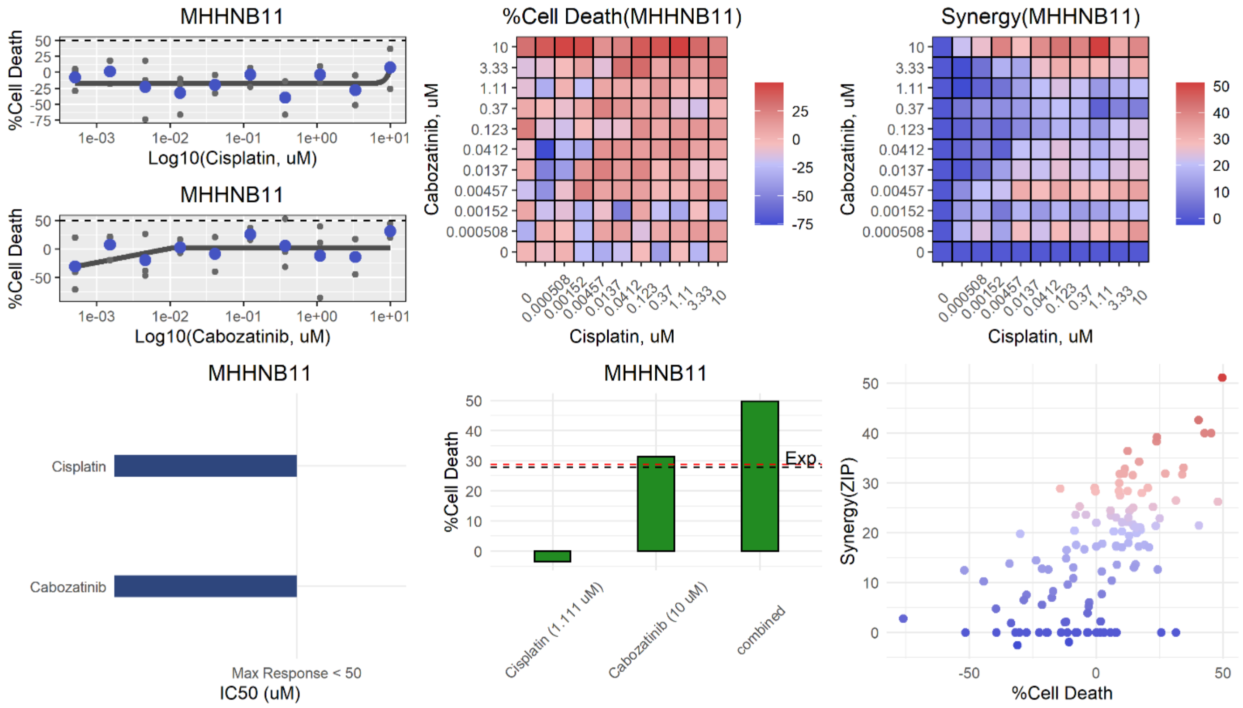

MHHNB11
%Cell Death
Log10(Cisplatin, uM)
Log10(Cabozatinib, uM)
1e-03
1e-02
1e-01
1e+00
1e+01
%Cell Death(MHHNB11)
Synergy(MHHNB11)
Cabozatinib, uM
Cisplatin, uM
0
0.000508
0.00152
0.00457
0.0137
0.0412
0.123
0.37
1.11
3.33
10
Cisplatin
Cabozatinib
Max Response < 50
IC50 (uM)
Cisplatin (1.111 uM)
Cabozatinib (10 uM)
combined
Exp.
Synergy(ZIP)
%Cell Death

k

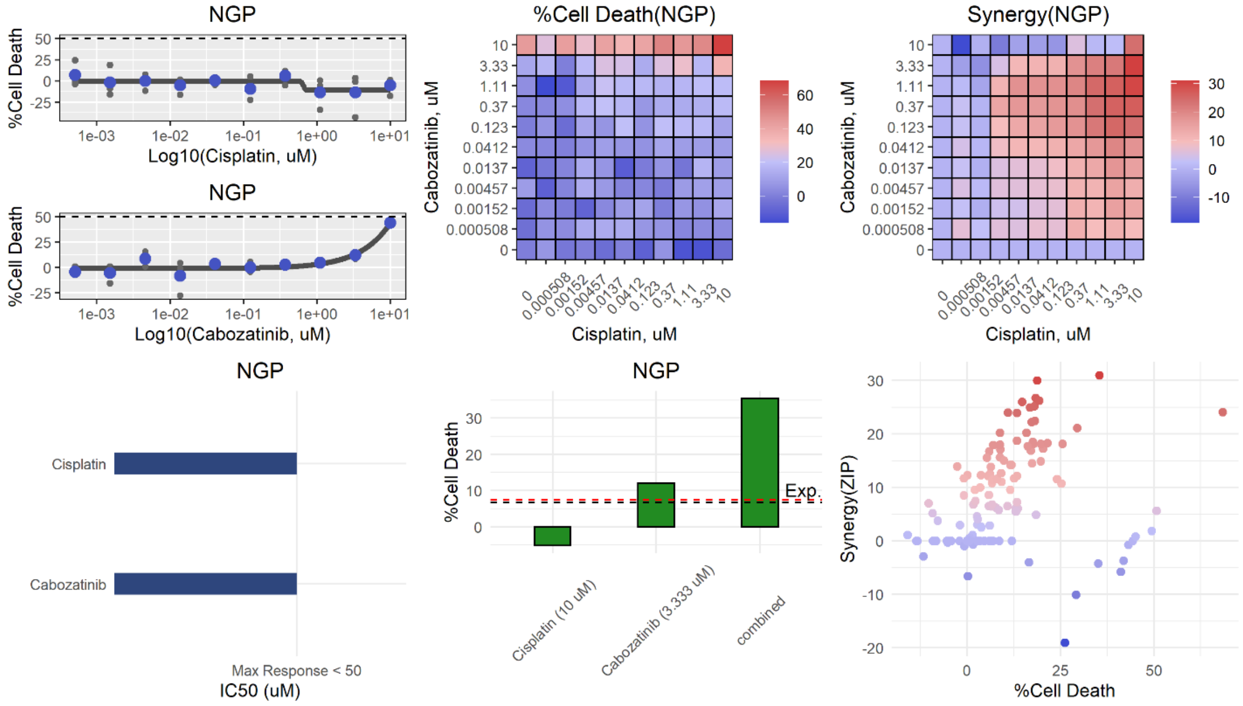
NGP
%Cell Death
Log10(Cisplatin, uM)
NGP
%Cell Death
Log10(Cabozatinib, uM)
%Cell Death(NGP)
Cabozatinib, uM
Cisplatin, uM
Synergy(NGP)
Cabozatinib, uM
Cisplatin, uM
NGP
Cisplatin
Cabozatinib
Max Response < 50
IC50 (uM)
NGP
%Cell Death
Exp.
Cisplatin (10 uM)
Cabozatinib (3.333 uM)
combined
Synergy(ZIP)
%Cell Death

l

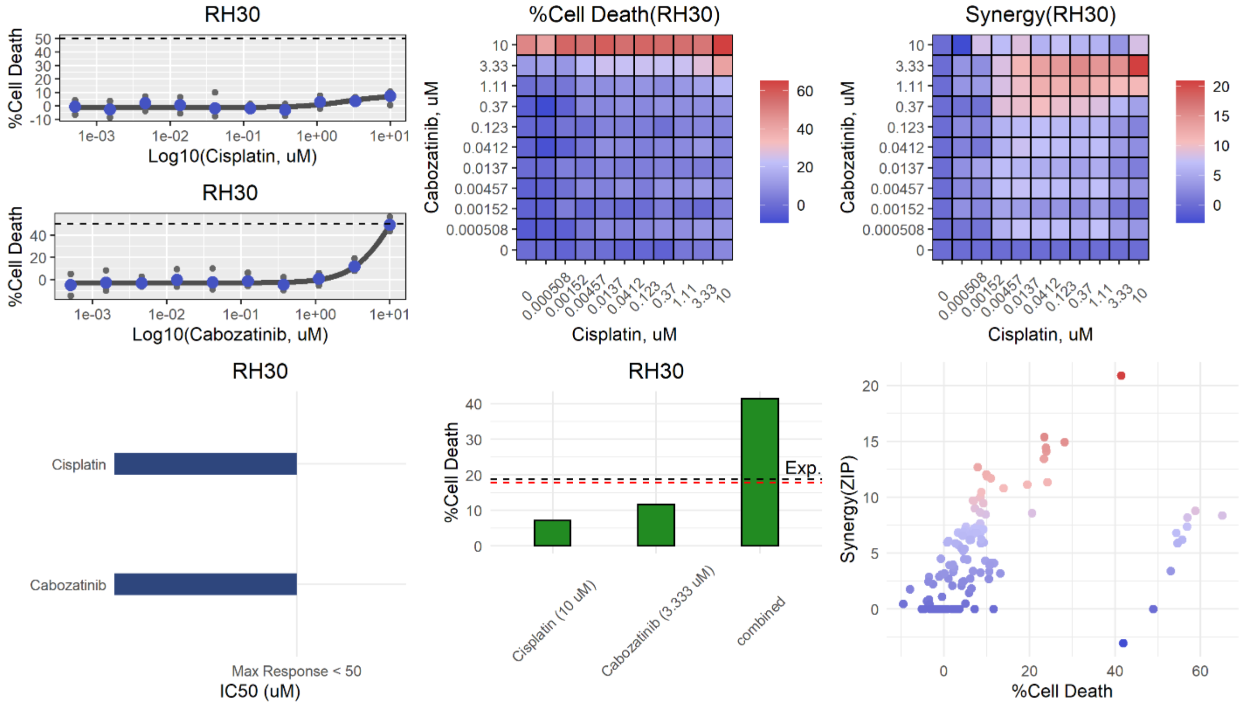
RH30
%Cell Death
Log10(Cisplatin, uM)
RH30
%Cell Death
Log10(Cabozatinib, uM)
%Cell Death(RH30)
Cabozatinib, uM
Cisplatin, uM
Synergy(RH30)
Cabozatinib, uM
Cisplatin, uM
RH30
Cisplatin
Cabozatinib
Max Response < 50
IC50 (uM)
RH30
%Cell Death
Exp.
Cisplatin (10 uM)
Cabozatinib (3.333 uM)
combined
Synergy(ZIP)
%Cell Death

m

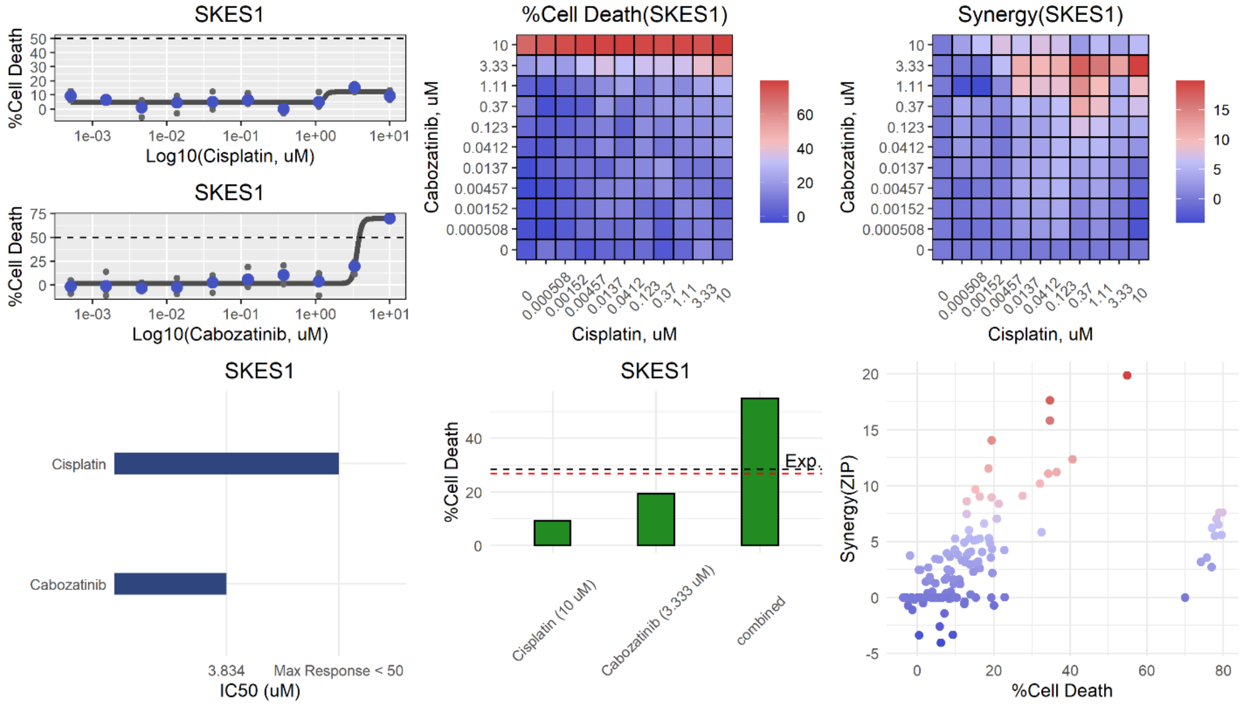
SKES1
%Cell Death
50
40
30
20
10
0
1e-03
1e-02
1e-01
1e+00
1e+01
Log10(Cisplatin, uM)
SKES1
%Cell Death
75
50
25
0
1e-03
1e-02
1e-01
1e+00
1e+01
Log10(Cabozatinib, uM)
%Cell Death(SKES1)
Cabozatinib, uM
10
3.33
1.11
0.37
0.123
0.0412
0.0137
0.00457
0.00152
0.000508
0
60
40
20
0
0
0.000508
0.00152
0.00457
0.0137
0.0412
0.123
0.37
1.11
3.33
10
Cisplatin, uM
Synergy(SKES1)
Cabozatinib, uM
15
10
5
0
Cisplatin, uM
SKES1
Cisplatin
Cabozatinib
3.834
Max Response < 50
IC50 (uM)
SKES1
%Cell Death
40
20
0
Exp.
Cisplatin (10 uM)
Cabozatinib (3.333 uM)
combined
Synergy(ZIP)
20
15
10
5
0
-5
0
20
40
60
80
%Cell Death

n

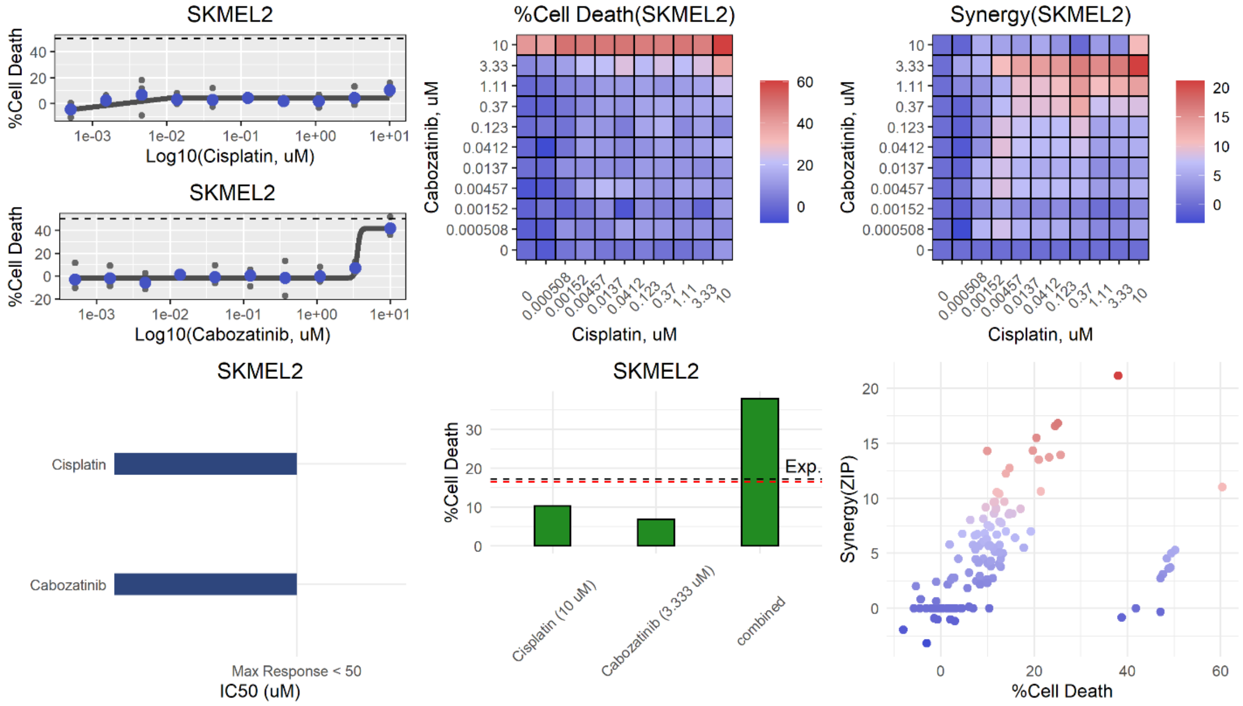
SKMEL2
%Cell Death
40
20
0
1e-03
1e-02
1e-01
1e+00
1e+01
Log10(Cisplatin, uM)
SKMEL2
%Cell Death
40
20
0
-20
1e-03
1e-02
1e-01
1e+00
1e+01
Log10(Cabozatinib, uM)
%Cell Death(SKMEL2)
Cabozatinib, uM
10
3.33
1.11
0.37
0.123
0.0412
0.0137
0.00457
0.00152
0.000508
0
60
40
20
0
0
0.000508
0.00152
0.00457
0.0137
0.0412
0.123
0.37
1.11
3.33
10
Cisplatin, uM
Synergy(SKMEL2)
Cabozatinib, uM
20
15
10
5
0
Cisplatin, uM
SKMEL2
Cisplatin
Cabozatinib
Max Response < 50
IC50 (uM)
SKMEL2
%Cell Death
30
20
10
0
Exp.
Cisplatin (10 uM)
Cabozatinib (3.333 uM)
combined
Synergy(ZIP)
20
15
10
5
0
0
20
40
60
%Cell Death

o

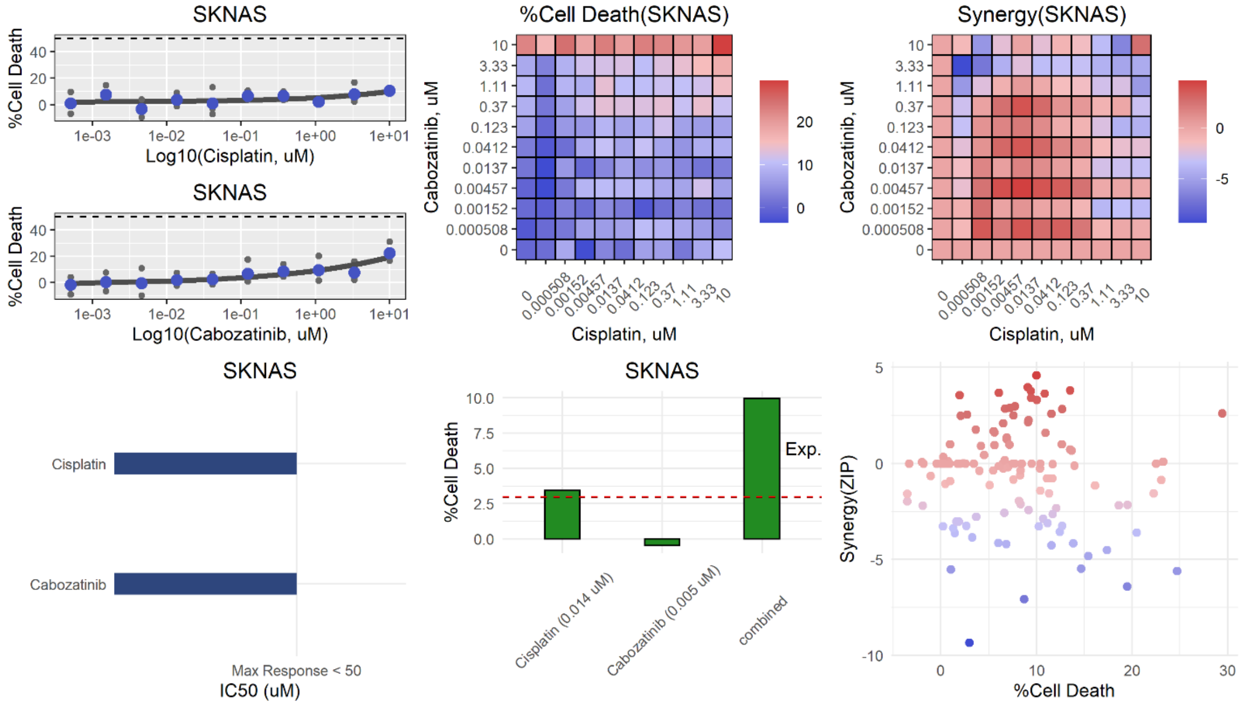
SKNAS
%Cell Death
Log10(Cisplatin, uM)
SKNAS
%Cell Death
Log10(Cabozatinib, uM)
%Cell Death(SKNAS)
Cabozatinib, uM
Cisplatin, uM
Synergy(SKNAS)
Cabozatinib, uM
Cisplatin, uM
SKNAS
Cisplatin
Cabozatinib
Max Response < 50
IC50 (uM)
SKNAS
%Cell Death
Exp.
Cisplatin (0.014 uM)
Cabozatinib (0.005 uM)
combined
Synergy(ZIP)
%Cell Death

p

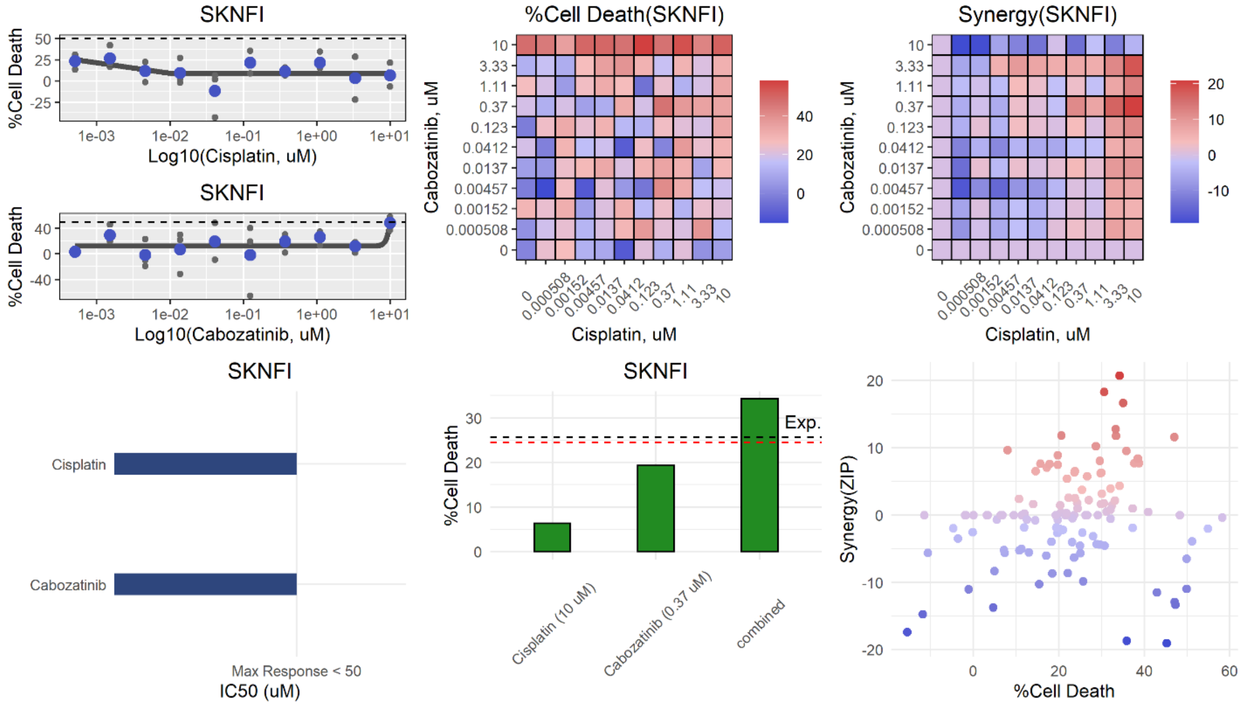
SKNFI
%Cell Death
Log10(Cisplatin, uM)
SKNFI
%Cell Death
Log10(Cabozatinib, uM)
%Cell Death(SKNFI)
Cabozatinib, uM
Cisplatin, uM
Synergy(SKNFI)
Cabozatinib, uM
Cisplatin, uM
SKNFI
Cisplatin
Cabozatinib
Max Response < 50
IC50 (uM)
SKNFI
%Cell Death
Exp.
Cisplatin (10 uM)
Cabozatinib (0.37 uM)
combined
Synergy(ZIP)
%Cell Death

q

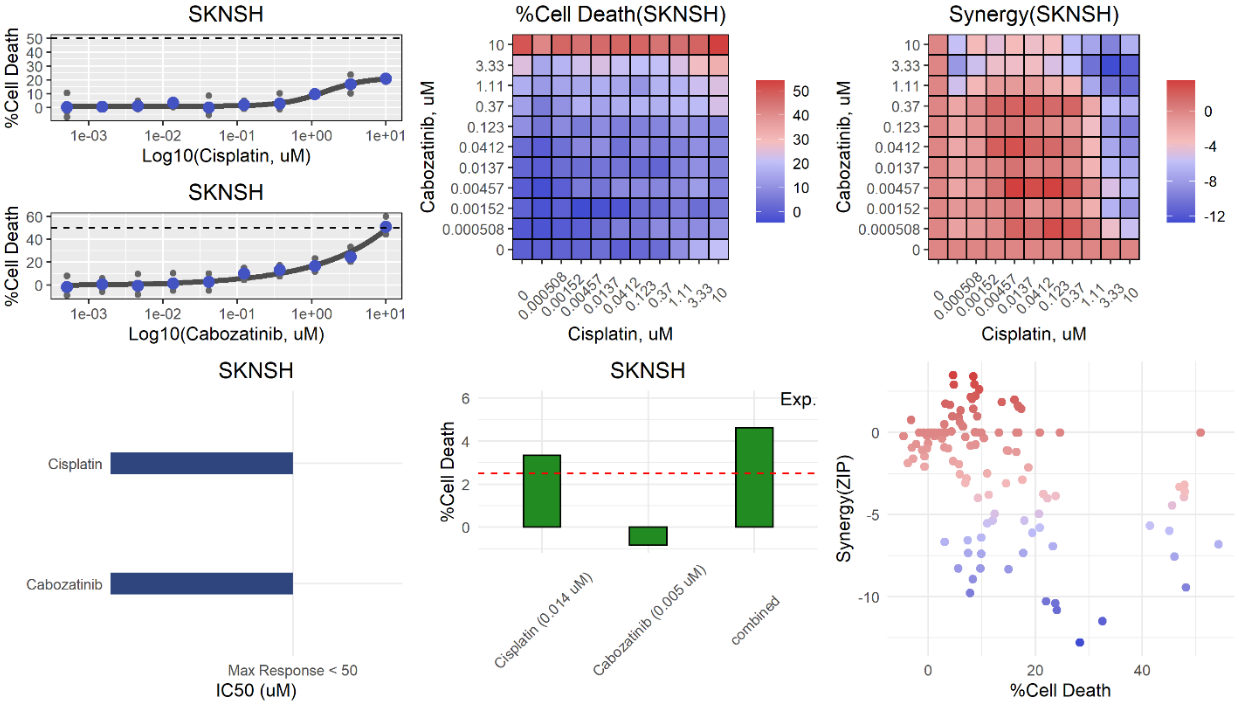
SKNSH
%Cell Death
Log10(Cisplatin, uM)
Log10(Cabozatinib, uM)
%Cell Death(SKNSH)
Synergy(SKNSH)
Cabozatinib, uM
Cisplatin, uM
Cisplatin
Cabozatinib
Max Response < 50
IC50 (uM)
Exp.
Cisplatin (0.014 uM)
Cabozatinib (0.005 uM)
combined
Synergy(ZIP)
%Cell Death

r

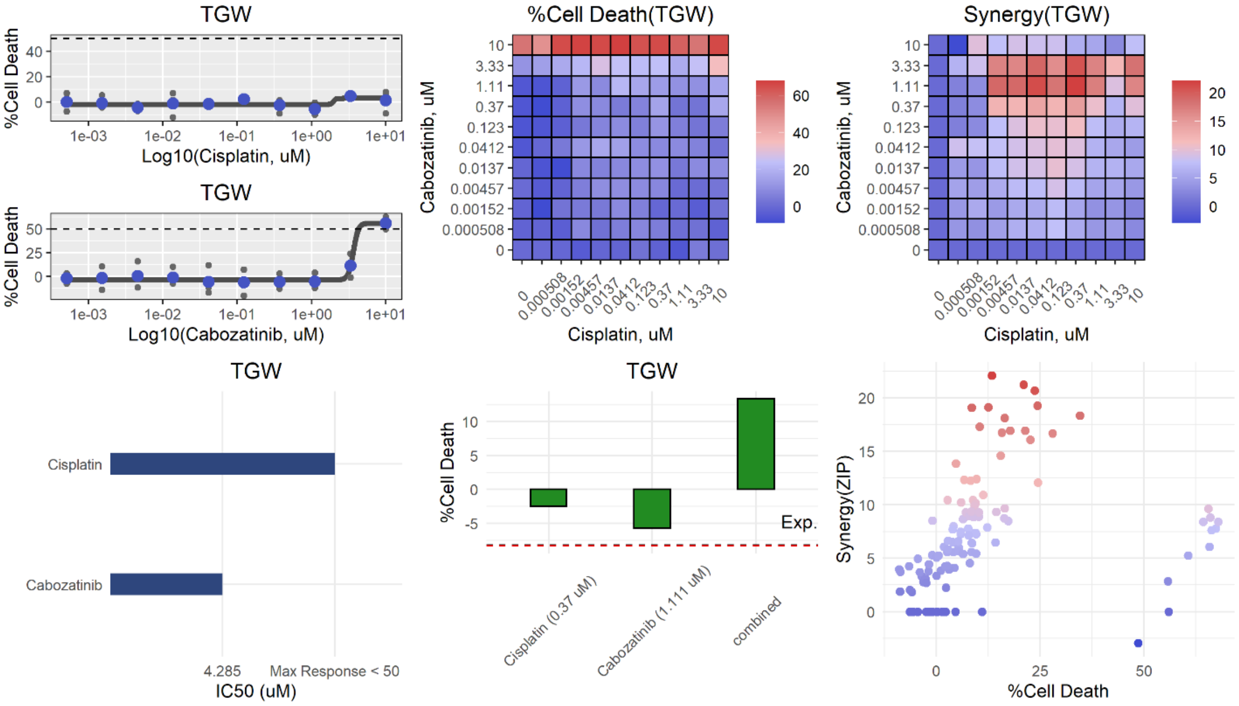
TGW
%Cell Death
Log10(Cisplatin, uM)
Log10(Cabozatinib, uM)
%Cell Death(TGW)
Synergy(TGW)
Cabozatinib, uM
Cisplatin, uM
Cisplatin
Cabozatinib
4.285
Max Response < 50
IC50 (uM)
Exp.
Cisplatin (0.37 uM)
Cabozatinib (1.111 uM)
combined
Synergy(ZIP)
%Cell Death

Supplementary Figure 5. Summary of dense cisplatin-cabozantinib combinations in all cell lines.

(a - r) 18 panels for combination of cisplatin and cabozantinib in all 18 cell lines. a) AC16, b) BE2C, c) BJ-TERT, d) CHP212, e) GIMEN, f) GM12878, g) HCT116, h) HKE293T, i) KELLY, j) MHHNB11, k) NGP, l) RH30, m) SKES1, n) SKMEL2, o) SKNAS, p) SKNFI, q) SKNSH, r) TGW. Dose-response curves for each single-agent (top left), normalized cell death matrix (top middle), synergy matrix (top right; Note: units are δZIP), IC_{50} values of each single-agent (lower left), barplots corresponding to maximum synergy (lower middle; Note: dashed lines indicate expected values under additivity (black) or Bliss independence (red)), and scatterplot of cell death vs. synergy (lower right) for all 18 cell lines used in dense drug-drug screens.

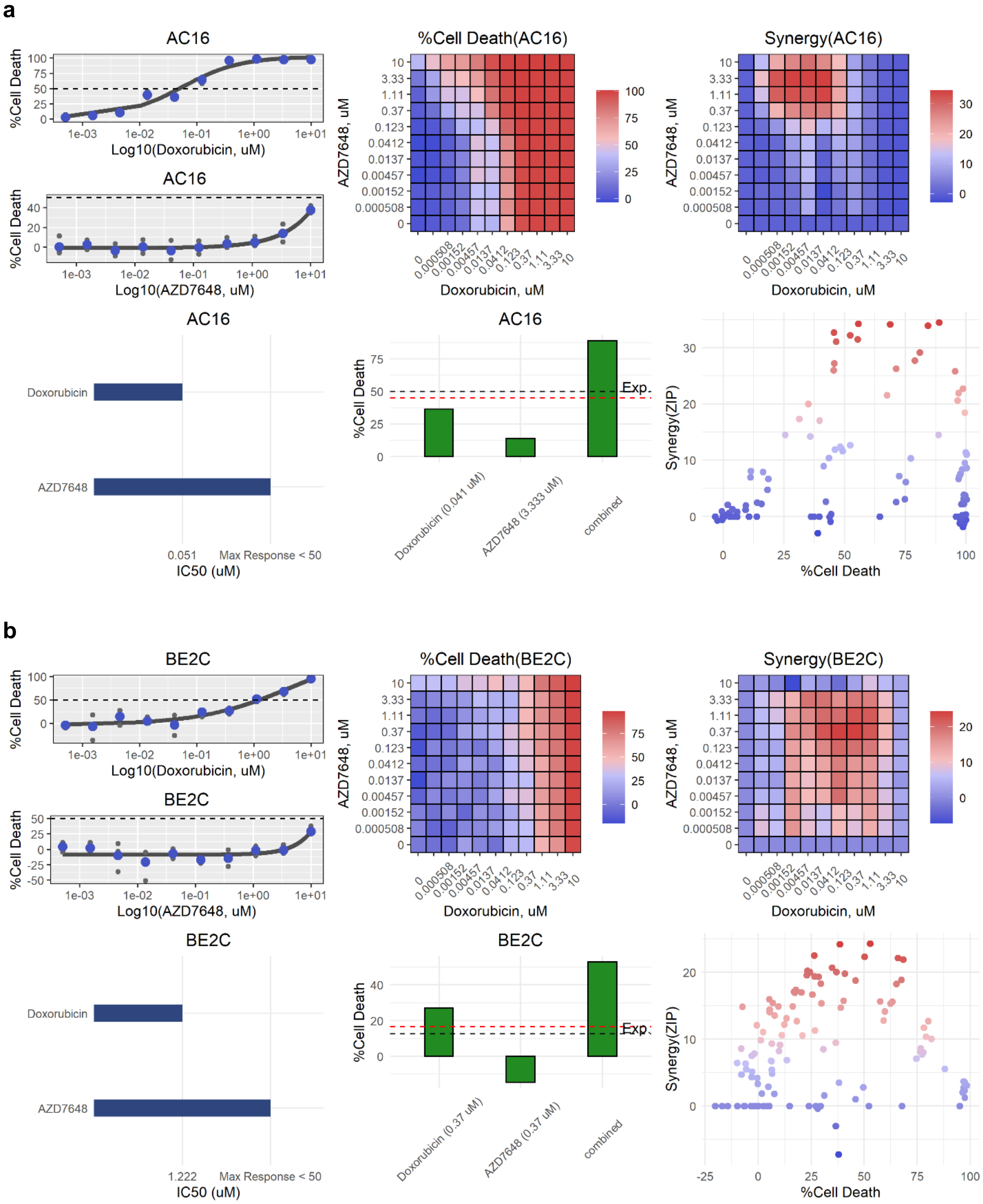

a
AC16
%Cell Death
Log10(Doxorubicin, uM)
AC16
%Cell Death
Log10(AZD7648, uM)
%Cell Death(AC16)
AZD7648, uM
Doxorubicin, uM
Synergy(AC16)
AZD7648, uM
Doxorubicin, uM
AC16
Doxorubicin
AZD7648
0.051
Max Response < 50
IC50 (uM)
AC16
%Cell Death
Exp.
Doxorubicin (0.041 uM)
AZD7648 (3.333 uM)
combined
Synergy(ZIP)
%Cell Death
b
BE2C
%Cell Death
Log10(Doxorubicin, uM)
BE2C
%Cell Death
Log10(AZD7648, uM)
%Cell Death(BE2C)
AZD7648, uM
Doxorubicin, uM
Synergy(BE2C)
AZD7648, uM
Doxorubicin, uM
BE2C
Doxorubicin
AZD7648
1.222
Max Response < 50
IC50 (uM)
BE2C
%Cell Death
Exp.
Doxorubicin (0.37 uM)
AZD7648 (0.37 uM)
combined
Synergy(ZIP)
%Cell Death

c

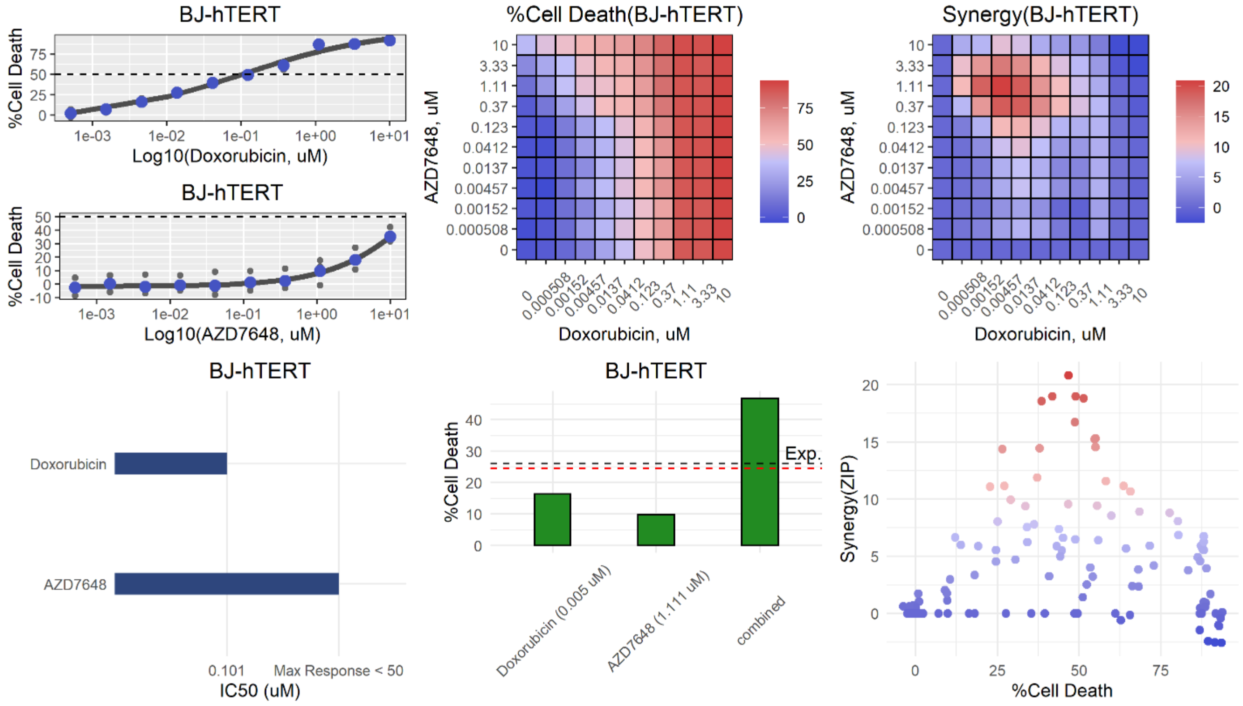
BJ-hTERT
%Cell Death
Log10(Doxorubicin, uM)
BJ-hTERT
%Cell Death
Log10(AZD7648, uM)
%Cell Death(BJ-hTERT)
AZD7648, uM
Doxorubicin, uM
Synergy(BJ-hTERT)
AZD7648, uM
Doxorubicin, uM
BJ-hTERT
Doxorubicin
AZD7648
0.101
Max Response < 50
IC50 (uM)
BJ-hTERT
%Cell Death
Exp.
Doxorubicin (0.005 uM)
AZD7648 (1.111 uM)
combined
Synergy(ZIP)
%Cell Death

d

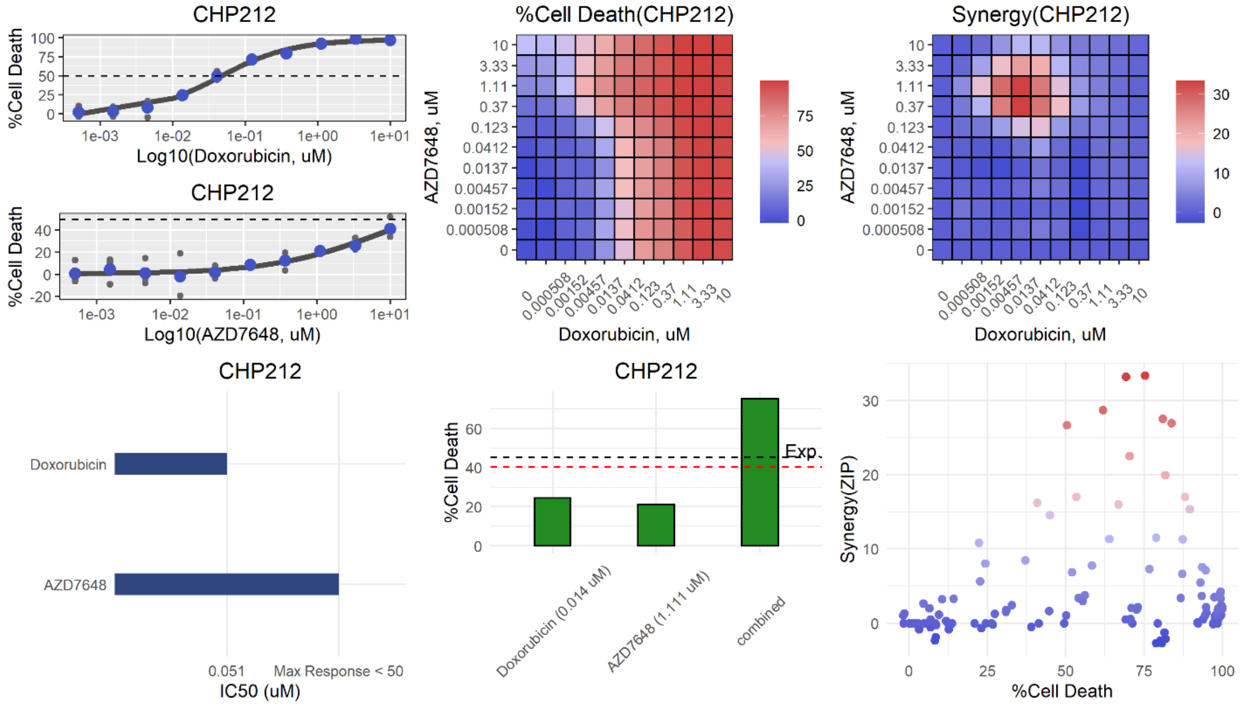
CHP212
%Cell Death
Log10(Doxorubicin, uM)
CHP212
%Cell Death
Log10(AZD7648, uM)
%Cell Death(CHP212)
AZD7648, uM
Doxorubicin, uM
Synergy(CHP212)
AZD7648, uM
Doxorubicin, uM
CHP212
Doxorubicin
AZD7648
0.051
Max Response < 50
IC50 (uM)
CHP212
%Cell Death
Exp.
Doxorubicin (0.014 uM)
AZD7648 (1.111 uM)
combined
Synergy(ZIP)
%Cell Death

e

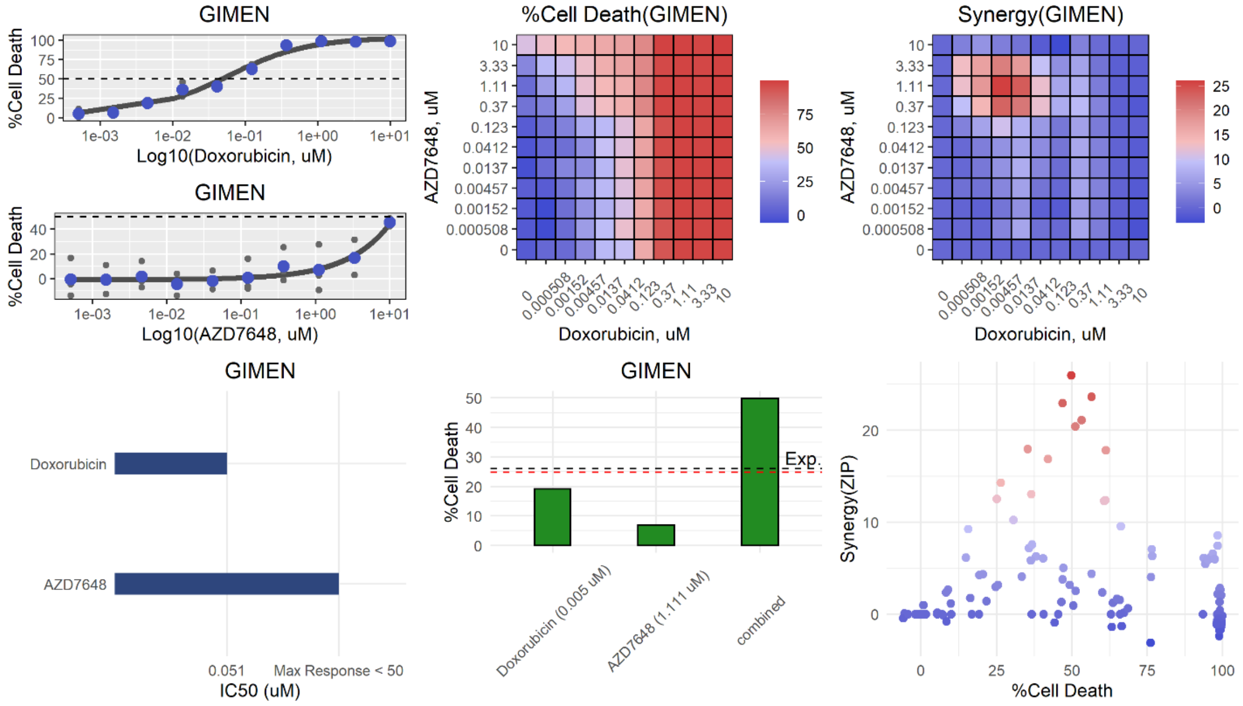
GIMEN
%Cell Death
Log10(Doxorubicin, uM)
Log10(AZD7648, uM)
%Cell Death(GIMEN)
Synergy(GIMEN)
AZD7648, uM
Doxorubicin, uM
Doxorubicin
AZD7648
0.051
Max Response < 50
IC50 (uM)
Exp.
Doxorubicin (0.005 uM)
AZD7648 (1.111 uM)
combined
Synergy(ZIP)

f

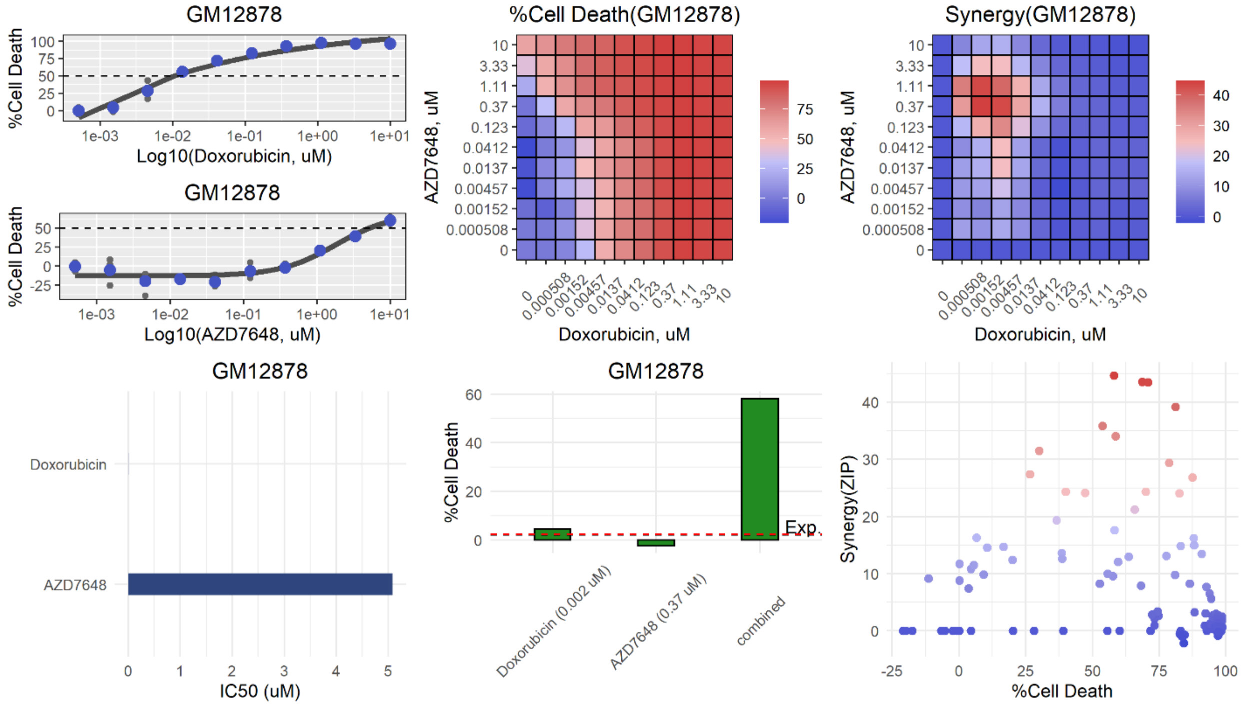
GM12878
%Cell Death
Log10(Doxorubicin, uM)
Log10(AZD7648, uM)
%Cell Death(GM12878)
Synergy(GM12878)
AZD7648, uM
Doxorubicin, uM
Doxorubicin
AZD7648
IC50 (uM)
Exp.
Doxorubicin (0.002 uM)
AZD7648 (0.37 uM)
combined
Synergy(ZIP)

g

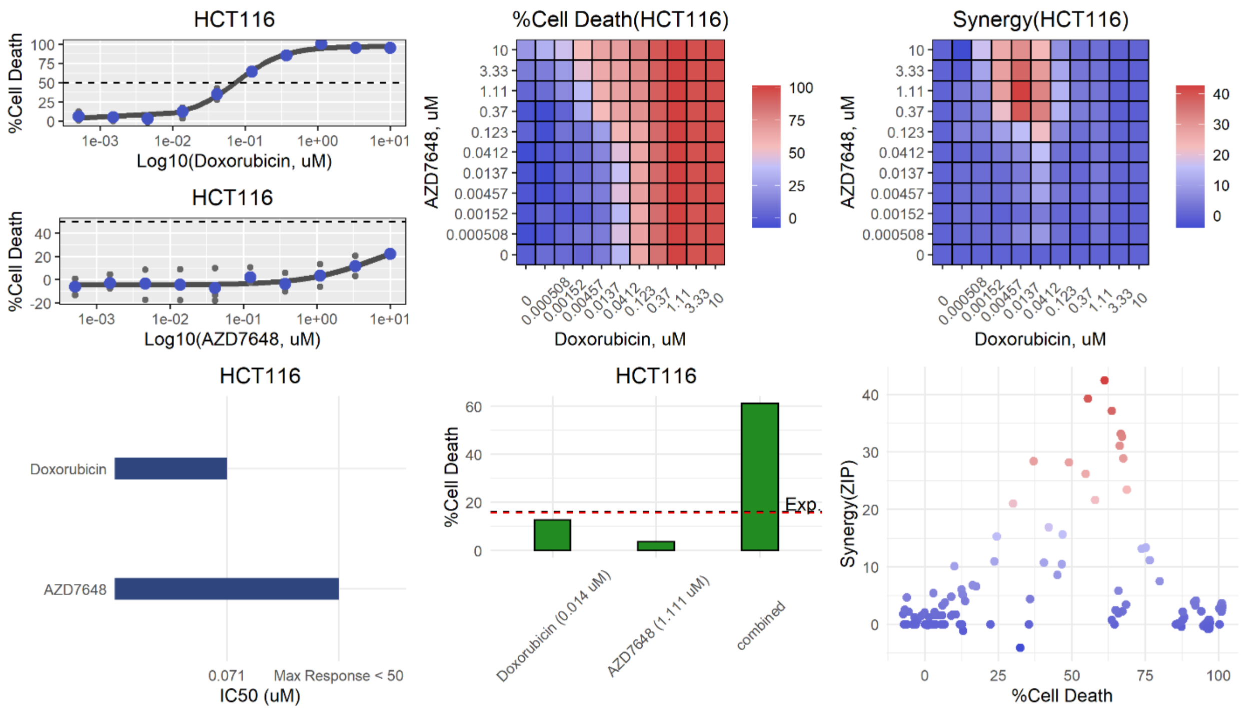
HCT116
%Cell Death
Log10(Doxorubicin, uM)
HCT116
%Cell Death
Log10(AZD7648, uM)
%Cell Death(HCT116)
AZD7648, uM
Doxorubicin, uM
Synergy(HCT116)
AZD7648, uM
Doxorubicin, uM
HCT116
Doxorubicin
AZD7648
0.071
Max Response < 50
IC50 (uM)
HCT116
%Cell Death
Exp.
Doxorubicin (0.014 uM)
AZD7648 (1.111 uM)
combined
Synergy(ZIP)
%Cell Death

h

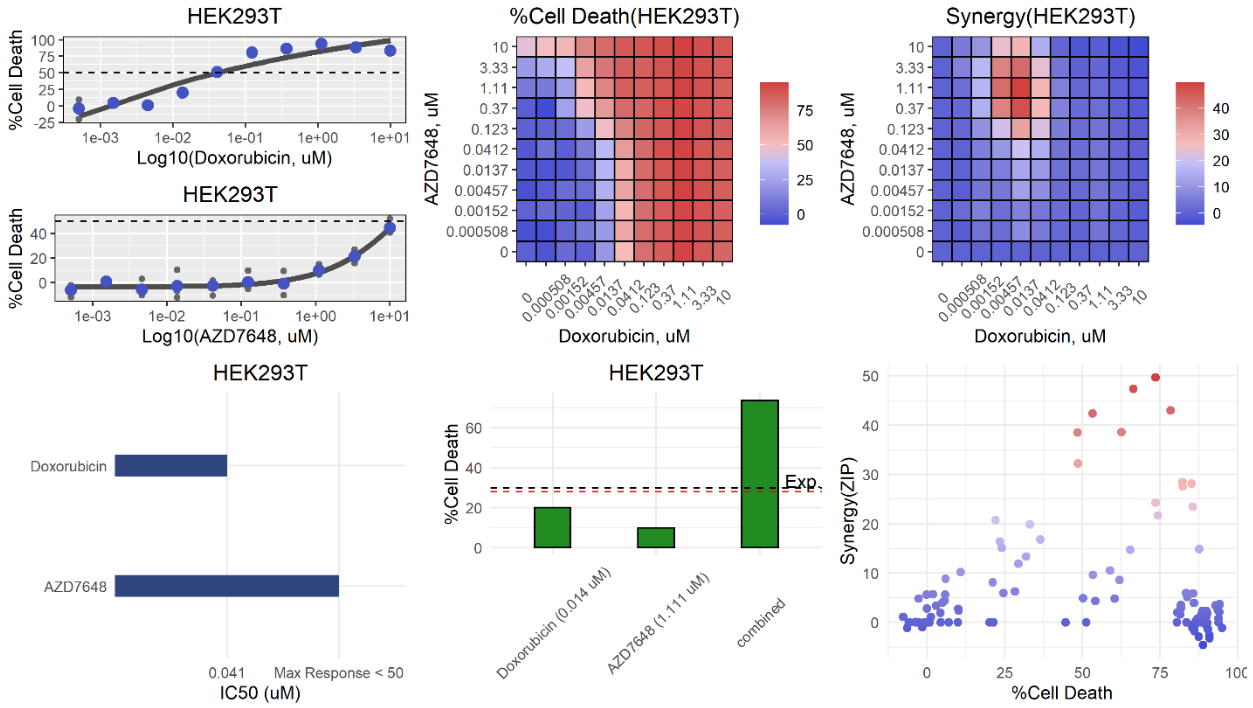
HEK293T
%Cell Death
Log10(Doxorubicin, uM)
HEK293T
%Cell Death
Log10(AZD7648, uM)
%Cell Death(HEK293T)
AZD7648, uM
Doxorubicin, uM
Synergy(HEK293T)
AZD7648, uM
Doxorubicin, uM
HEK293T
Doxorubicin
AZD7648
0.041
Max Response < 50
IC50 (uM)
HEK293T
%Cell Death
Exp.
Doxorubicin (0.014 uM)
AZD7648 (1.111 uM)
combined
Synergy(ZIP)
%Cell Death

i

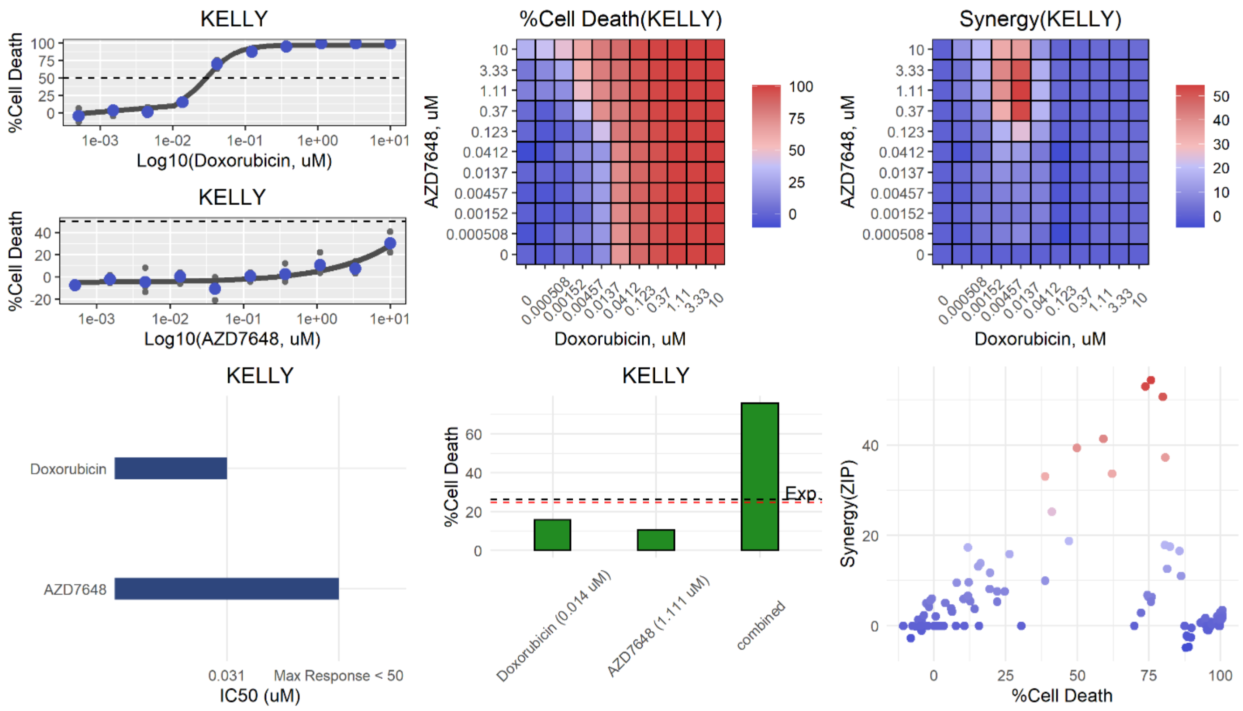
KELLY
%Cell Death
Log10(Doxorubicin, uM)
KELLY
%Cell Death
Log10(AZD7648, uM)
%Cell Death(KELLY)
AZD7648, uM
Doxorubicin, uM
Synergy(KELLY)
AZD7648, uM
Doxorubicin, uM
KELLY
Doxorubicin
AZD7648
0.031
Max Response < 50
IC50 (uM)
KELLY
%Cell Death
Exp.
Doxorubicin (0.014 uM)
AZD7648 (1.111 uM)
combined
Synergy(ZIP)
%Cell Death

j

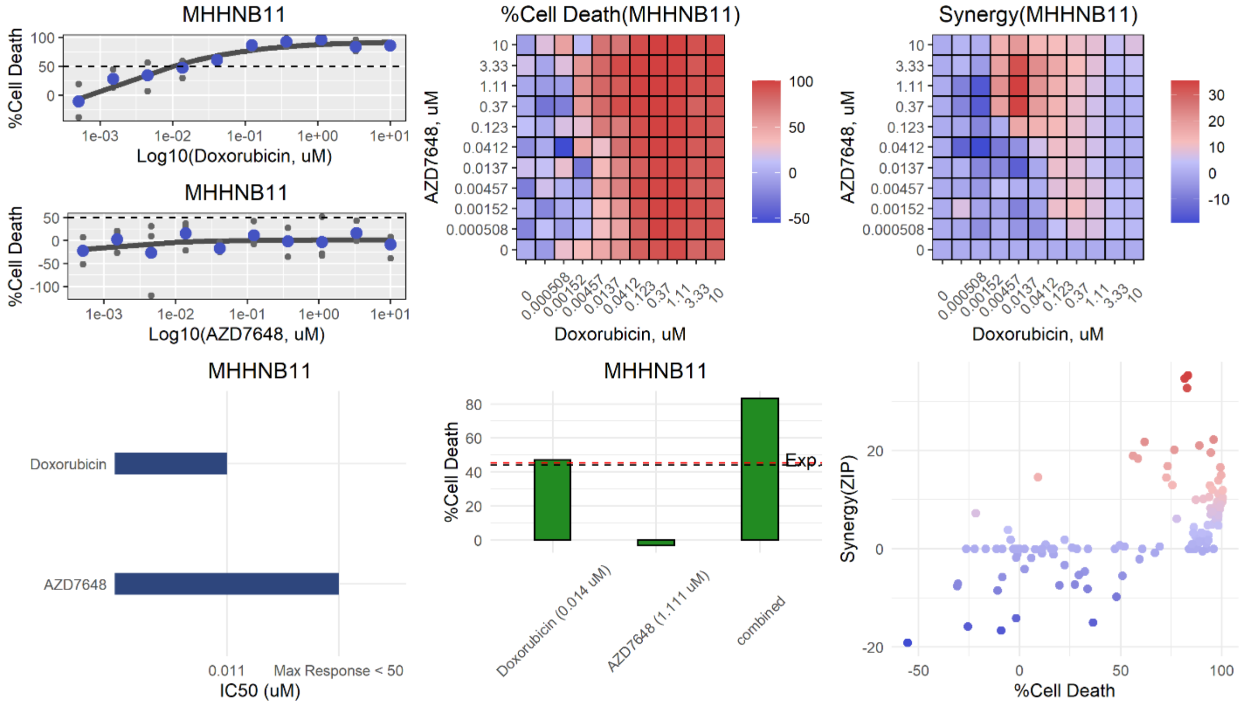
MHHNB11
%Cell Death
Log10(Doxorubicin, uM)
MHHNB11
%Cell Death
Log10(AZD7648, uM)
%Cell Death(MHHNB11)
AZD7648, uM
Doxorubicin, uM
Synergy(MHHNB11)
AZD7648, uM
Doxorubicin, uM
MHHNB11
Doxorubicin
AZD7648
0.011
Max Response < 50
IC50 (uM)
MHHNB11
%Cell Death
Exp.
Doxorubicin (0.014 uM)
AZD7648 (1.111 uM)
combined
Synergy(ZIP)
%Cell Death

k

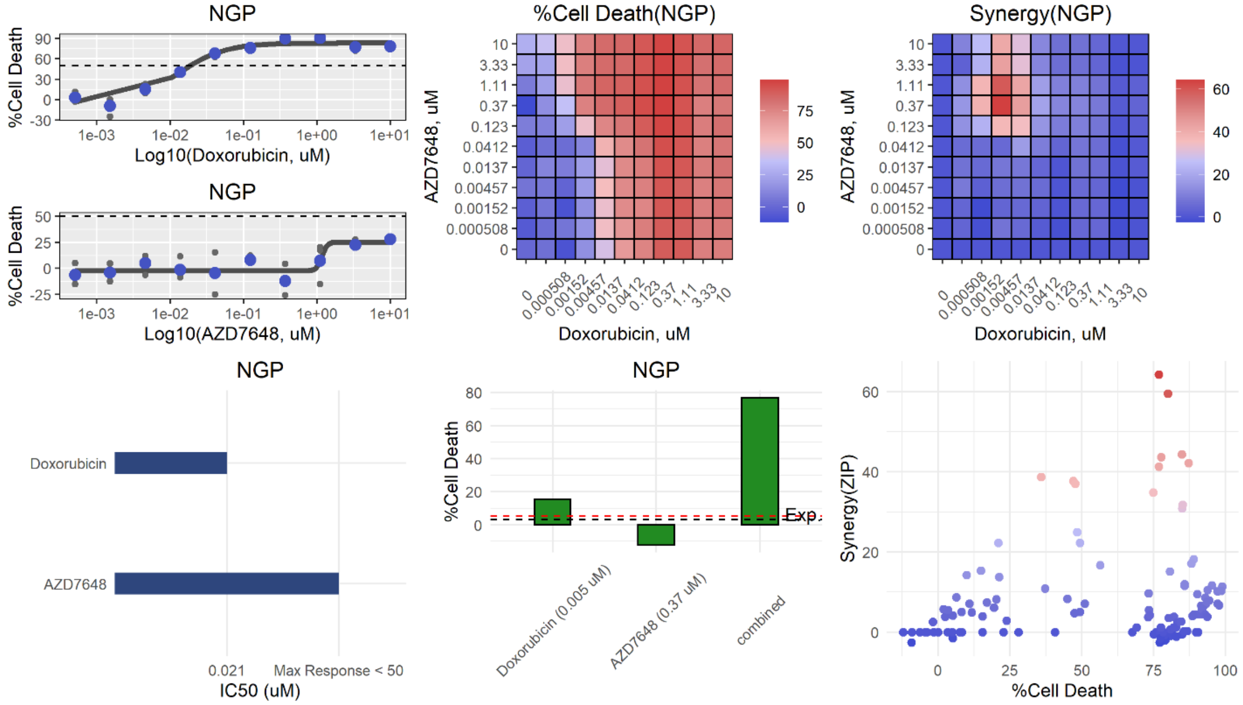
NGP
%Cell Death
Log10(Doxorubicin, uM)
Log10(AZD7648, uM)
%Cell Death(NGP)
AZD7648, uM
Doxorubicin, uM
Synergy(NGP)
IC50 (uM)
Doxorubicin
AZD7648
0.021
Max Response < 50
Doxorubicin (0.005 uM)
AZD7648 (0.37 uM)
combined
Exp.
Synergy(ZIP)

l

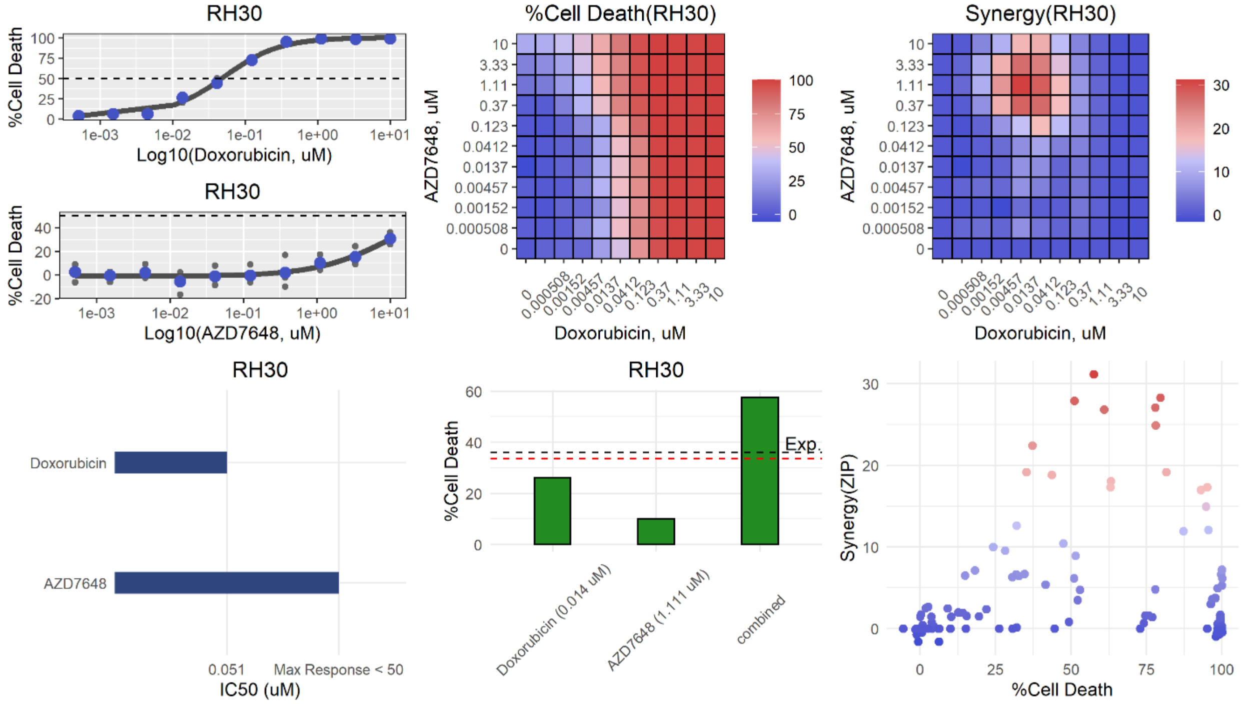
RH30
%Cell Death
Log10(Doxorubicin, uM)
Log10(AZD7648, uM)
%Cell Death(RH30)
AZD7648, uM
Doxorubicin, uM
Synergy(RH30)
IC50 (uM)
Doxorubicin
AZD7648
0.051
Max Response < 50
Doxorubicin (0.014 uM)
AZD7648 (1.111 uM)
combined
Exp.
Synergy(ZIP)

m

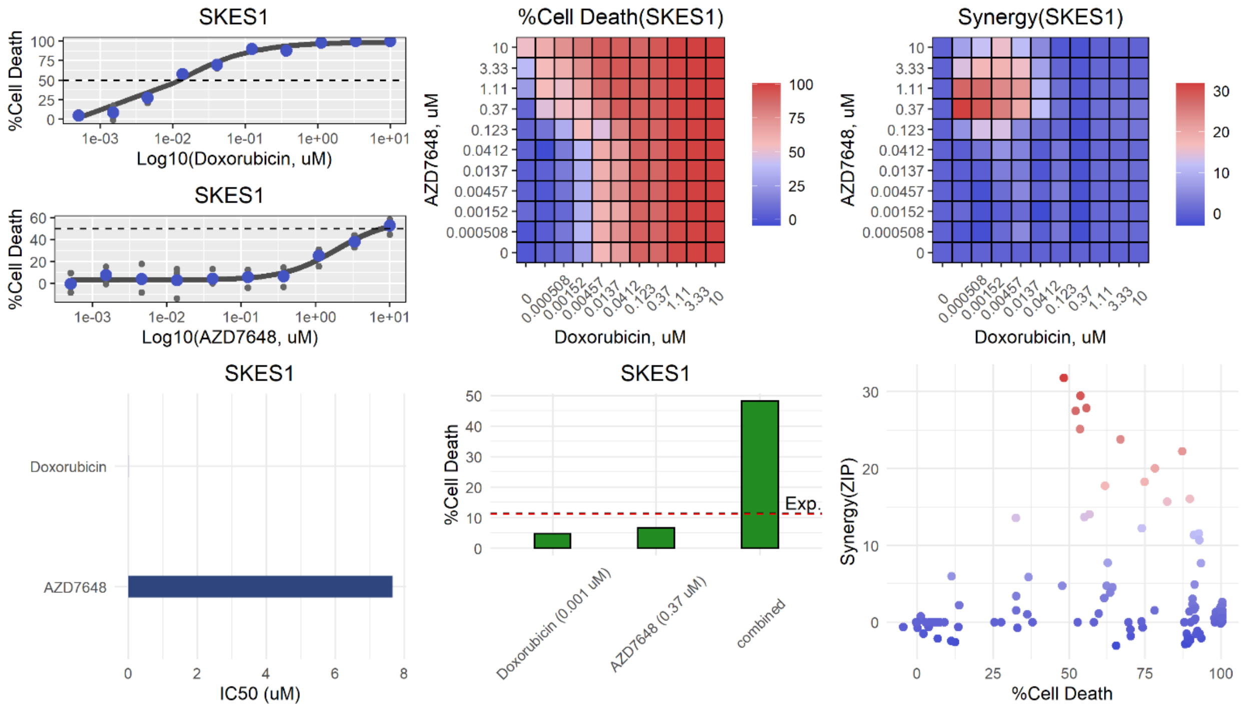
SKES1
%Cell Death
Log10(Doxorubicin, uM)
SKES1
%Cell Death
Log10(AZD7648, uM)
%Cell Death(SKES1)
AZD7648, uM
Doxorubicin, uM
Synergy(SKES1)
AZD7648, uM
Doxorubicin, uM
SKES1
Doxorubicin
AZD7648
IC50 (uM)
SKES1
%Cell Death
Exp.
Doxorubicin (0.001 uM)
AZD7648 (0.37 uM)
combined
Synergy(ZIP)
%Cell Death

n

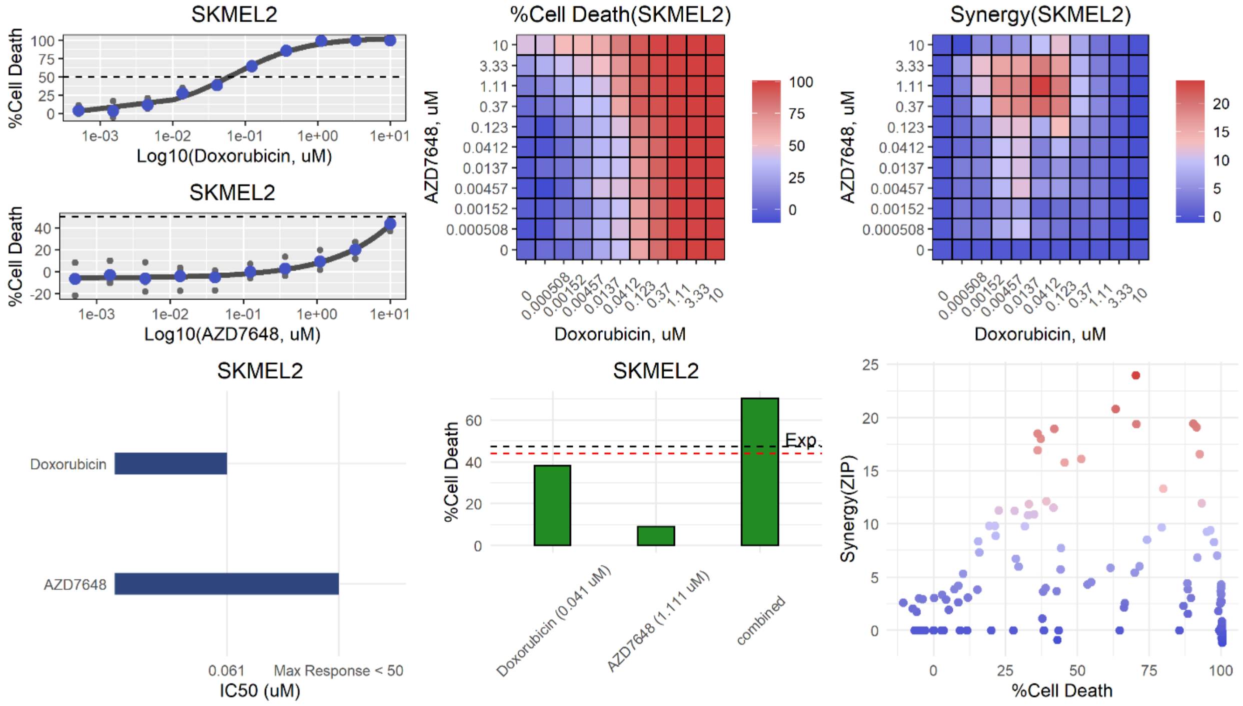
SKMEL2
%Cell Death
Log10(Doxorubicin, uM)
SKMEL2
%Cell Death
Log10(AZD7648, uM)
%Cell Death(SKMEL2)
AZD7648, uM
Doxorubicin, uM
Synergy(SKMEL2)
AZD7648, uM
Doxorubicin, uM
SKMEL2
Doxorubicin
AZD7648
0.061
Max Response < 50
IC50 (uM)
SKMEL2
%Cell Death
Exp.
Doxorubicin (0.041 uM)
AZD7648 (1.111 uM)
combined
Synergy(ZIP)
%Cell Death

o

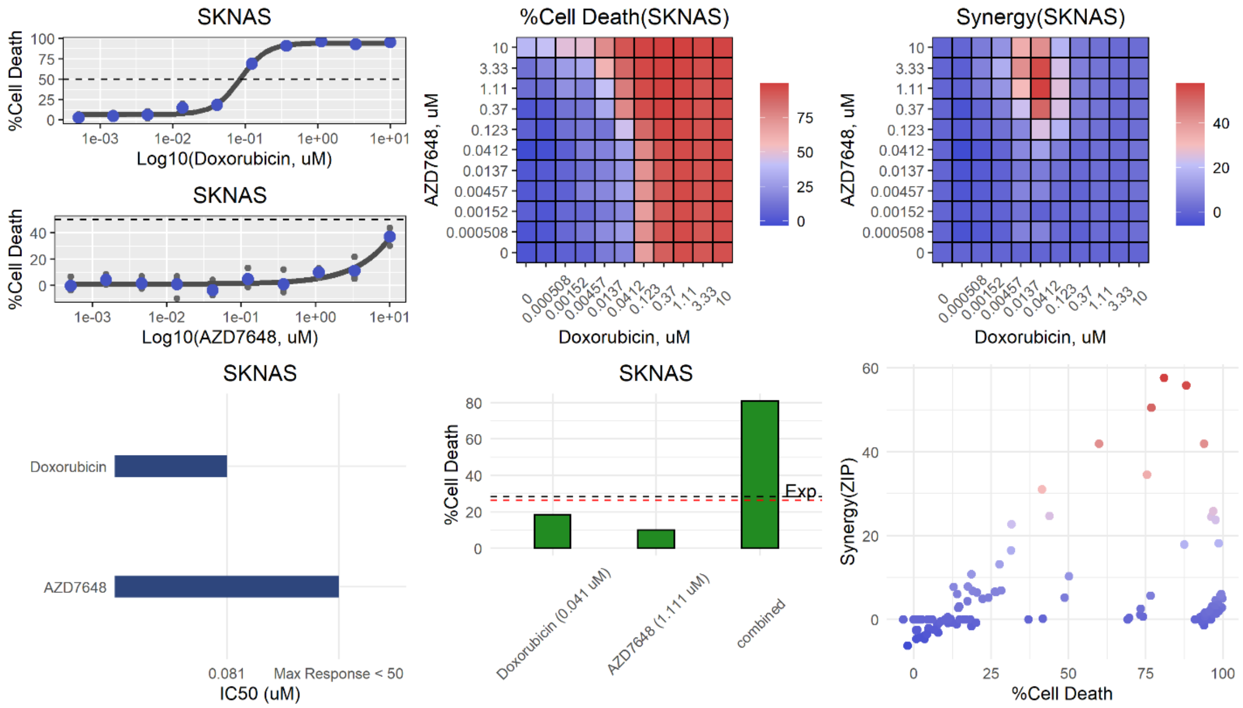
SKNAS
%Cell Death
Log10(Doxorubicin, uM)
SKNAS
%Cell Death
Log10(AZD7648, uM)
%Cell Death(SKNAS)
AZD7648, uM
Doxorubicin, uM
Synergy(SKNAS)
AZD7648, uM
Doxorubicin, uM
SKNAS
Doxorubicin
AZD7648
0.081
Max Response < 50
IC50 (uM)
SKNAS
%Cell Death
Exp.
Doxorubicin (0.041 uM)
AZD7648 (1.111 uM)
combined
Synergy(ZIP)
%Cell Death

p

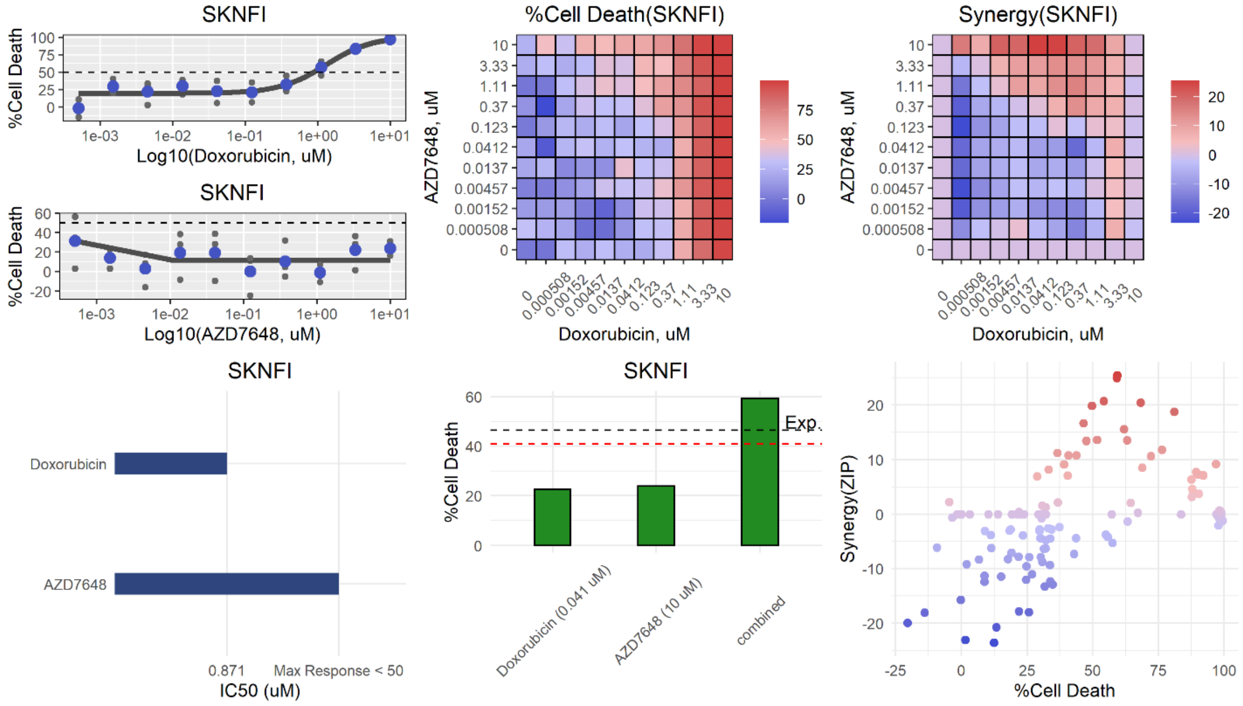
SKNFI
%Cell Death
Log10(Doxorubicin, uM)
SKNFI
%Cell Death
Log10(AZD7648, uM)
%Cell Death(SKNFI)
AZD7648, uM
Doxorubicin, uM
Synergy(SKNFI)
AZD7648, uM
Doxorubicin, uM
SKNFI
Doxorubicin
AZD7648
0.871
Max Response < 50
IC50 (uM)
SKNFI
%Cell Death
Exp.
Doxorubicin (0.041 uM)
AZD7648 (10 uM)
combined
Synergy(ZIP)
%Cell Death

q

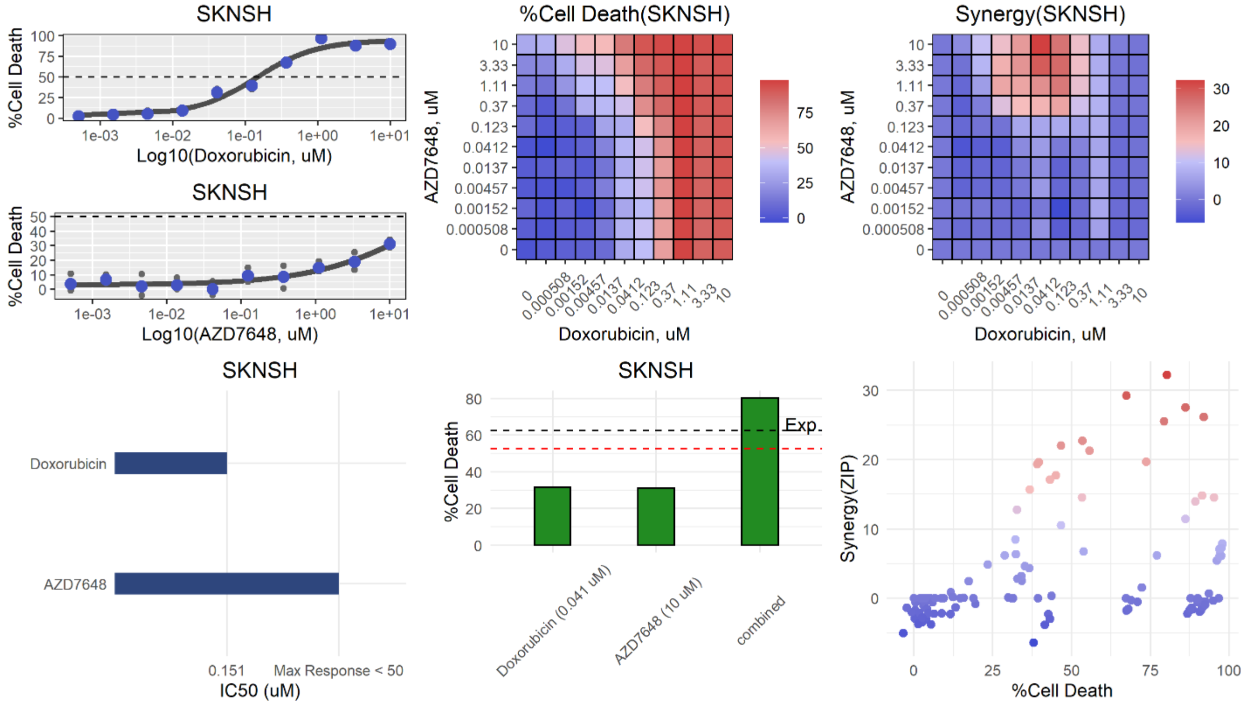
SKNSH
%Cell Death
Log10(Doxorubicin, uM)
SKNSH
%Cell Death
Log10(AZD7648, uM)
%Cell Death(SKNSH)
AZD7648, uM
Doxorubicin, uM
Synergy(SKNSH)
AZD7648, uM
Doxorubicin, uM
SKNSH
Doxorubicin
AZD7648
0.151
Max Response < 50
IC50 (uM)
SKNSH
%Cell Death
Exp.
Doxorubicin (0.041 uM)
AZD7648 (10 uM)
combined
Synergy(ZIP)
%Cell Death

r

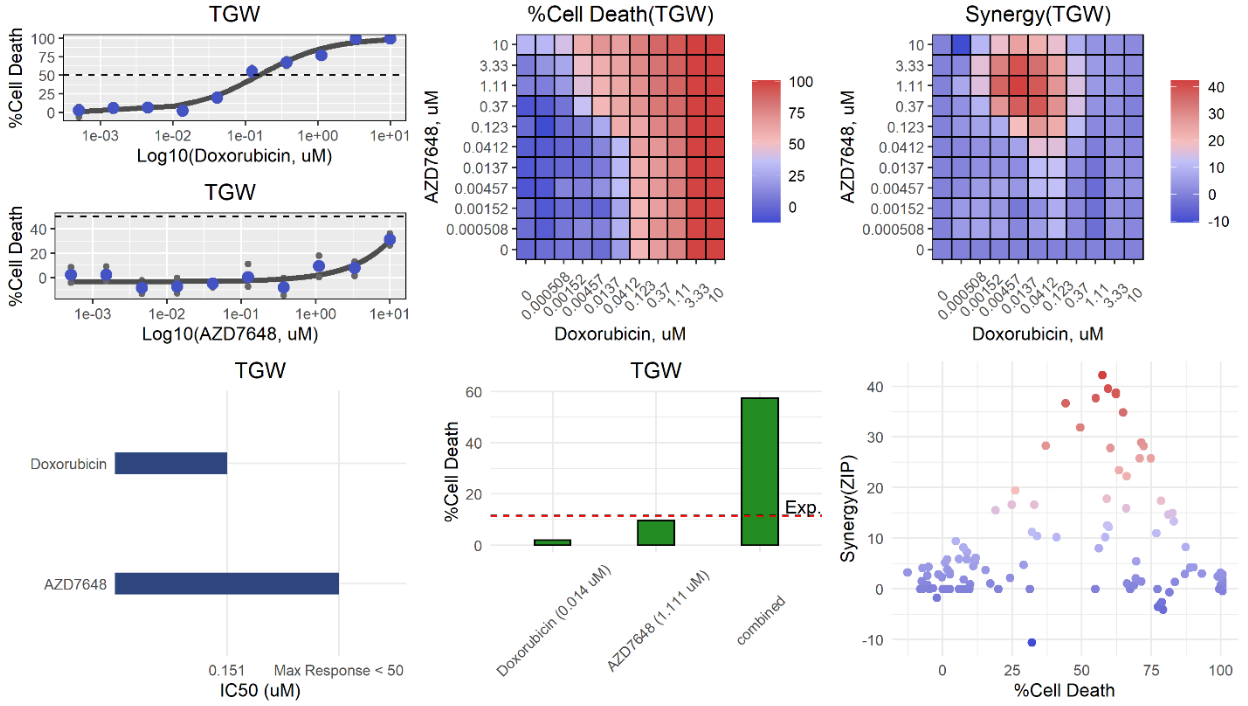
TGW
%Cell Death
Log10(Doxorubicin, uM)
TGW
%Cell Death
Log10(AZD7648, uM)
%Cell Death(TGW)
AZD7648, uM
Doxorubicin, uM
Synergy(TGW)
AZD7648, uM
Doxorubicin, uM
TGW
Doxorubicin
AZD7648
0.151
Max Response < 50
IC50 (uM)
TGW
%Cell Death
Exp.
Doxorubicin (0.014 uM)
AZD7648 (1.111 uM)
combined
Synergy(ZIP)
%Cell Death

Supplementary Figure 6. Summary of dense doxorubicin-AZD7648 combinations in all cell lines.

(a - r) 18 panels for combination of doxorubicin and AZD7648 in all 18 cell lines. a) AC16, b) BE2C, c) BJ-TERT, d) CHP212, e) GIMEN, f) GM12878, g) HCT116, h) HKE293T, i) KELLY, j) MHHNB11, k) NGP, l) RH30, m) SKES1, n) SKMEL2, o) SKNAS, p) SKNFI, q) SKNSH, r) TGW. Dose-response curves for each single-agent (top left), normalized cell death matrix (top middle), synergy matrix (top right; Note: units are δZIP), IC_{50} values of each single-agent (lower left), barplots corresponding to maximum synergy (lower middle; Note: dashed lines indicate expected values under additivity (black) or Bliss independence (red)), and scatterplot of cell death vs. synergy (lower right) for all 18 cell lines used in dense drug-drug screens.

a

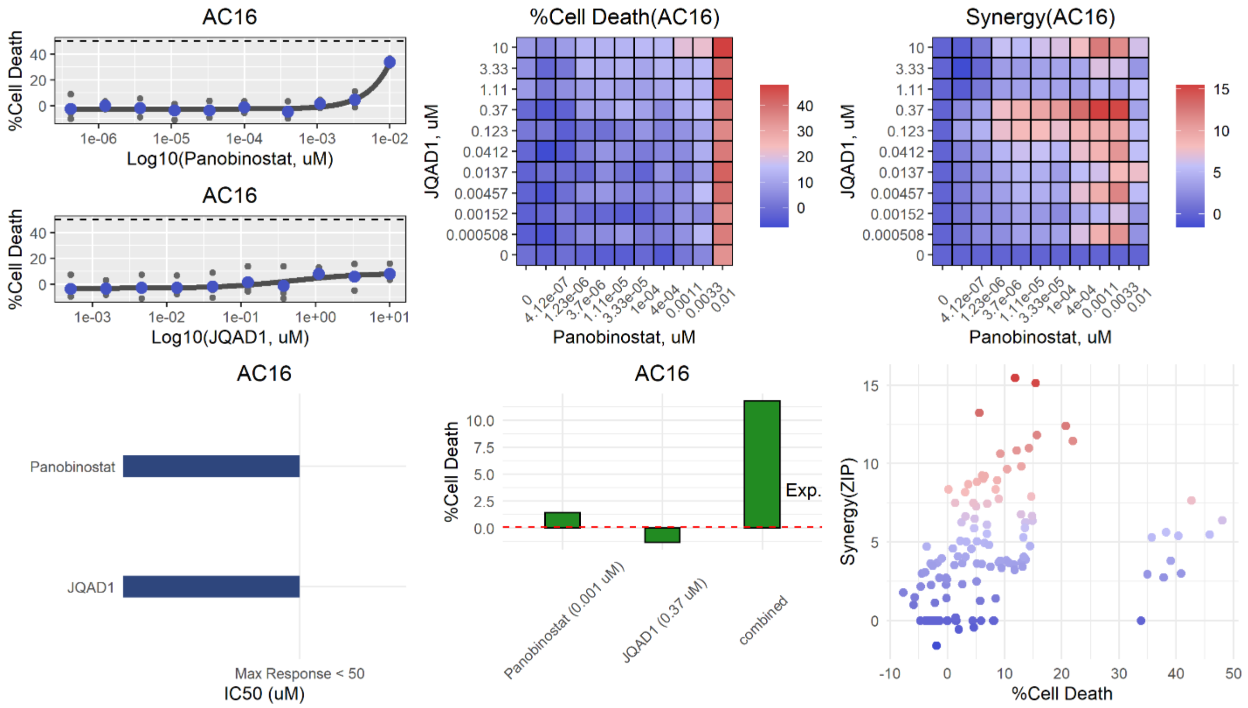
AC16
%Cell Death
Log10(Panobinostat, uM)
1e-06
1e-05
1e-04
1e-03
1e-02
AC16
Log10(JQAD1, uM)
1e-03
1e-02
1e-01
1e+00
1e+01
%Cell Death(AC16)
JQAD1, uM
10
3.33
1.11
0.37
0.123
0.0412
0.0137
0.00457
0.00152
0.000508
0
Panobinostat, uM
0
4.12e-07
1.23e-06
3.7e-06
1.11e-05
3.33e-05
1e-04
4e-04
0.0011
0.0033
0.01
Synergy(AC16)
AC16
Panobinostat
JQAD1
Max Response < 50
IC50 (uM)
AC16
%Cell Death
Exp.
Panobinostat (0.001 uM)
JQAD1 (0.37 uM)
combined
Synergy(ZIP)
%Cell Death

b

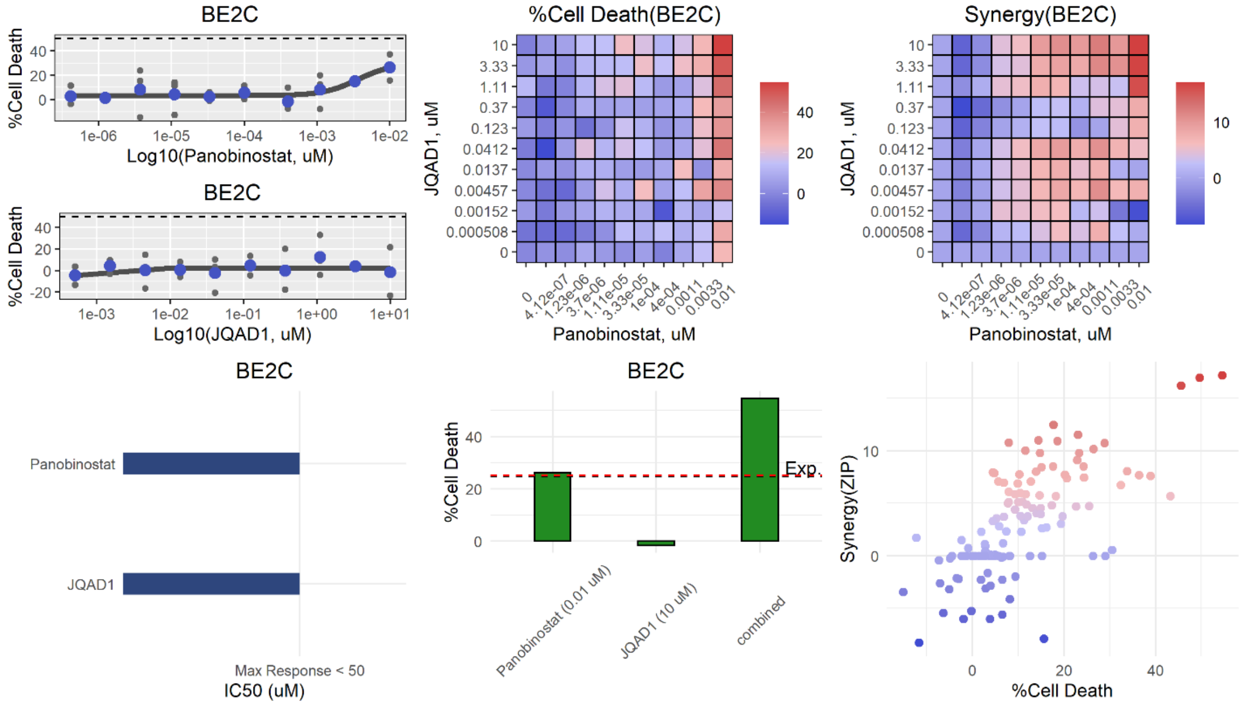
BE2C
%Cell Death
Log10(Panobinostat, uM)
1e-06
1e-05
1e-04
1e-03
1e-02
BE2C
Log10(JQAD1, uM)
1e-03
1e-02
1e-01
1e+00
1e+01
%Cell Death(BE2C)
JQAD1, uM
10
3.33
1.11
0.37
0.123
0.0412
0.0137
0.00457
0.00152
0.000508
0
Panobinostat, uM
0
4.12e-07
1.23e-06
3.7e-06
1.11e-05
3.33e-05
1e-04
4e-04
0.0011
0.0033
0.01
Synergy(BE2C)
BE2C
Panobinostat
JQAD1
Max Response < 50
IC50 (uM)
BE2C
%Cell Death
Exp.
Panobinostat (0.01 uM)
JQAD1 (10 uM)
combined
Synergy(ZIP)
%Cell Death

c

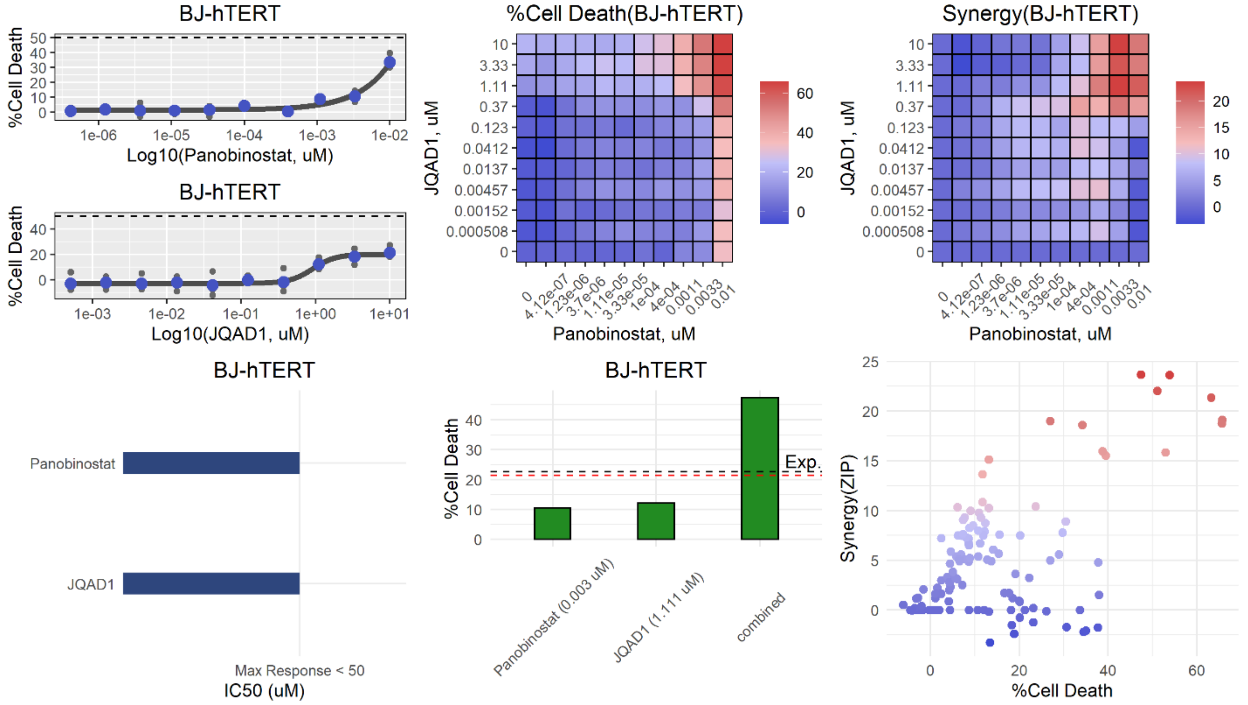
BJ-hTERT
%Cell Death
Log10(Panobinostat, uM)
BJ-hTERT
%Cell Death
Log10(JQAD1, uM)
%Cell Death(BJ-hTERT)
JQAD1, uM
Panobinostat, uM
Synergy(BJ-hTERT)
JQAD1, uM
Panobinostat, uM
BJ-hTERT
Panobinostat
JQAD1
Max Response < 50
IC50 (uM)
BJ-hTERT
%Cell Death
Exp.
Panobinostat (0.003 uM)
JQAD1 (1.111 uM)
combined
Synergy(ZIP)
%Cell Death

d

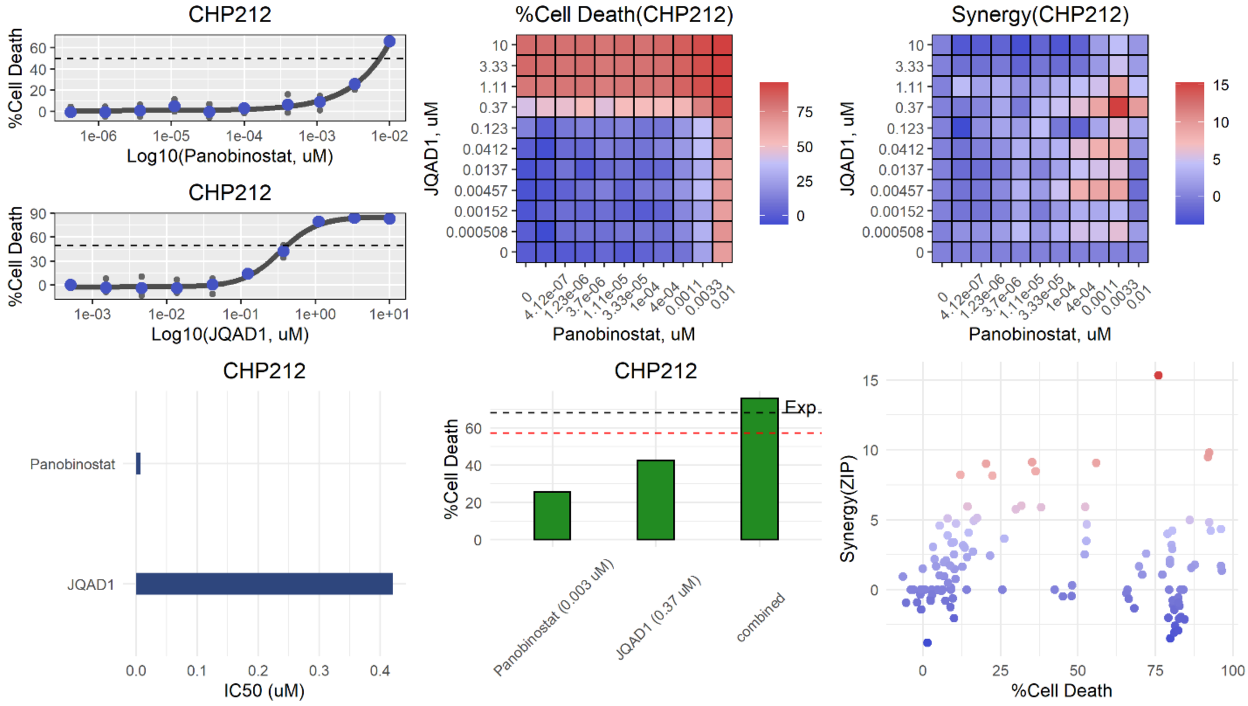
CHP212
%Cell Death
Log10(Panobinostat, uM)
CHP212
%Cell Death
Log10(JQAD1, uM)
%Cell Death(CHP212)
JQAD1, uM
Panobinostat, uM
Synergy(CHP212)
JQAD1, uM
Panobinostat, uM
CHP212
Panobinostat
JQAD1
IC50 (uM)
CHP212
%Cell Death
Exp.
Panobinostat (0.003 uM)
JQAD1 (0.37 uM)
combined
Synergy(ZIP)
%Cell Death

e

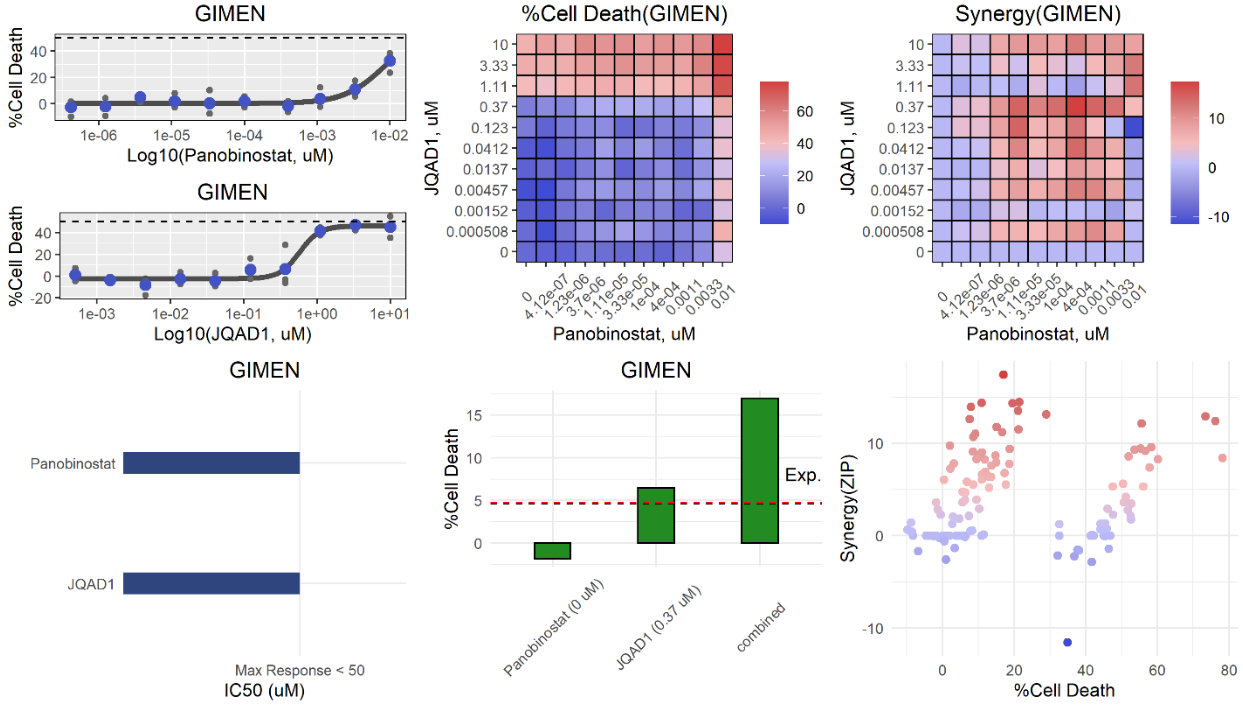

GIMEN
%Cell Death
Log10(Panobinostat, uM)
1e-06
1e-05
1e-04
1e-03
1e-02
GIMEN
Log10(JQAD1, uM)
1e-03
1e-02
1e-01
1e+00
1e+01
%Cell Death(GIMEN)
JQAD1, uM
Panobinostat, uM
Synergy(GIMEN)
GIMEN
Panobinostat
JQAD1
Max Response < 50
IC50 (uM)
GIMEN
%Cell Death
Panobinostat (0 uM)
JQAD1 (0.37 uM)
combined
Exp.
Synergy(ZIP)
%Cell Death

f

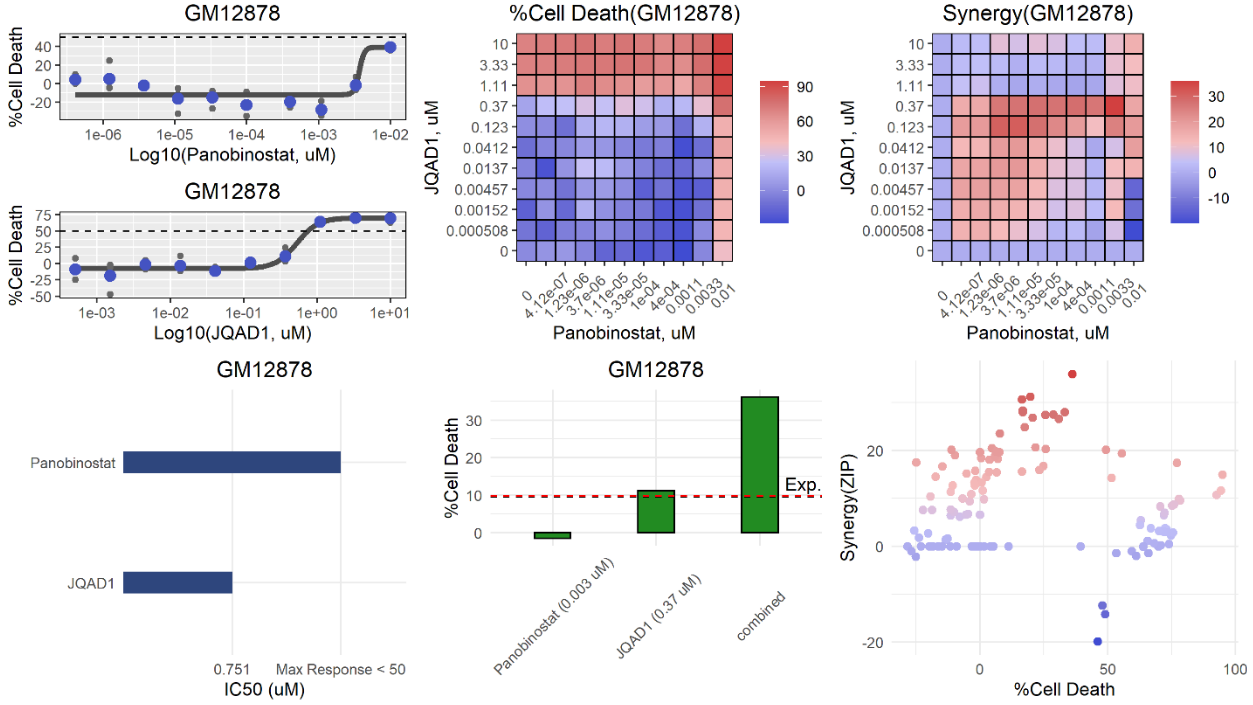

GM12878
%Cell Death
Log10(Panobinostat, uM)
1e-06
1e-05
1e-04
1e-03
1e-02
GM12878
Log10(JQAD1, uM)
1e-03
1e-02
1e-01
1e+00
1e+01
%Cell Death(GM12878)
JQAD1, uM
Panobinostat, uM
Synergy(GM12878)
GM12878
Panobinostat
JQAD1
0.751
Max Response < 50
IC50 (uM)
GM12878
%Cell Death
Panobinostat (0.003 uM)
JQAD1 (0.37 uM)
combined
Exp.
Synergy(ZIP)
%Cell Death

g

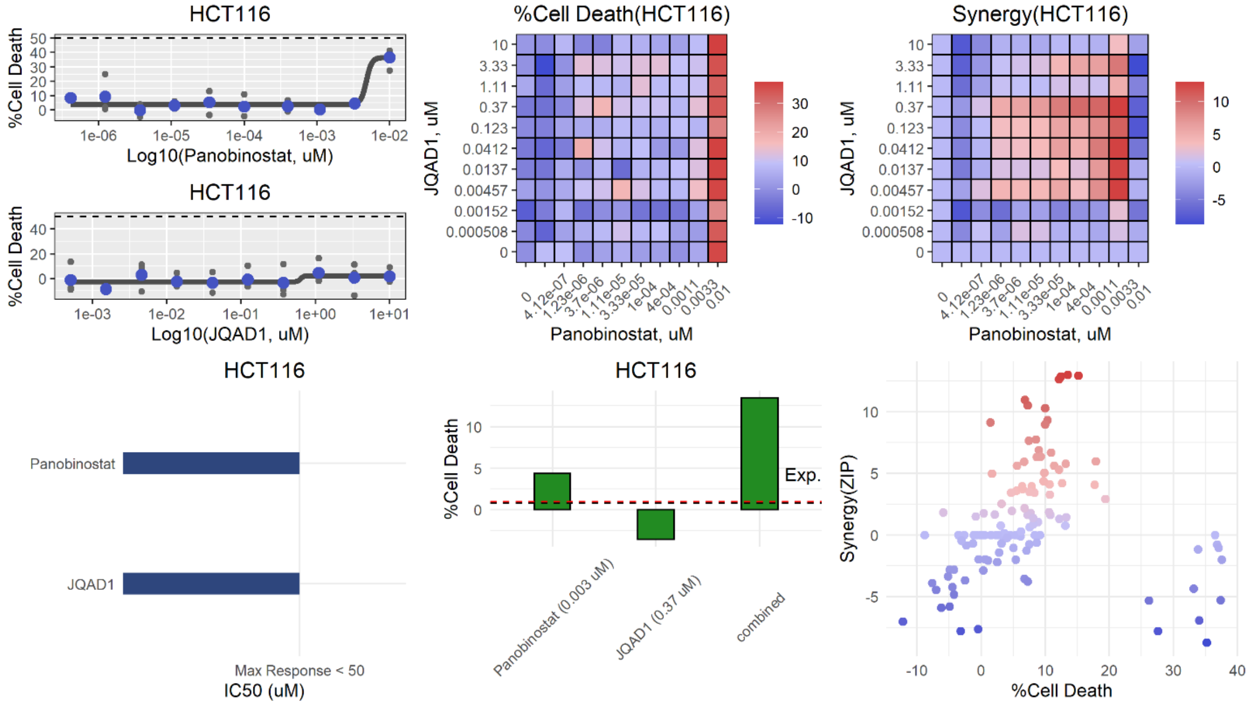
HCT116
%Cell Death
Log10(Panobinostat, uM)
HCT116
Log10(JQAD1, uM)
%Cell Death(HCT116)
JQAD1, uM
Panobinostat, uM
Synergy(HCT116)
HCT116
Panobinostat
JQAD1
Max Response < 50
IC50 (uM)
HCT116
Exp.
Panobinostat (0.003 uM)
JQAD1 (0.37 uM)
combined
Synergy(ZIP)
%Cell Death

h

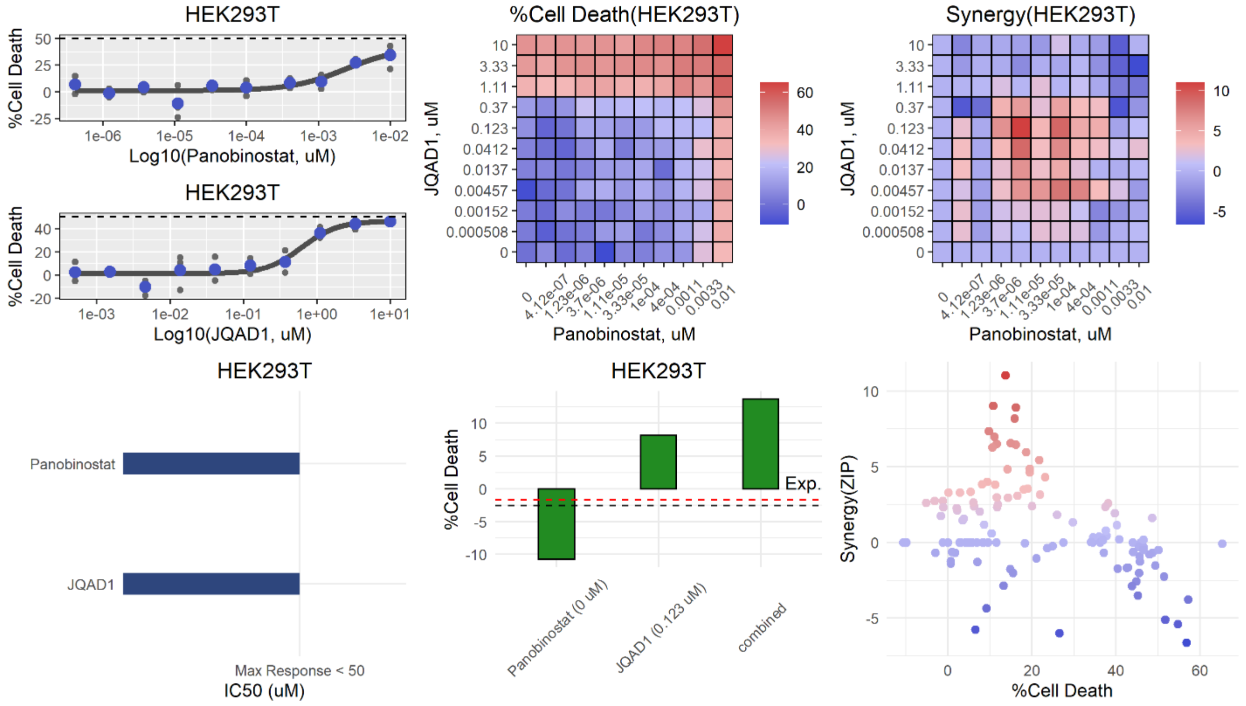
HEK293T
%Cell Death
Log10(Panobinostat, uM)
HEK293T
Log10(JQAD1, uM)
%Cell Death(HEK293T)
JQAD1, uM
Panobinostat, uM
Synergy(HEK293T)
HEK293T
Panobinostat
JQAD1
Max Response < 50
IC50 (uM)
HEK293T
Exp.
Panobinostat (0 uM)
JQAD1 (0.123 uM)
combined
Synergy(ZIP)
%Cell Death

i

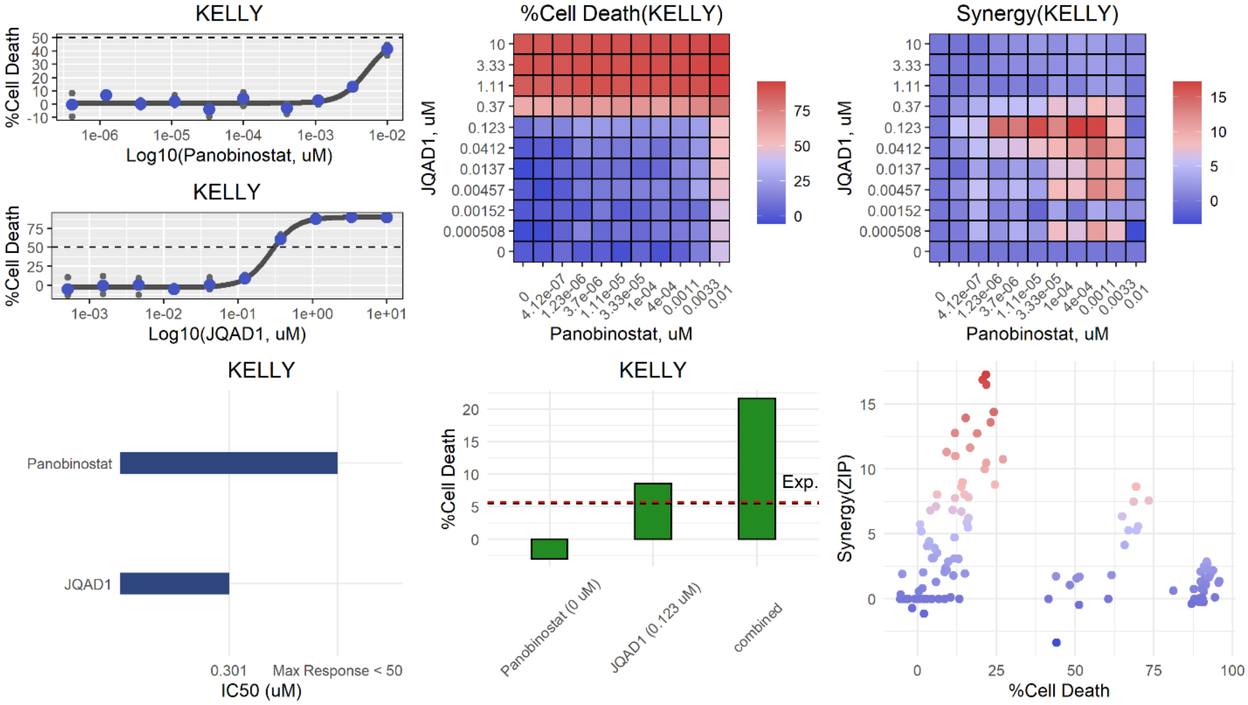

KELLY
%Cell Death
Log10(Panobinostat, uM)
Log10(JQAD1, uM)
%Cell Death(KELLY)
Synergy(KELLY)
JQAD1, uM
Panobinostat, uM
Panobinostat
JQAD1
0.301
Max Response < 50
IC50 (uM)
Panobinostat (0 uM)
JQAD1 (0.123 uM)
combined
Exp.
Synergy(ZIP)

j

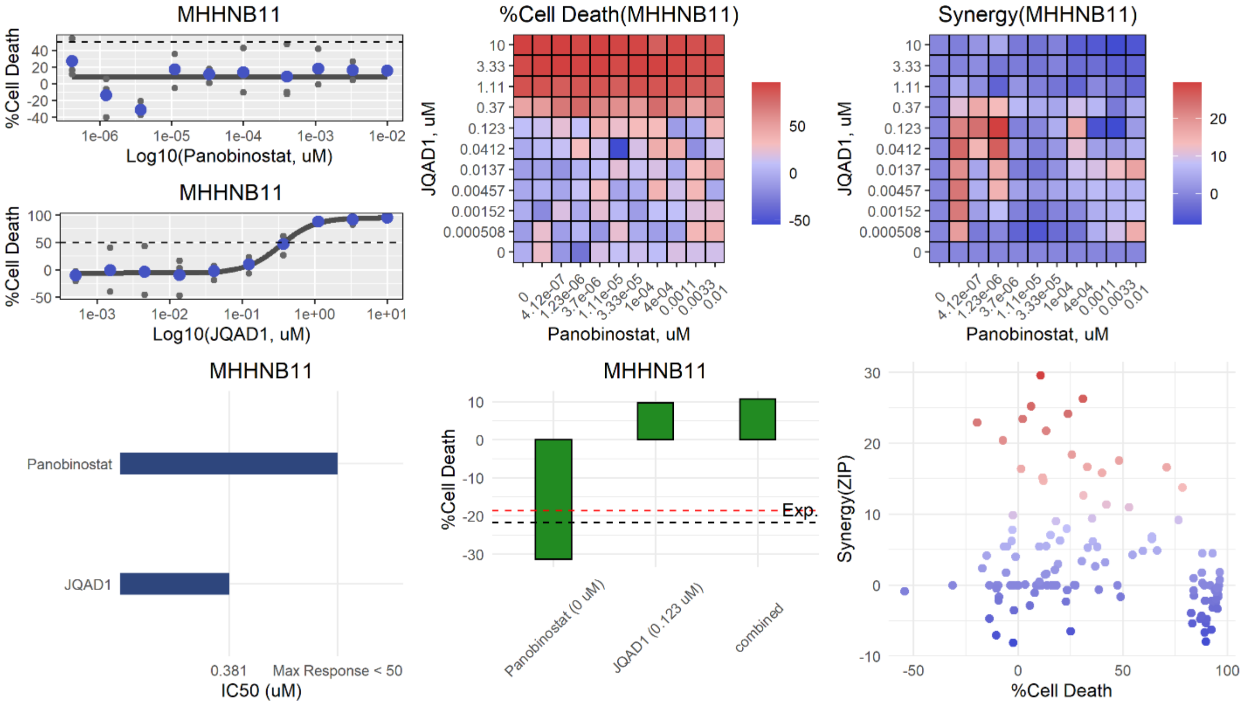

MHHNB11
%Cell Death
Log10(Panobinostat, uM)
Log10(JQAD1, uM)
%Cell Death(MHHNB11)
Synergy(MHHNB11)
JQAD1, uM
Panobinostat, uM
Panobinostat
JQAD1
0.381
Max Response < 50
IC50 (uM)
Panobinostat (0 uM)
JQAD1 (0.123 uM)
combined
Exp.
Synergy(ZIP)

k

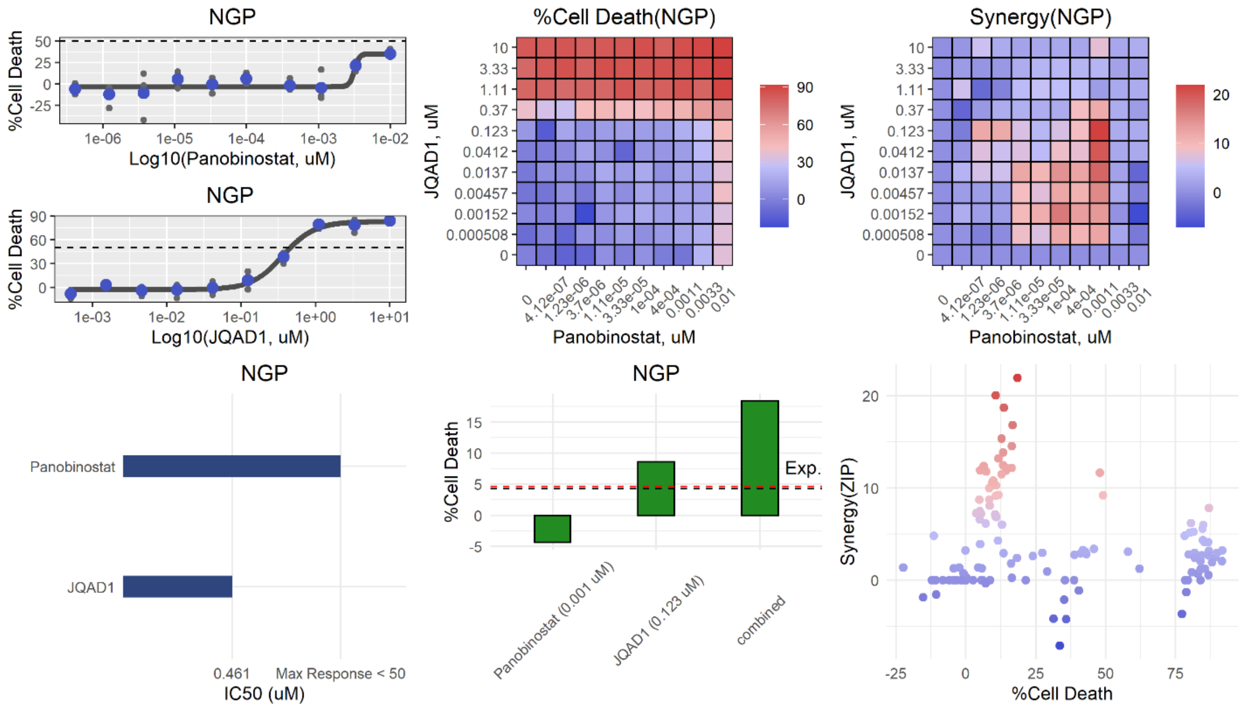
NGP
%Cell Death
Log10(Panobinostat, uM)
NGP
%Cell Death
Log10(JQAD1, uM)
%Cell Death(NGP)
JQAD1, uM
Panobinostat, uM
Synergy(NGP)
JQAD1, uM
Panobinostat, uM
NGP
Panobinostat
JQAD1
0.461
Max Response < 50
IC50 (uM)
NGP
%Cell Death
Exp.
Panobinostat (0.001 uM)
JQAD1 (0.123 uM)
combined
Synergy(ZIP)
%Cell Death

l

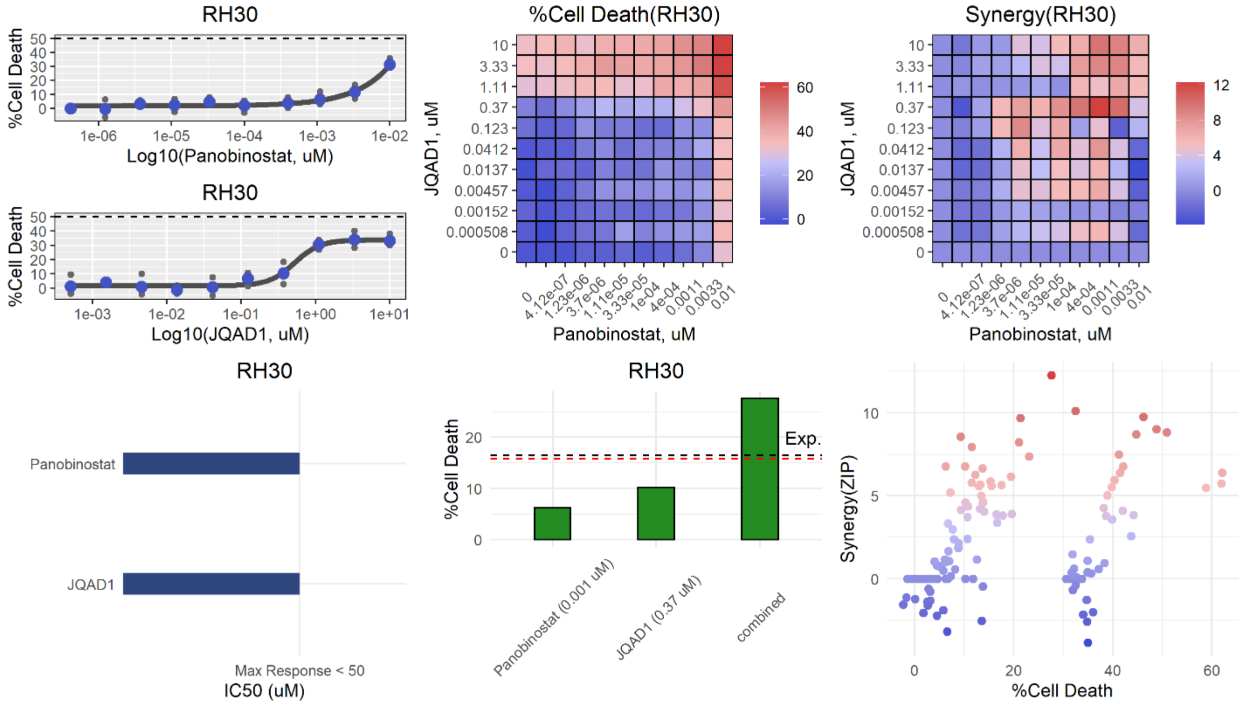
RH30
%Cell Death
Log10(Panobinostat, uM)
RH30
%Cell Death
Log10(JQAD1, uM)
%Cell Death(RH30)
JQAD1, uM
Panobinostat, uM
Synergy(RH30)
JQAD1, uM
Panobinostat, uM
RH30
Panobinostat
JQAD1
Max Response < 50
IC50 (uM)
RH30
%Cell Death
Exp.
Panobinostat (0.001 uM)
JQAD1 (0.37 uM)
combined
Synergy(ZIP)
%Cell Death

m

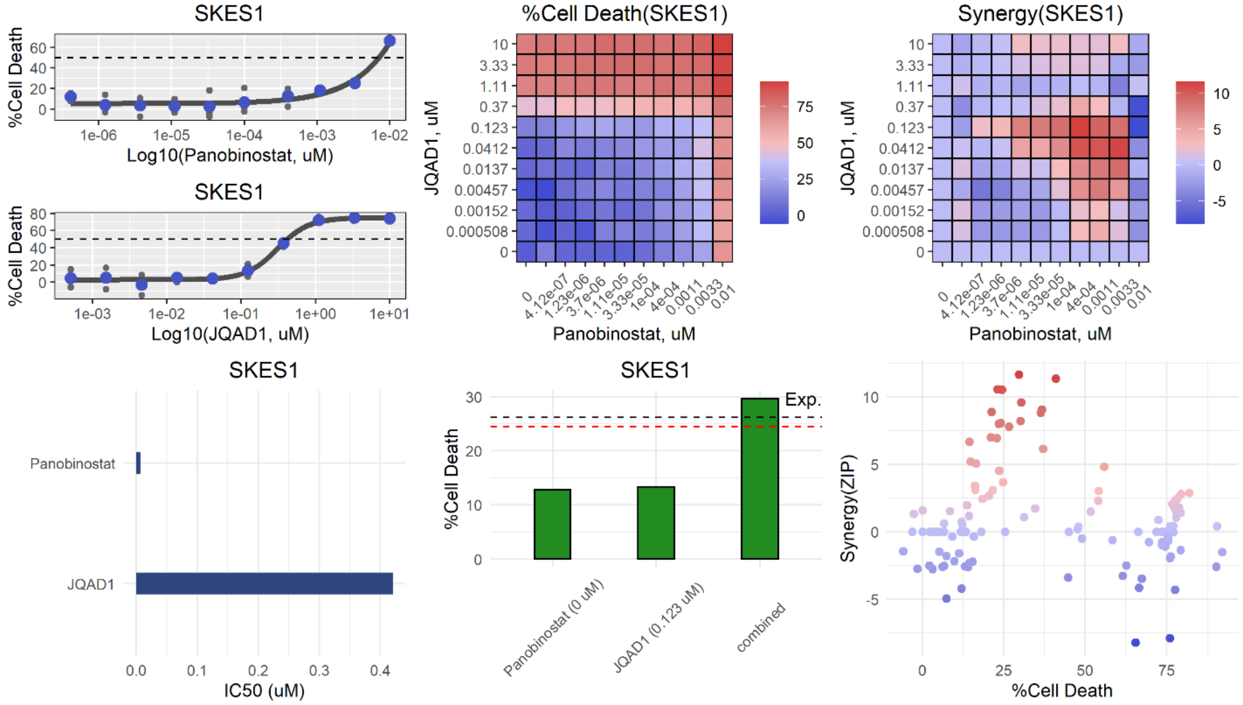
SKES1
%Cell Death
Log10(Panobinostat, uM)
Log10(JQAD1, uM)
%Cell Death(SKES1)
Synergy(SKES1)
JQAD1, uM
Panobinostat, uM
Panobinostat
JQAD1
IC50 (uM)
Exp.
Panobinostat (0 uM)
JQAD1 (0.123 uM)
combined
Synergy(ZIP)

n

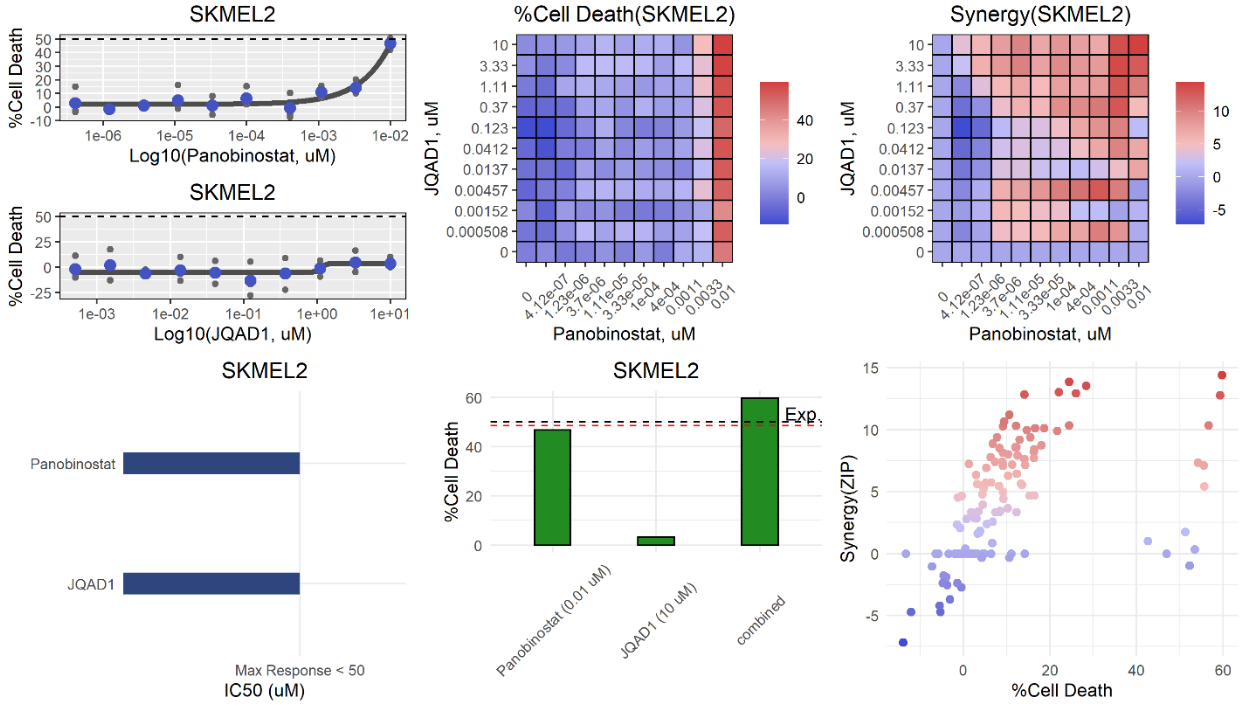
SKMEL2
%Cell Death
Log10(Panobinostat, uM)
Log10(JQAD1, uM)
%Cell Death(SKMEL2)
Synergy(SKMEL2)
JQAD1, uM
Panobinostat, uM
Panobinostat
JQAD1
Max Response < 50
IC50 (uM)
Exp.
Panobinostat (0.01 uM)
JQAD1 (10 uM)
combined
Synergy(ZIP)

o

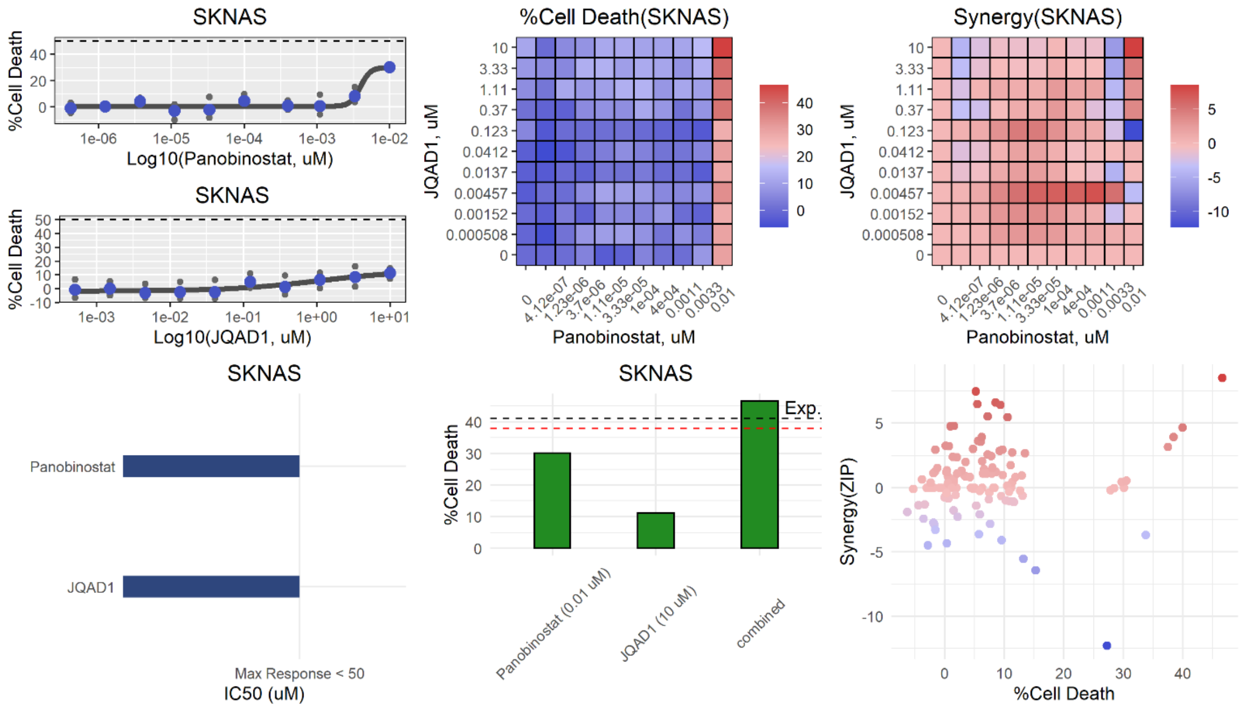
SKNAS
%Cell Death
Log10(Panobinostat, uM)
SKNAS
%Cell Death
Log10(JQAD1, uM)
%Cell Death(SKNAS)
JQAD1, uM
Panobinostat, uM
Synergy(SKNAS)
JQAD1, uM
Panobinostat, uM
SKNAS
Panobinostat
JQAD1
Max Response < 50
IC50 (uM)
SKNAS
%Cell Death
Exp.
Panobinostat (0.01 uM)
JQAD1 (10 uM)
combined
Synergy(ZIP)
%Cell Death

p

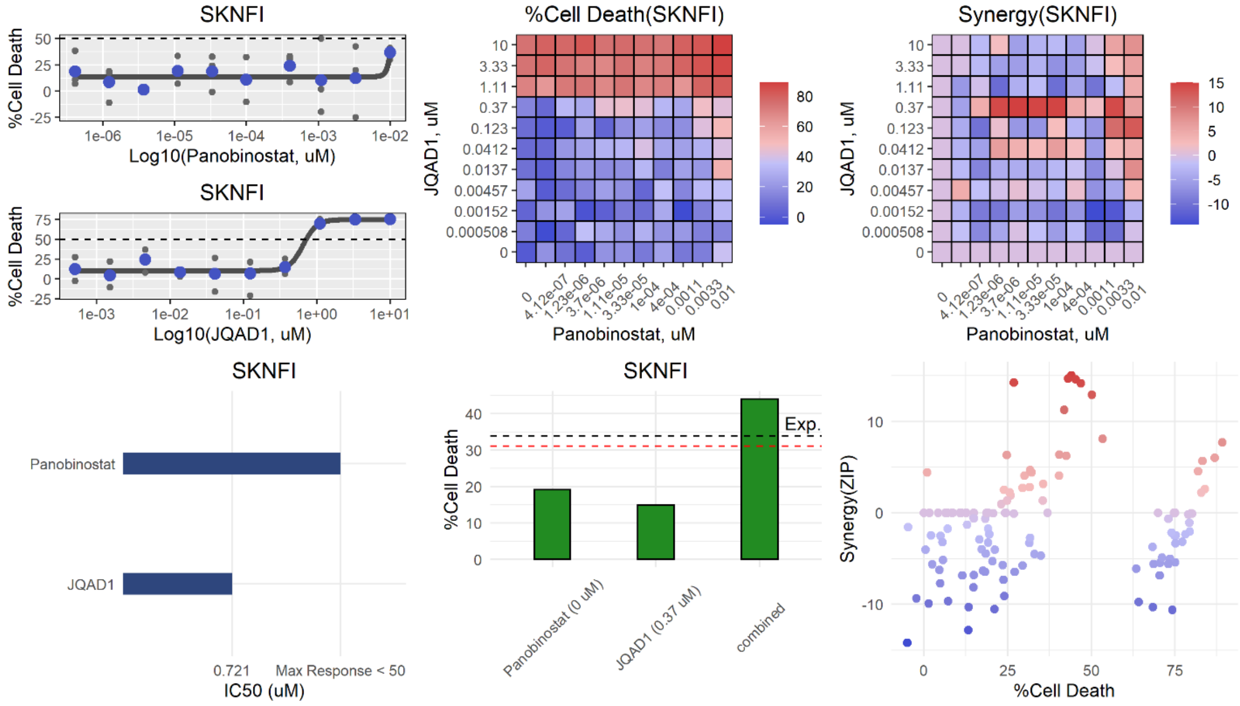
SKNFI
%Cell Death
Log10(Panobinostat, uM)
SKNFI
%Cell Death
Log10(JQAD1, uM)
%Cell Death(SKNFI)
JQAD1, uM
Panobinostat, uM
Synergy(SKNFI)
JQAD1, uM
Panobinostat, uM
SKNFI
Panobinostat
JQAD1
0.721
Max Response < 50
IC50 (uM)
SKNFI
%Cell Death
Exp.
Panobinostat (0 uM)
JQAD1 (0.37 uM)
combined
Synergy(ZIP)
%Cell Death

q

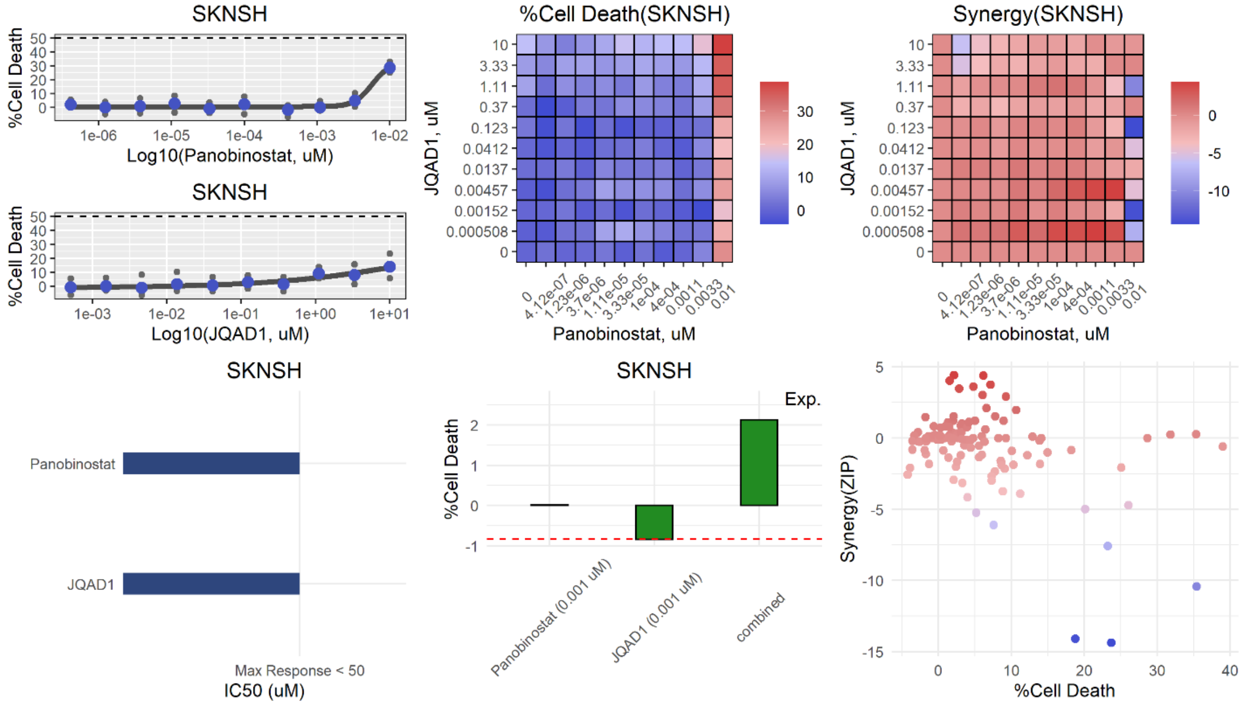
SKNSH
%Cell Death
Log10(Panobinostat, uM)
SKNSH
%Cell Death
Log10(JQAD1, uM)
%Cell Death(SKNSH)
JQAD1, uM
Panobinostat, uM
Synergy(SKNSH)
JQAD1, uM
Panobinostat, uM
SKNSH
Panobinostat
JQAD1
Max Response < 50
IC50 (uM)
SKNSH
%Cell Death
Exp.
Panobinostat (0.001 uM)
JQAD1 (0.001 uM)
combined
Synergy(ZIP)
%Cell Death

r

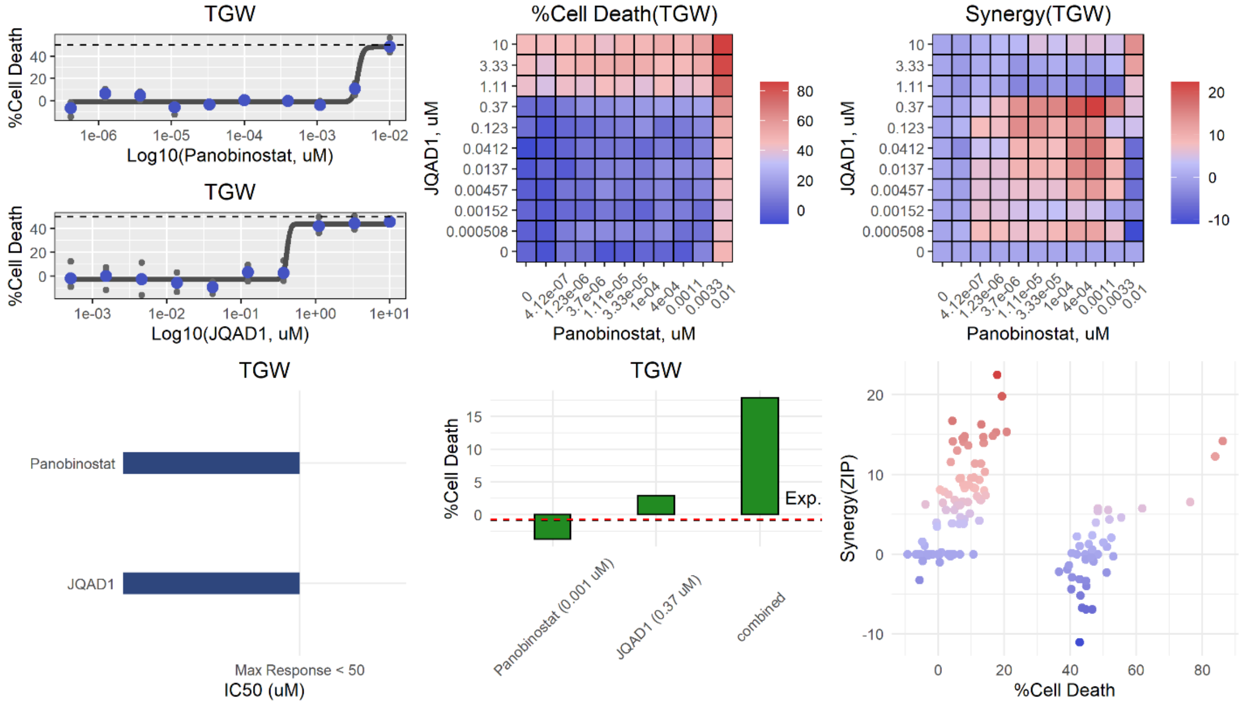
TGW
%Cell Death
Log10(Panobinostat, uM)
TGW
%Cell Death
Log10(JQAD1, uM)
%Cell Death(TGW)
JQAD1, uM
Panobinostat, uM
Synergy(TGW)
JQAD1, uM
Panobinostat, uM
TGW
Panobinostat
JQAD1
Max Response < 50
IC50 (uM)
TGW
%Cell Death
Exp.
Panobinostat (0.001 uM)
JQAD1 (0.37 uM)
combined
Synergy(ZIP)
%Cell Death

Supplementary Figure 7. Summary of dense JQAD1-panobinostat combinations in all cell lines.

(a - r) 18 panels for combination of JQAD1 and panobinostat in all 18 cell lines. a) AC16, b) BE2C, c) BJ-TERT, d) CHP212, e) GIMEN, f) GM12878, g) HCT116, h) HKE293T, i) KELLY, j) MHHNB11, k) NGP, l) RH30, m) SKES1, n) SKMEL2, o) SKNAS, p) SKNFI, q) SKNSH, r) TGW. Dose-response curves for each single-agent (top left), normalized cell death matrix (top middle), synergy matrix (top right; Note: units are δZIP), IC_{50} values of each single-agent (lower left), barplots corresponding to maximum synergy (lower middle; Note: dashed lines indicate expected values under additivity (black) or Bliss independence (red)), and scatterplot of cell death vs. synergy (lower right) for all 18 cell lines used in dense drug-drug screens.

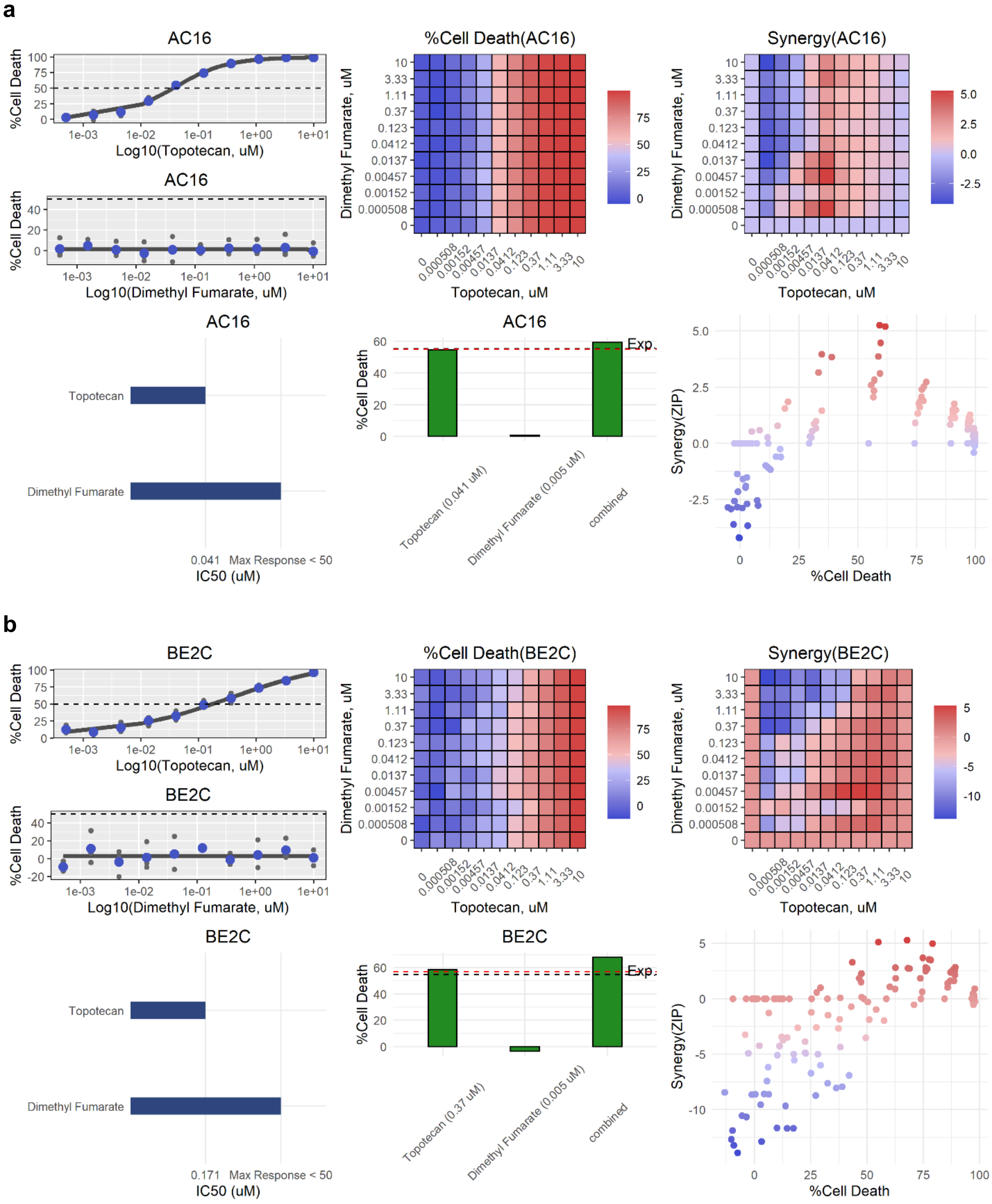
a
AC16
%Cell Death
Log10(Topotecan, uM)
AC16
%Cell Death
Log10(Dimethyl Fumarate, uM)
%Cell Death(AC16)
Dimethyl Fumarate, uM
Topotecan, uM
Synergy(AC16)
Dimethyl Fumarate, uM
Topotecan, uM
AC16
Topotecan
Dimethyl Fumarate
0.041
Max Response < 50
IC50 (uM)
AC16
%Cell Death
Exp.
Topotecan (0.041 uM)
Dimethyl Fumarate (0.005 uM)
combined
Synergy(ZIP)
%Cell Death
b
BE2C
%Cell Death
Log10(Topotecan, uM)
BE2C
%Cell Death
Log10(Dimethyl Fumarate, uM)
%Cell Death(BE2C)
Dimethyl Fumarate, uM
Topotecan, uM
Synergy(BE2C)
Dimethyl Fumarate, uM
Topotecan, uM
BE2C
Topotecan
Dimethyl Fumarate
0.171
Max Response < 50
IC50 (uM)
BE2C
%Cell Death
Exp.
Topotecan (0.37 uM)
Dimethyl Fumarate (0.005 uM)
combined
Synergy(ZIP)
%Cell Death

c

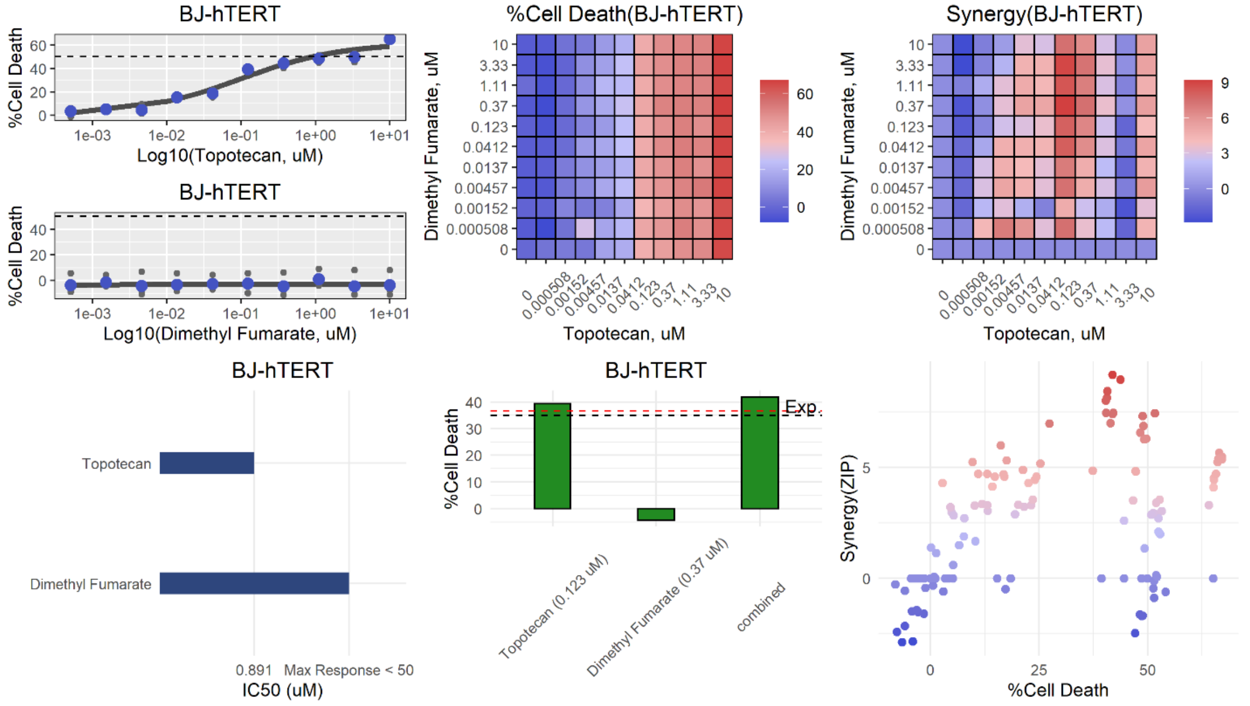

BJ-hTERT
%Cell Death
Log10(Topotecan, uM)
BJ-hTERT
%Cell Death
Log10(Dimethyl Fumarate, uM)
%Cell Death(BJ-hTERT)
Dimethyl Fumarate, uM
Topotecan, uM
Synergy(BJ-hTERT)
Dimethyl Fumarate, uM
Topotecan, uM
BJ-hTERT
Topotecan
Dimethyl Fumarate
0.891 Max Response < 50
IC50 (uM)
BJ-hTERT
%Cell Death
Exp
Topotecan (0.123 uM)
Dimethyl Fumarate (0.37 uM)
combined
Synergy(ZIP)
%Cell Death

d

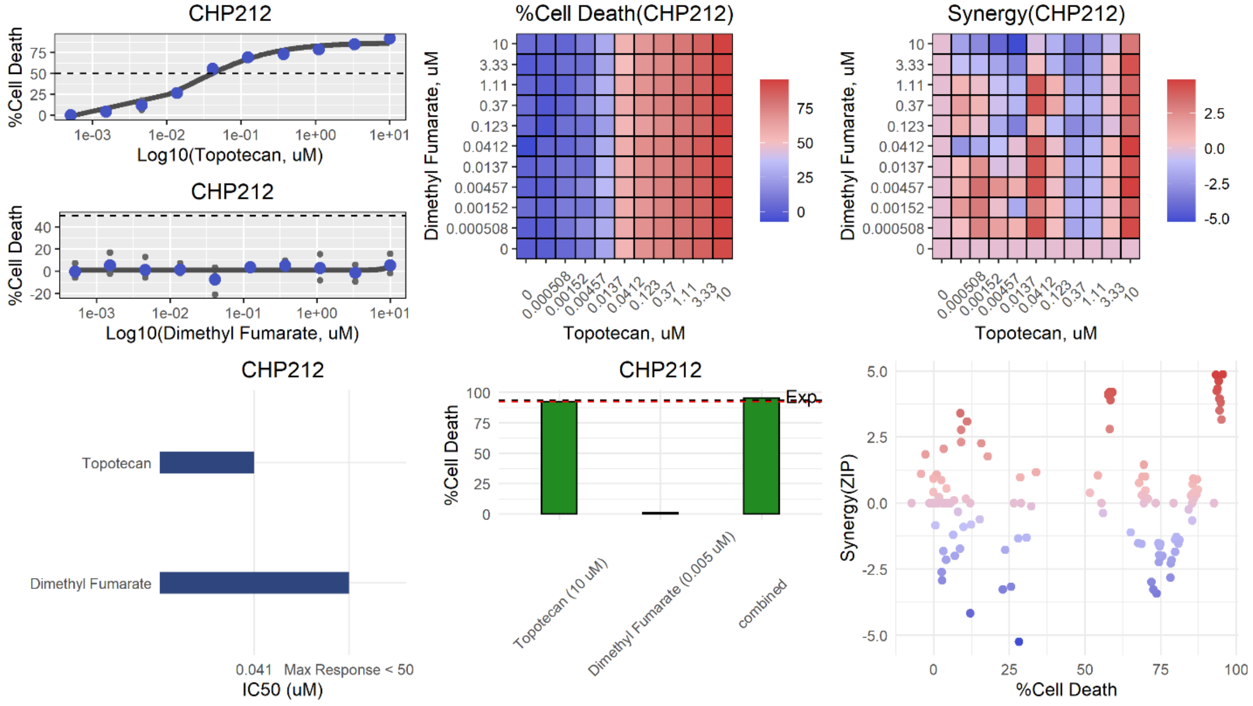

CHP212
%Cell Death
Log10(Topotecan, uM)
CHP212
%Cell Death
Log10(Dimethyl Fumarate, uM)
%Cell Death(CHP212)
Dimethyl Fumarate, uM
Topotecan, uM
Synergy(CHP212)
Dimethyl Fumarate, uM
Topotecan, uM
CHP212
Topotecan
Dimethyl Fumarate
0.041 Max Response < 50
IC50 (uM)
CHP212
%Cell Death
Exp
Topotecan (10 uM)
Dimethyl Fumarate (0.005 uM)
combined
Synergy(ZIP)
%Cell Death

e

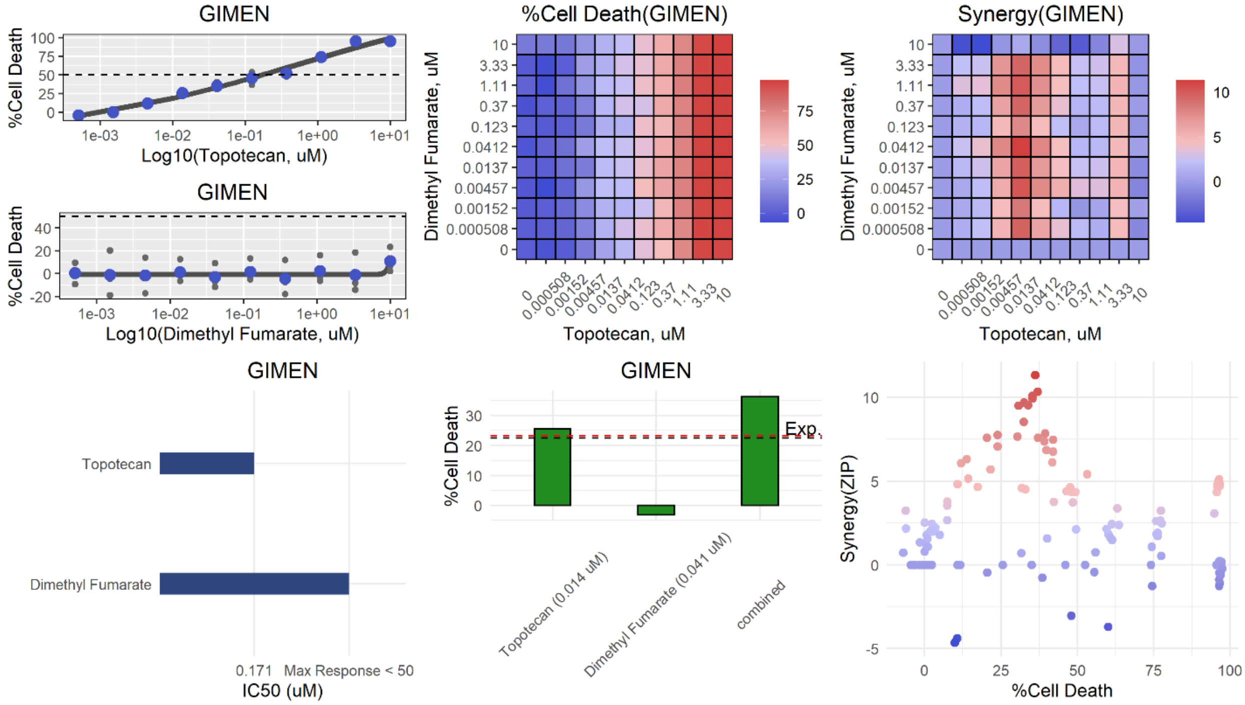
GIMEN
%Cell Death
Log10(Topotecan, uM)
GIMEN
%Cell Death
Log10(Dimethyl Fumarate, uM)
%Cell Death(GIMEN)
Dimethyl Fumarate, uM
Topotecan, uM
Synergy(GIMEN)
Dimethyl Fumarate, uM
Topotecan, uM
GIMEN
Topotecan
Dimethyl Fumarate
0.171 Max Response < 50
IC50 (uM)
GIMEN
%Cell Death
Exp.
Topotecan (0.014 uM)
Dimethyl Fumarate (0.041 uM)
combined
Synergy(ZIP)
%Cell Death

f

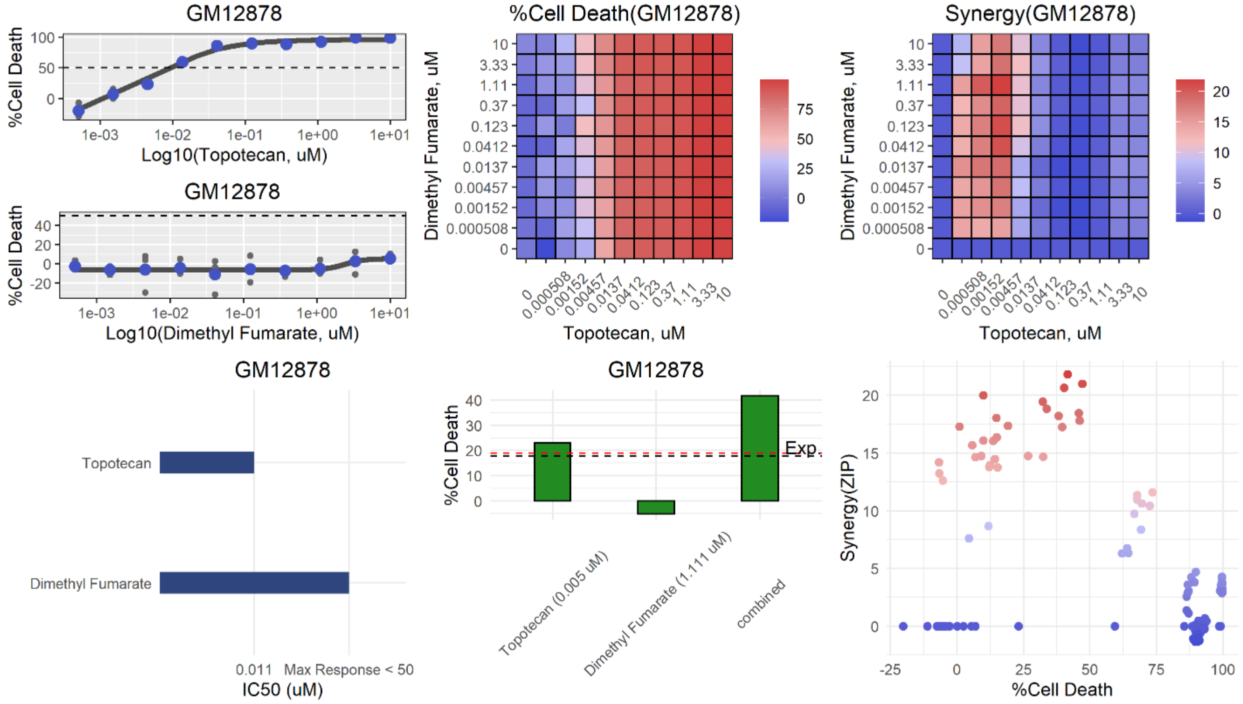
GM12878
%Cell Death
Log10(Topotecan, uM)
GM12878
%Cell Death
Log10(Dimethyl Fumarate, uM)
%Cell Death(GM12878)
Dimethyl Fumarate, uM
Topotecan, uM
Synergy(GM12878)
Dimethyl Fumarate, uM
Topotecan, uM
GM12878
Topotecan
Dimethyl Fumarate
0.011 Max Response < 50
IC50 (uM)
GM12878
%Cell Death
Exp.
Topotecan (0.005 uM)
Dimethyl Fumarate (1.111 uM)
combined
Synergy(ZIP)
%Cell Death

g

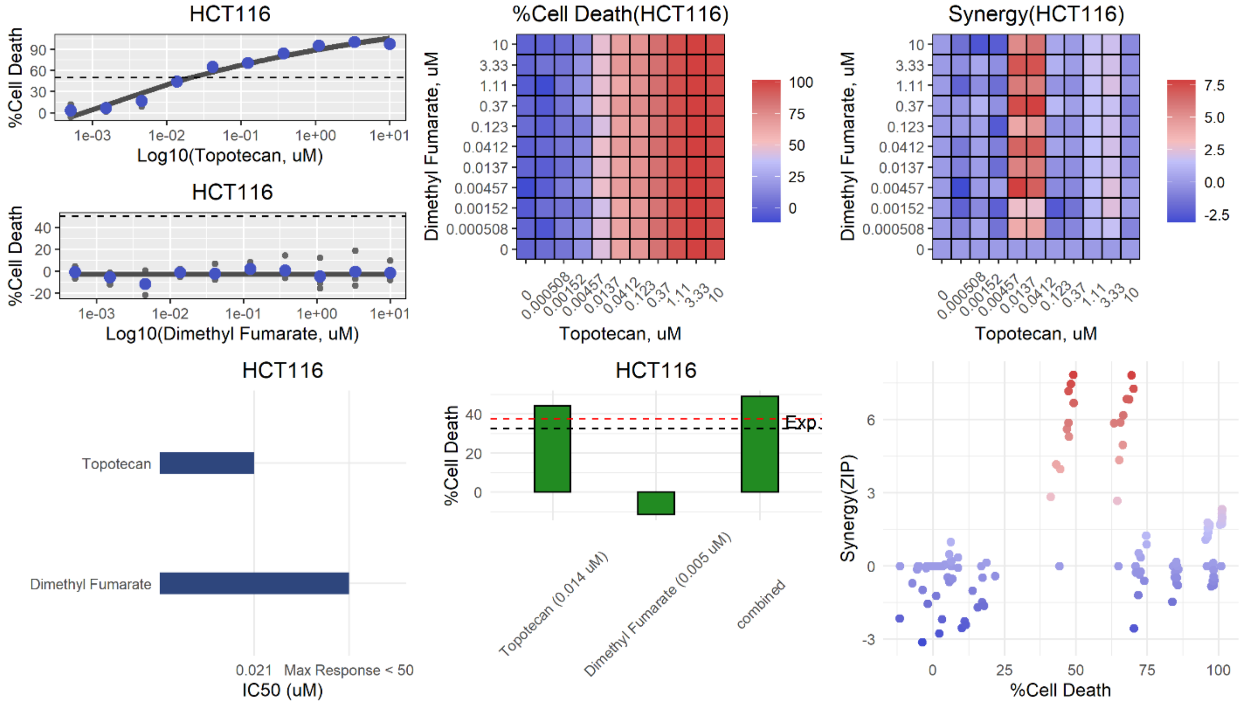
HCT116
%Cell Death
Log10(Topotecan, uM)
HCT116
%Cell Death
Log10(Dimethyl Fumarate, uM)
%Cell Death(HCT116)
Dimethyl Fumarate, uM
Topotecan, uM
Synergy(HCT116)
Dimethyl Fumarate, uM
Topotecan, uM
HCT116
Topotecan
Dimethyl Fumarate
0.021
Max Response < 50
IC50 (uM)
HCT116
%Cell Death
Exp.
Topotecan (0.014 uM)
Dimethyl Fumarate (0.005 uM)
combined
Synergy(ZIP)
%Cell Death

h

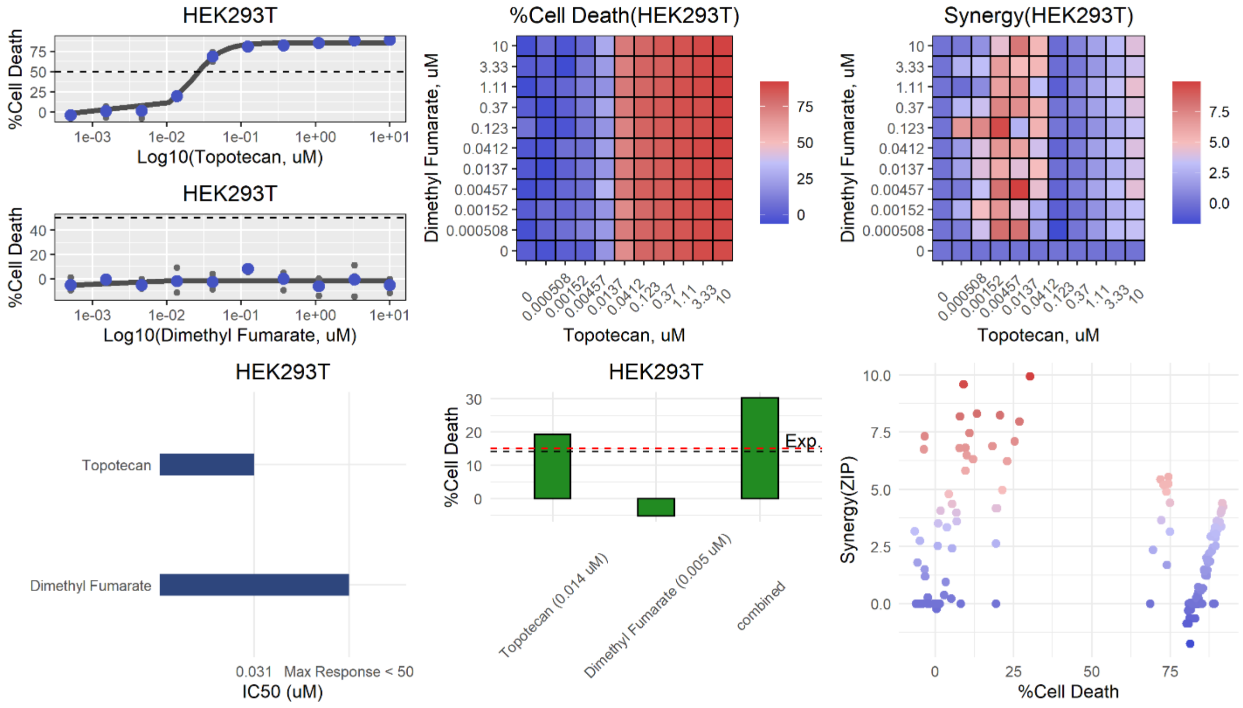
HEK293T
%Cell Death
Log10(Topotecan, uM)
HEK293T
%Cell Death
Log10(Dimethyl Fumarate, uM)
%Cell Death(HEK293T)
Dimethyl Fumarate, uM
Topotecan, uM
Synergy(HEK293T)
Dimethyl Fumarate, uM
Topotecan, uM
HEK293T
Topotecan
Dimethyl Fumarate
0.031
Max Response < 50
IC50 (uM)
HEK293T
%Cell Death
Exp.
Topotecan (0.014 uM)
Dimethyl Fumarate (0.005 uM)
combined
Synergy(ZIP)
%Cell Death

i

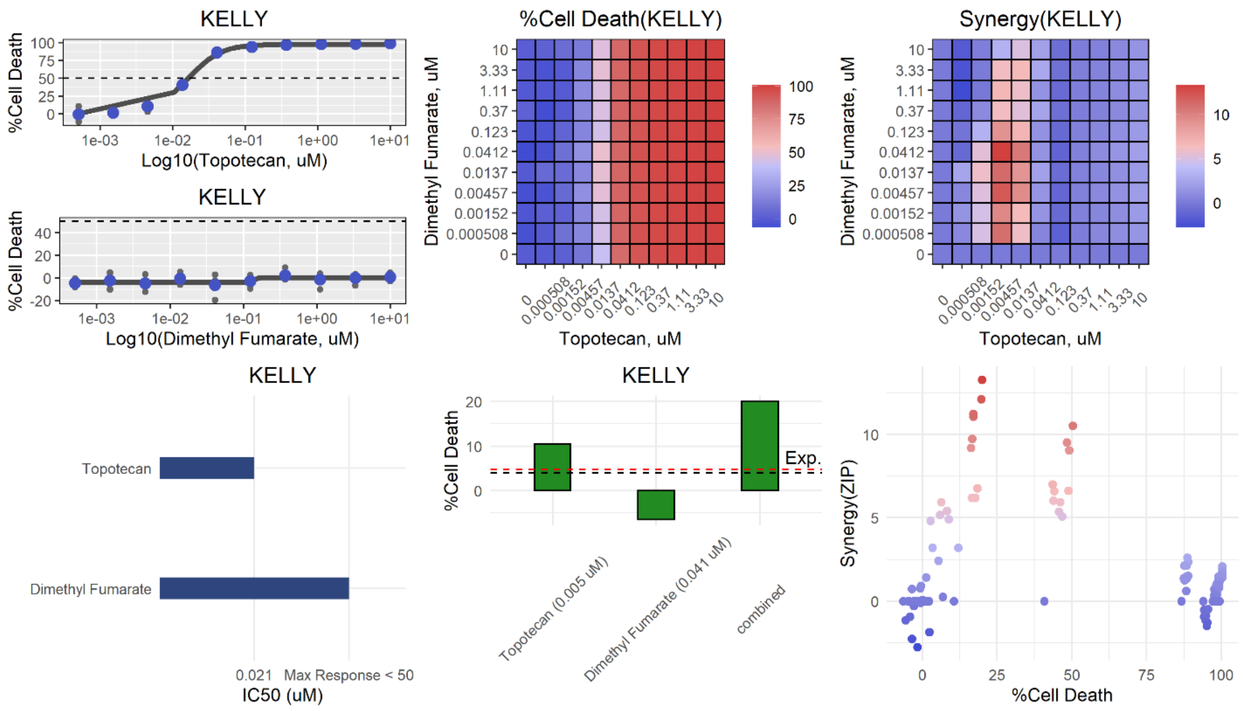
KELLY
%Cell Death
100
75
50
25
0
1e-03
1e-02
1e-01
1e+00
1e+01
Log10(Topotecan, uM)
KELLY
%Cell Death
40
20
0
-20
Log10(Dimethyl Fumarate, uM)
%Cell Death(KELLY)
Dimethyl Fumarate, uM
10
3.33
1.11
0.37
0.123
0.0412
0.0137
0.00457
0.00152
0.000508
0
Topotecan, uM
Synergy(KELLY)
10
5
0
KELLY
Topotecan
Dimethyl Fumarate
0.021
Max Response < 50
IC50 (uM)
KELLY
%Cell Death
Exp.
Topotecan (0.005 uM)
Dimethyl Fumarate (0.041 uM)
combined
Synergy(ZIP)
%Cell Death

j

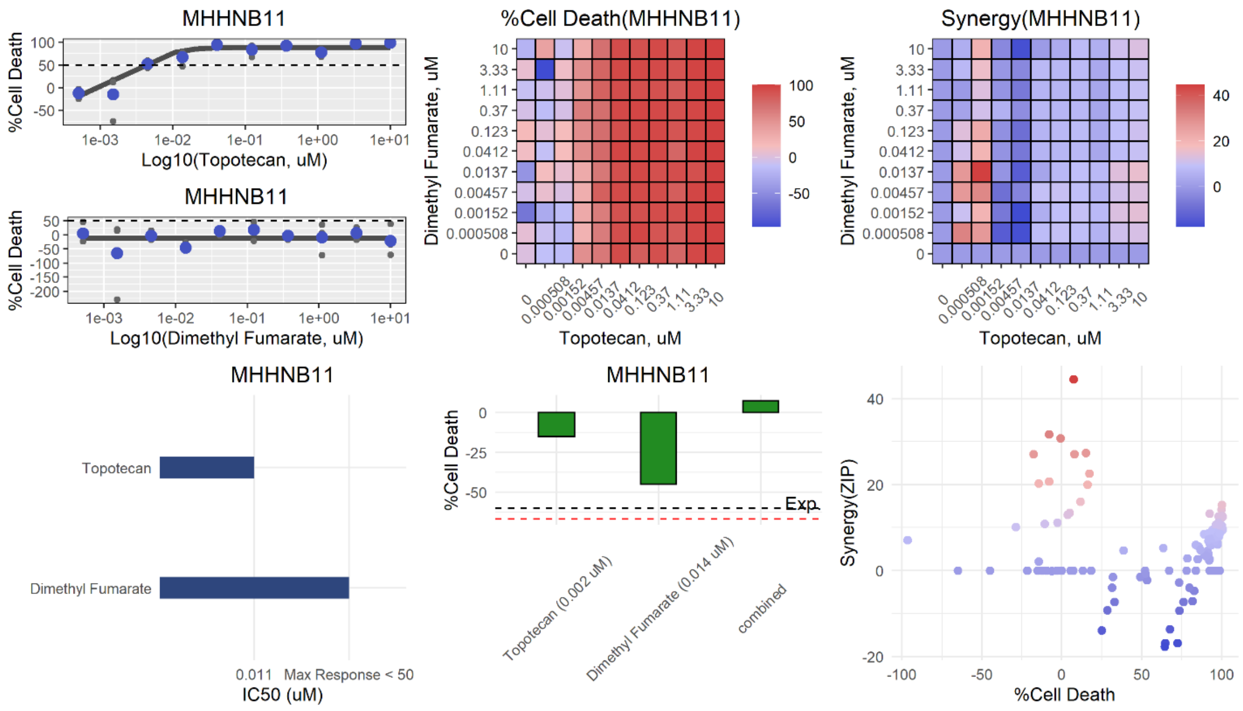
MHHNB11
%Cell Death
100
50
0
-50
1e-03
1e-02
1e-01
1e+00
1e+01
Log10(Topotecan, uM)
MHHNB11
%Cell Death
50
0
-50
-100
-150
-200
Log10(Dimethyl Fumarate, uM)
%Cell Death(MHHNB11)
Dimethyl Fumarate, uM
Topotecan, uM
Synergy(MHHNB11)
40
20
0
MHHNB11
Topotecan
Dimethyl Fumarate
0.011
Max Response < 50
IC50 (uM)
MHHNB11
%Cell Death
0
-25
-50
Exp.
Topotecan (0.002 uM)
Dimethyl Fumarate (0.014 uM)
combined
Synergy(ZIP)
%Cell Death
-100
-50
0
50
100

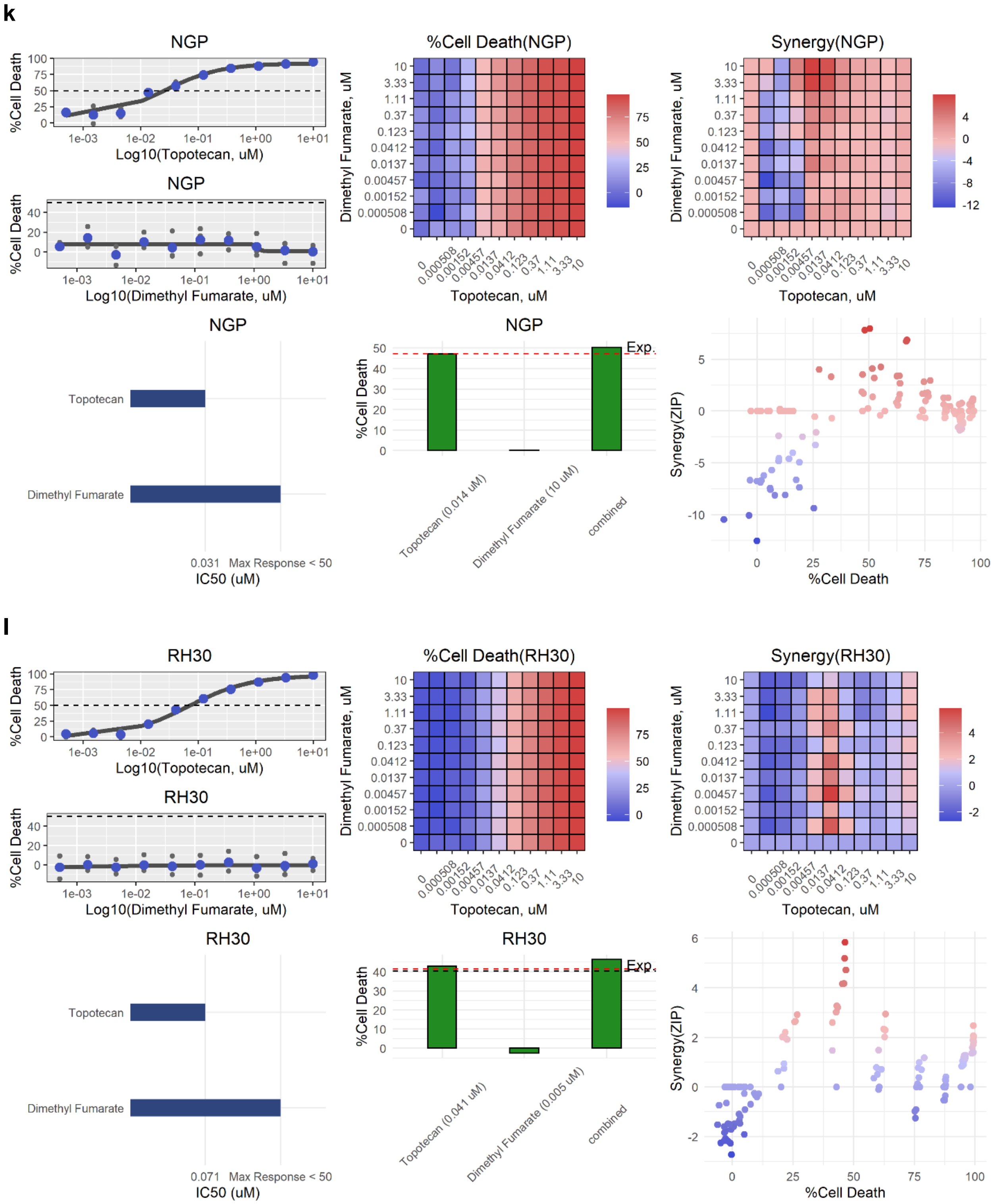
k
NGP
%Cell Death
Log10(Topotecan, uM)
Log10(Dimethyl Fumarate, uM)
%Cell Death(NGP)
Dimethyl Fumarate, uM
Topotecan, uM
Synergy(NGP)
Topotecan
Dimethyl Fumarate
0.031
Max Response < 50
IC50 (uM)
Topotecan (0.014 uM)
Dimethyl Fumarate (10 uM)
combined
Exp.
Synergy(ZIP)
l
RH30
%Cell Death(RH30)
Synergy(RH30)
0.071
Topotecan (0.041 uM)
Dimethyl Fumarate (0.005 uM)

m

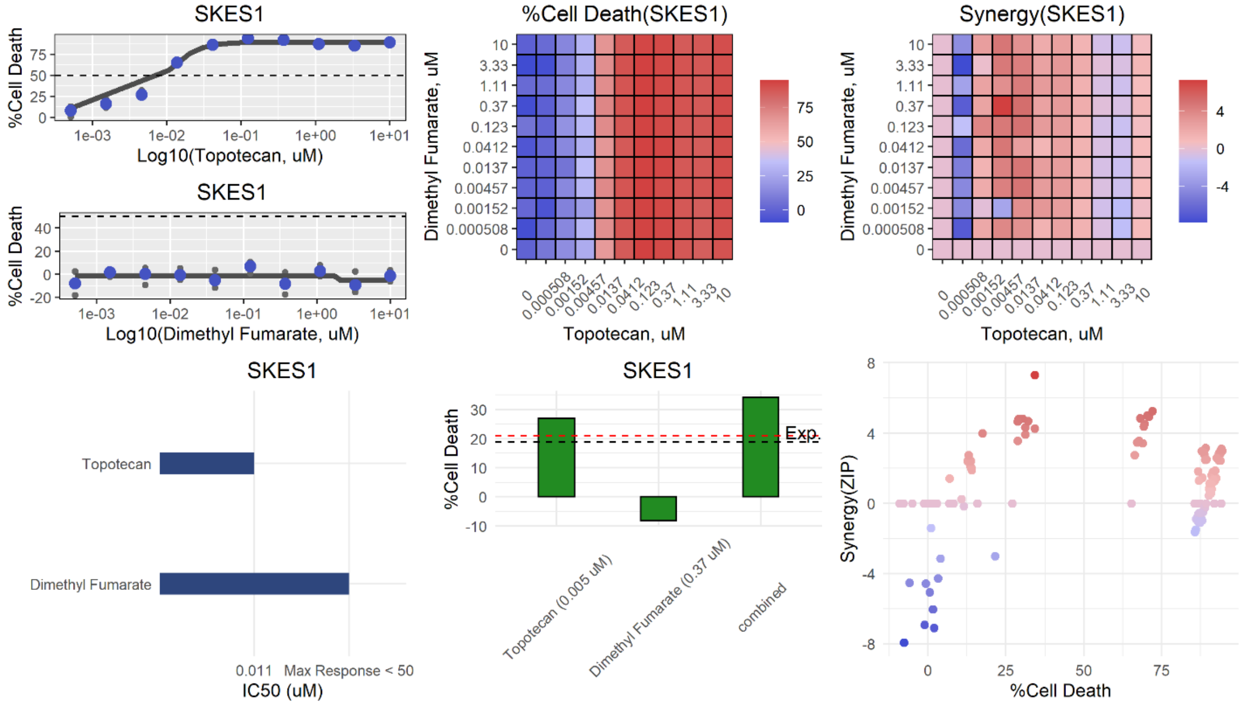
SKES1
%Cell Death
Log10(Topotecan, uM)
SKES1
Log10(Dimethyl Fumarate, uM)
%Cell Death(SKES1)
Dimethyl Fumarate, uM
Topotecan, uM
Synergy(SKES1)
SKES1
Topotecan
Dimethyl Fumarate
0.011
Max Response < 50
IC50 (uM)
SKES1
Exp.
Topotecan (0.005 uM)
Dimethyl Fumarate (0.37 uM)
combined
Synergy(ZIP)
%Cell Death

n

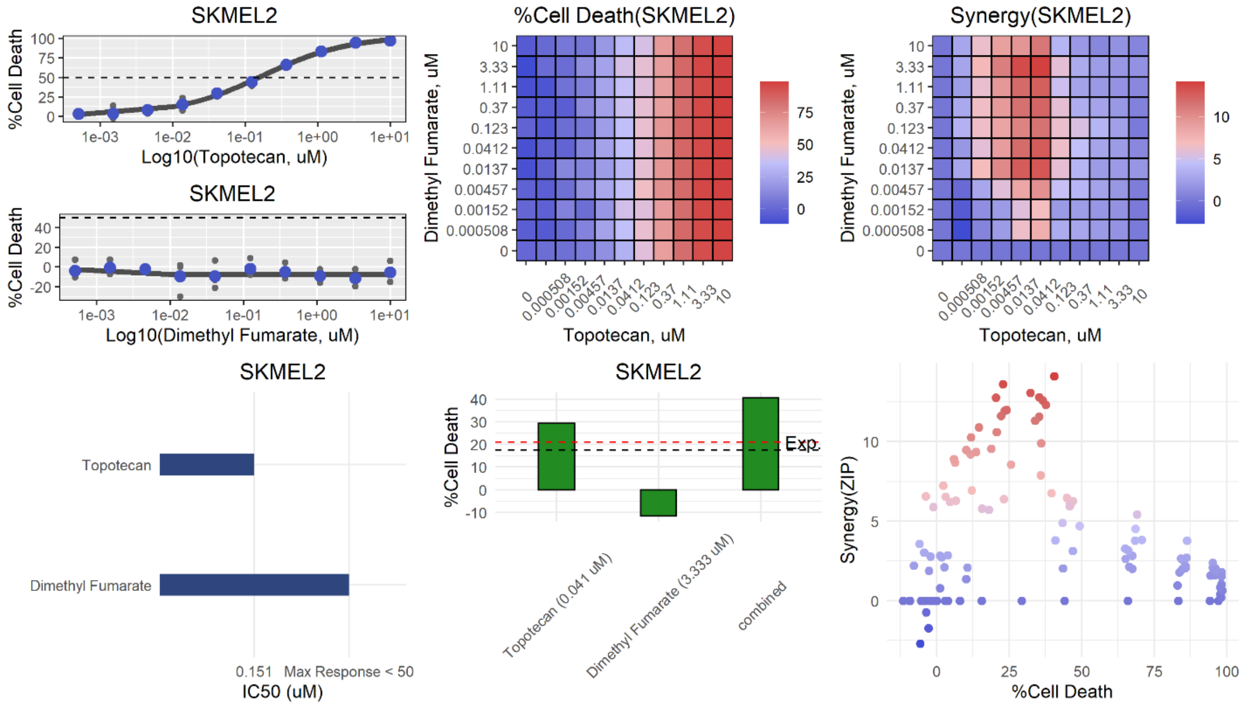
SKMEL2
%Cell Death
Log10(Topotecan, uM)
SKMEL2
Log10(Dimethyl Fumarate, uM)
%Cell Death(SKMEL2)
Dimethyl Fumarate, uM
Topotecan, uM
Synergy(SKMEL2)
SKMEL2
Topotecan
Dimethyl Fumarate
0.151
Max Response < 50
IC50 (uM)
SKMEL2
Exp.
Topotecan (0.041 uM)
Dimethyl Fumarate (3.333 uM)
combined
Synergy(ZIP)
%Cell Death

o

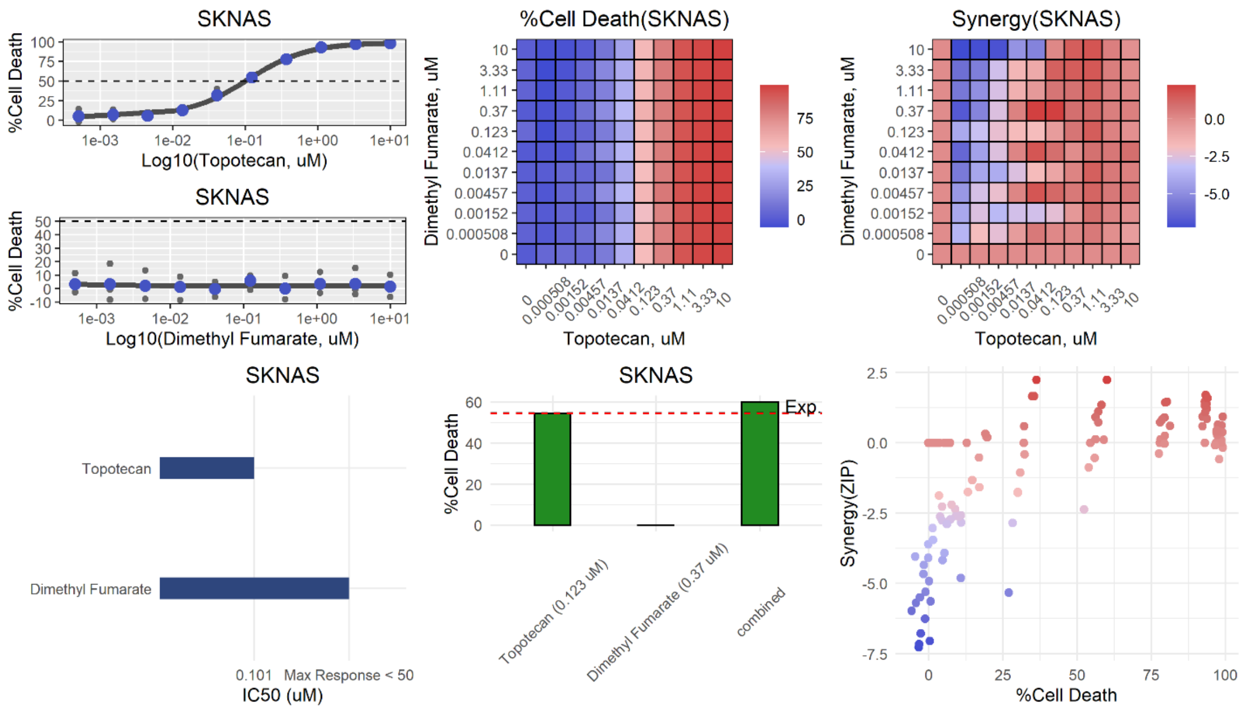
SKNAS
%Cell Death
Log10(Topotecan, uM)
SKNAS
%Cell Death
Log10(Dimethyl Fumarate, uM)
%Cell Death(SKNAS)
Dimethyl Fumarate, uM
Topotecan, uM
Synergy(SKNAS)
Dimethyl Fumarate, uM
Topotecan, uM
SKNAS
Topotecan
Dimethyl Fumarate
0.101
Max Response < 50
IC50 (uM)
SKNAS
%Cell Death
Exp.
Topotecan (0.123 uM)
Dimethyl Fumarate (0.37 uM)
combined
Synergy(ZIP)
%Cell Death

p

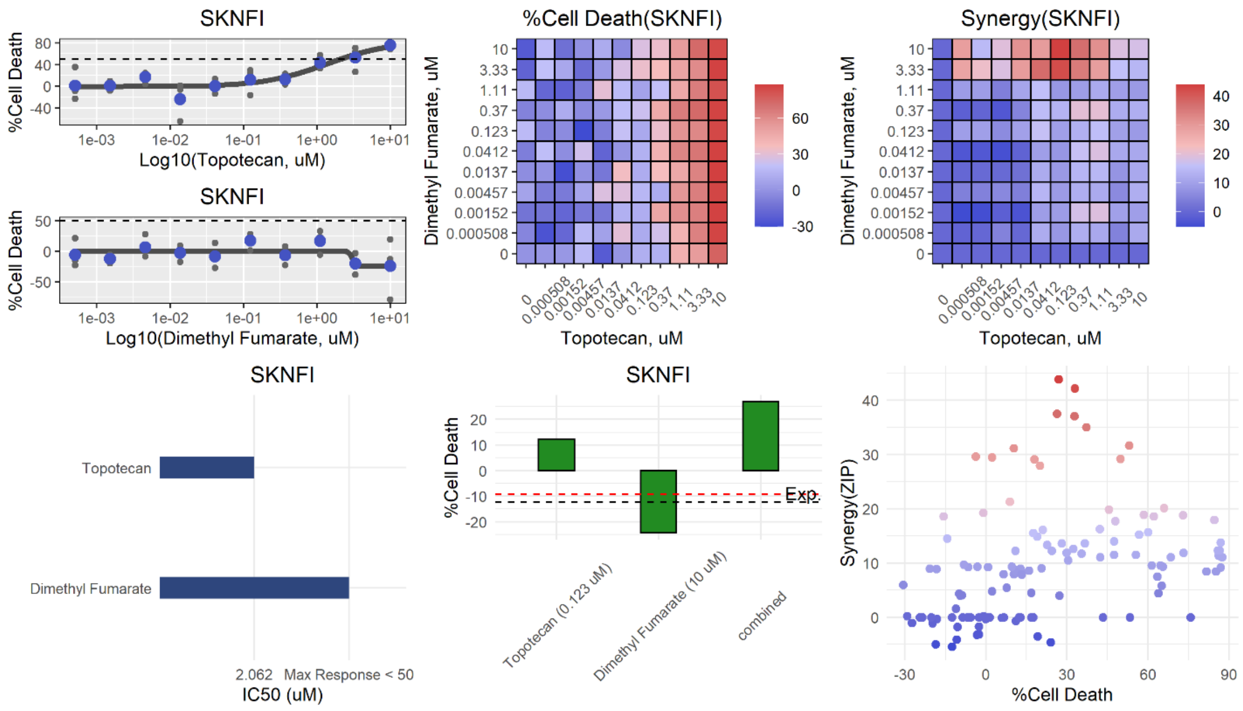
SKNFI
%Cell Death
Log10(Topotecan, uM)
SKNFI
%Cell Death
Log10(Dimethyl Fumarate, uM)
%Cell Death(SKNFI)
Dimethyl Fumarate, uM
Topotecan, uM
Synergy(SKNFI)
Dimethyl Fumarate, uM
Topotecan, uM
SKNFI
Topotecan
Dimethyl Fumarate
2.062
Max Response < 50
IC50 (uM)
SKNFI
%Cell Death
Exp.
Topotecan (0.123 uM)
Dimethyl Fumarate (10 uM)
combined
Synergy(ZIP)
%Cell Death

q

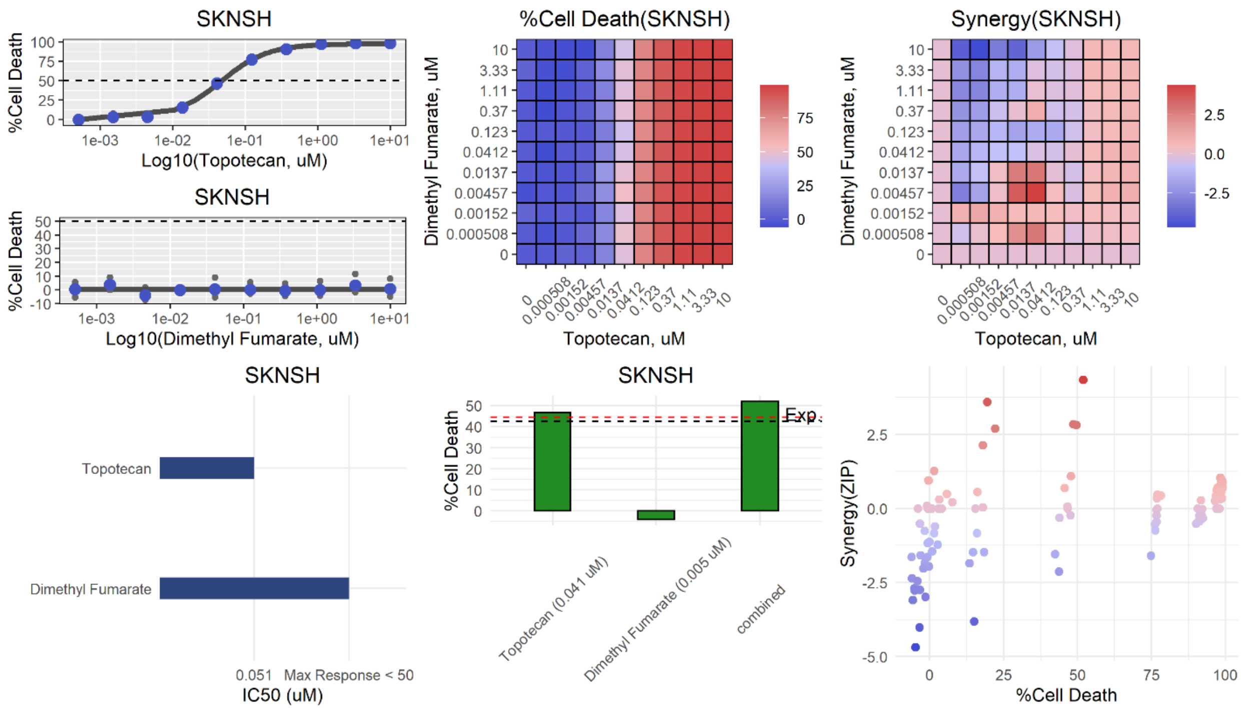

SKNSH
%Cell Death
Log10(Topotecan, uM)
SKNSH
%Cell Death
Log10(Dimethyl Fumarate, uM)
%Cell Death(SKNSH)
Dimethyl Fumarate, uM
Topotecan, uM
Synergy(SKNSH)
Dimethyl Fumarate, uM
Topotecan, uM
SKNSH
Topotecan
Dimethyl Fumarate
0.051
Max Response < 50
IC50 (uM)
SKNSH
%Cell Death
Exp.
Topotecan (0.041 uM)
Dimethyl Fumarate (0.005 uM)
combined
Synergy(ZIP)
%Cell Death

r

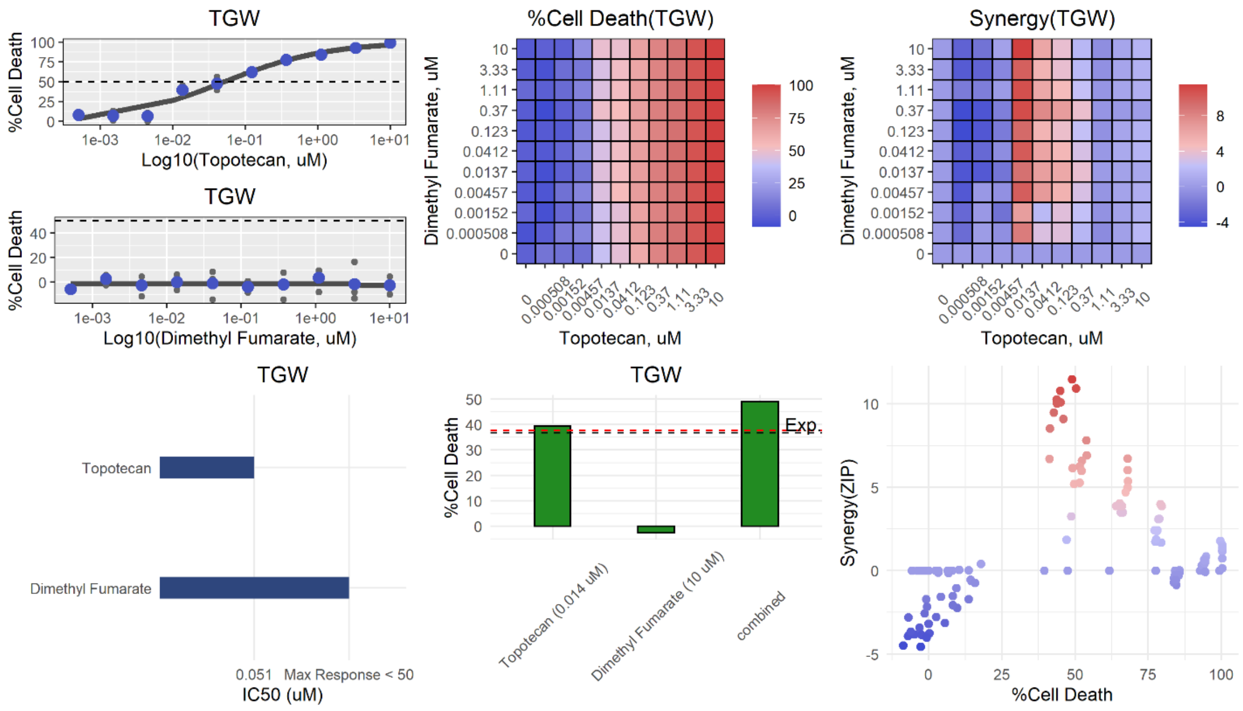

TGW
%Cell Death
Log10(Topotecan, uM)
TGW
%Cell Death
Log10(Dimethyl Fumarate, uM)
%Cell Death(TGW)
Dimethyl Fumarate, uM
Topotecan, uM
Synergy(TGW)
Dimethyl Fumarate, uM
Topotecan, uM
TGW
Topotecan
Dimethyl Fumarate
0.051
Max Response < 50
IC50 (uM)
TGW
%Cell Death
Exp.
Topotecan (0.014 uM)
Dimethyl Fumarate (10 uM)
combined
Synergy(ZIP)
%Cell Death

Supplementary Figure 8. Summary of dense topotecan-dimethly fumarate combinations in all cell lines.

(a - r) 18 panels for combination of topotecan and dimethyl fumarate in all 18 cell lines. a) AC16, b) BE2C, c) BJ-TERT, d) CHP212, e) GIMEN, f) GM12878, g) HCT116, h) HKE293T, i) KELLY, j) MHHNB11, k) NGP, l) RH30, m) SKES1, n) SKMEL2, o) SKNAS, p) SKNFI, q) SKNSH, r) TGW. Dose-response curves for each single-agent (top left), normalized cell death matrix (top middle), synergy matrix (top right; Note: units are δZIP), IC_{50} values of each single-agent (lower left), barplots corresponding to maximum synergy (lower middle; Note: dashed lines indicate expected values under additivity (black) or Bliss independence (red)), and scatterplot of cell death vs. synergy (lower right) for all 18 cell lines used in dense drug-drug screens.

a

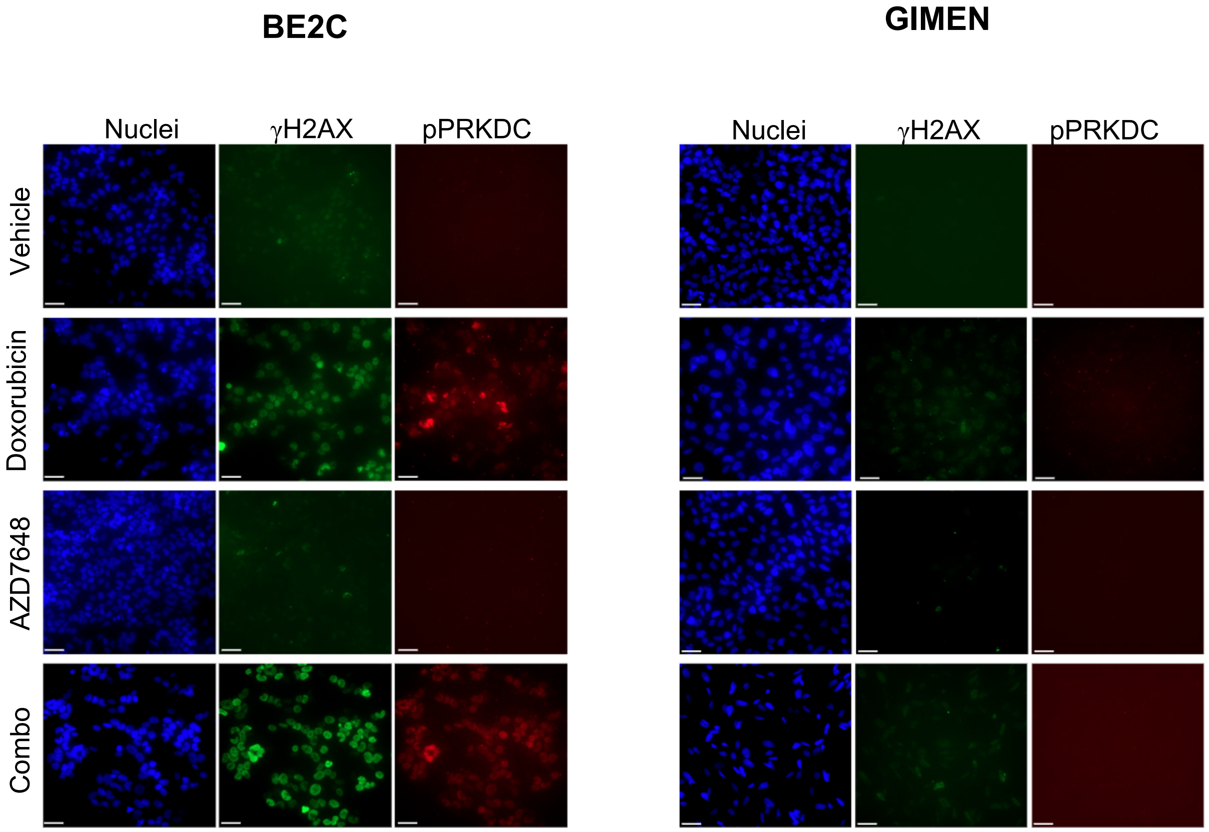
BE2C
GIMEN
Nuclei
γH2AX
pPRKDC
Vehicle
Doxorubicin
AZD7648
Combo

b

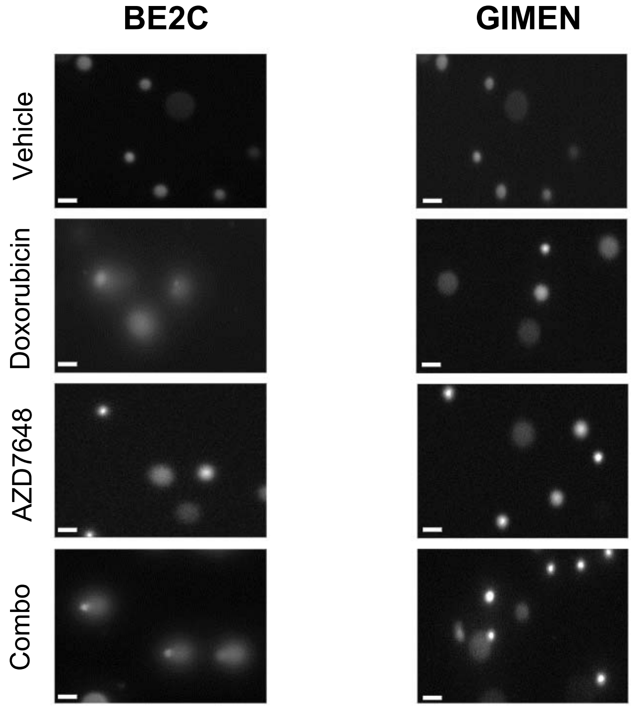
BE2C
GIMEN
Vehicle
Doxorubicin
AZD7648
Combo

Supplementary Figure 9. DNA damages in BE2C and GIMEN

After 72 hr vehicle or drug treatment, γH2AX and phosphorylated PRKDC were visualized by the representatives (a, 3 imgaes per vehicle or treatment, two independnet, scale bar 100μm) and tail moment was visualized by the representatives (b, 5 images per vehile or treatment, two independent,scale bar 20μm).

a

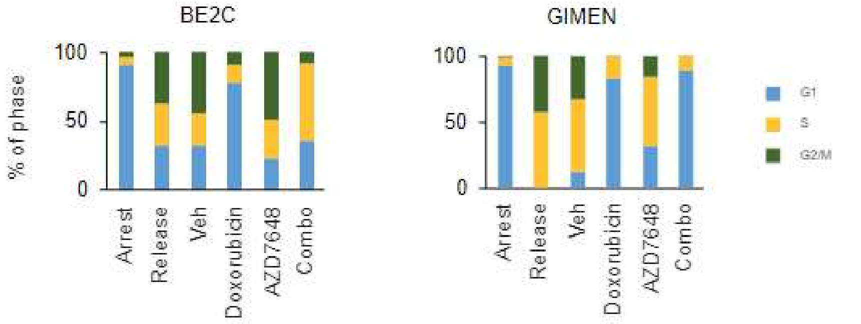

b

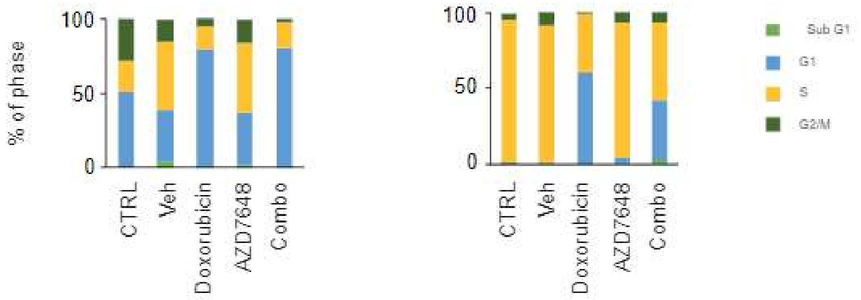

Supplementary Figure 10. Cell cycle analysis in BE2C and GIMEN

After 48 hr serum starvation, the cells were arrested at G0/G1 (a) and without cell synchronization (b), followed by vehicle or drug treatment for 72 hr (n=3 per group (veh or drug treatment)). After 72 hr, the cells were analyzed by FACS.

a

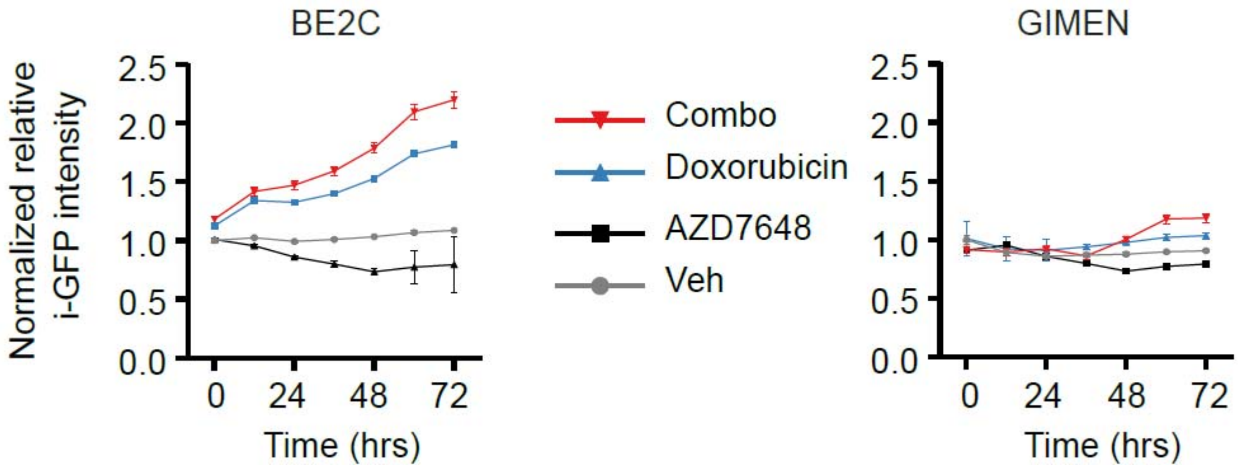

b

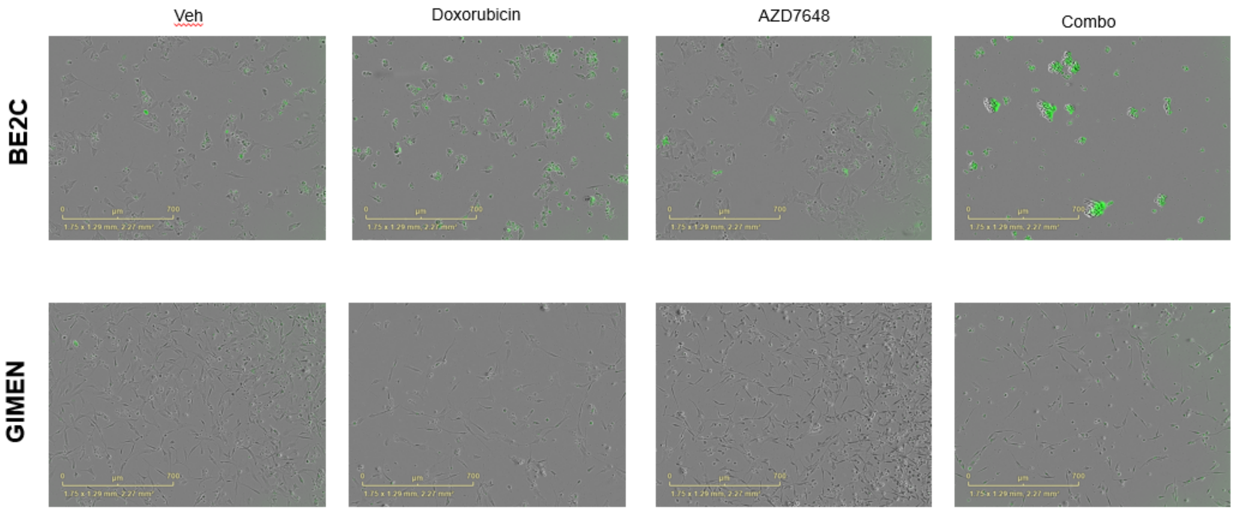

Supplementary Figure 11. NHEJ activity in BE2C and GIMEN

(a) Line plot showing the results of a cell-based assay for NHEJ using i-GFP quantifying the activity of NHEJ (y axis) in BE2C and GIMEN cells over 72h (x axis) at 12h intervals (Data presented as mean ±SEM, n=25 of relative intensiy of green per group (vehicle, doxorubicin, AZD7648, and combo), two independent).

(b) Images showing the changes in i-GFP, an indicator of NHEJ 72 hr after vehicle or drug treatment.

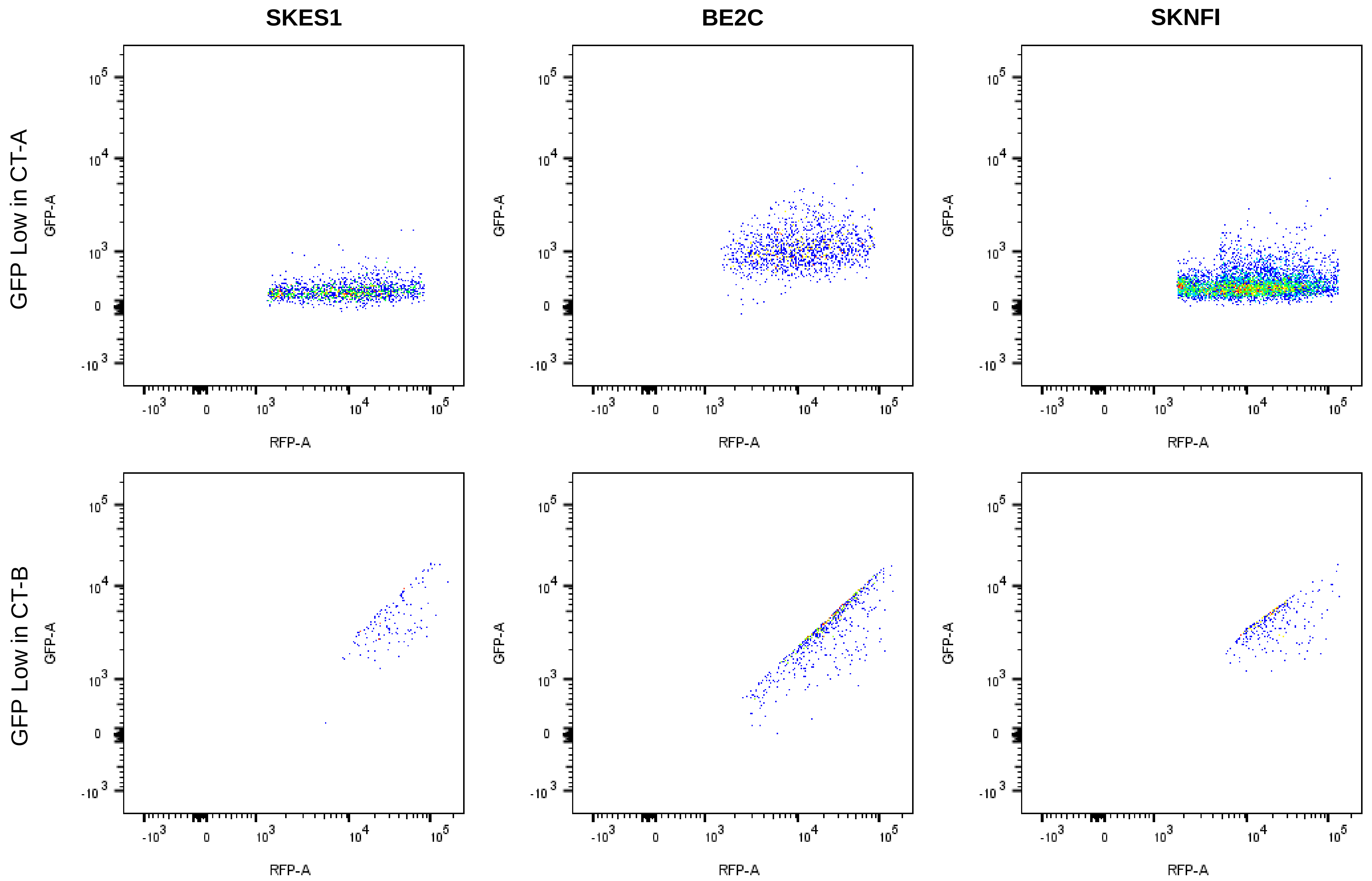
SKES1
BE2C
SKNFI
GFP Low in CT-A
GFP Low in CT-B
GFP-A
RFP-A
10^5
10^4
10^3
0
-10^3

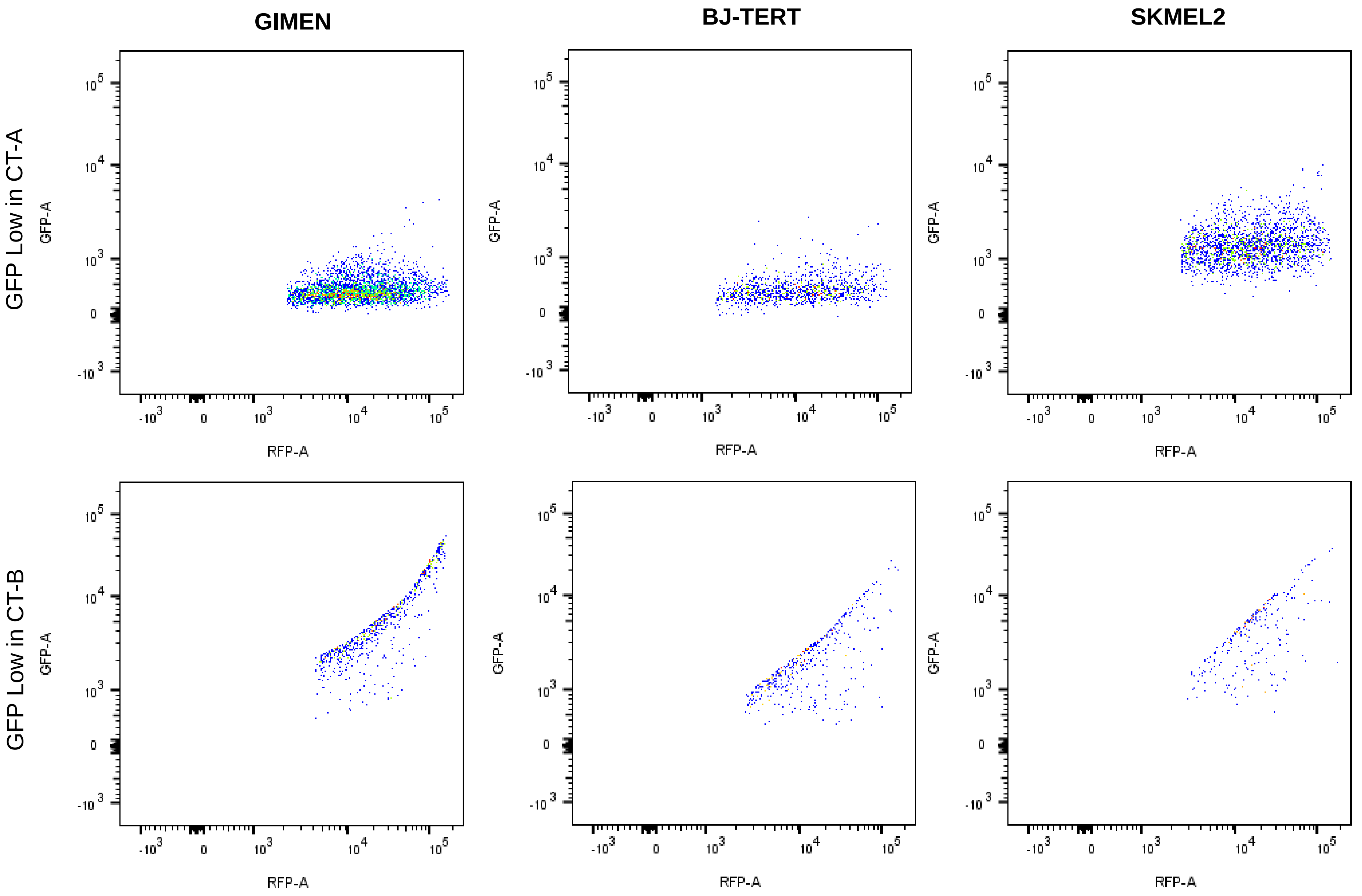
GIMEN
BJ-TERT
SKMEL2
GFP Low in CT-A
GFP Low in CT-B
GFP-A
RFP-A

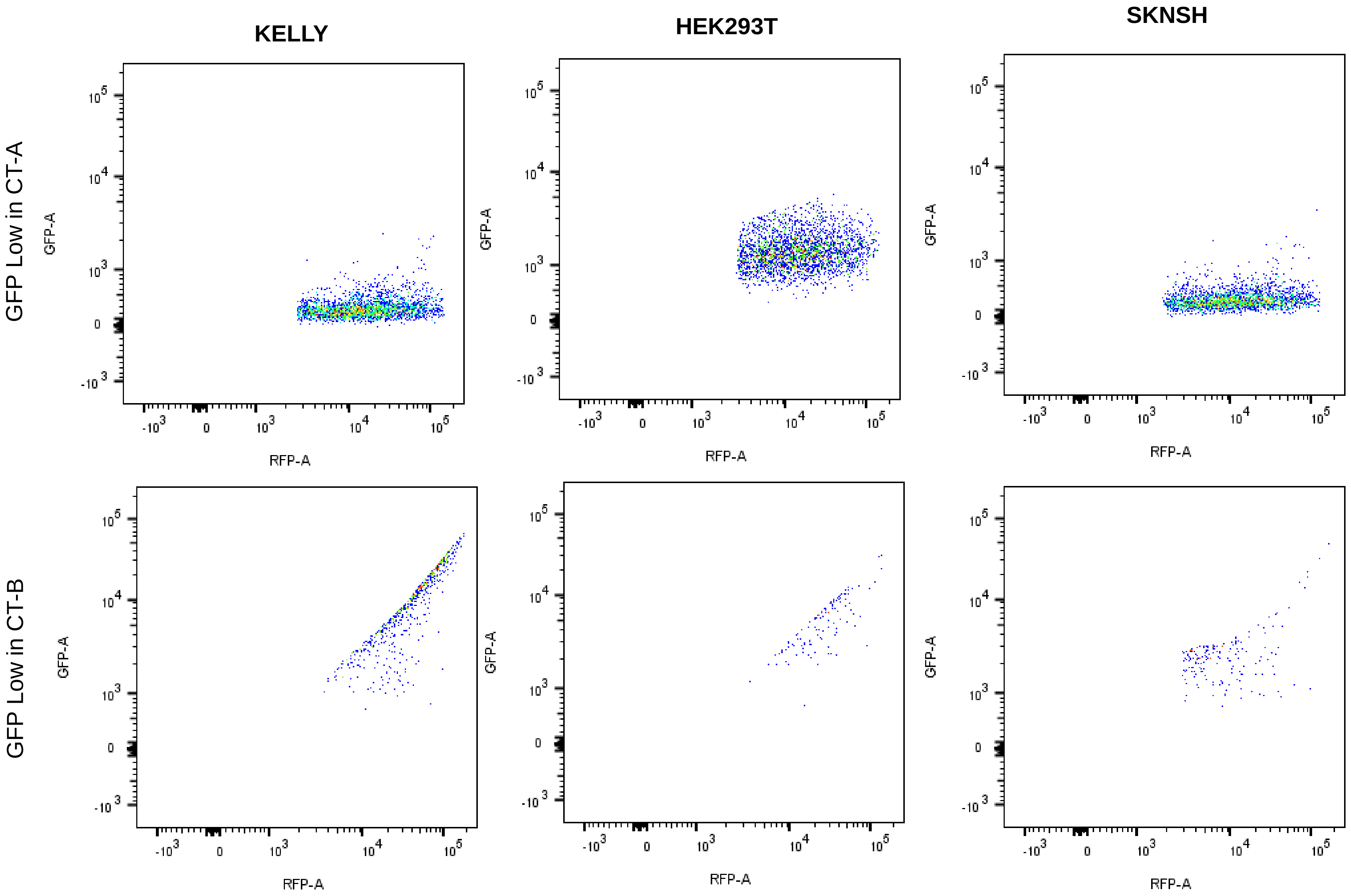

KELLY
HEK293T
SKNSH
GFP Low in CT-A
GFP Low in CT-B
GFP-A
RFP-A

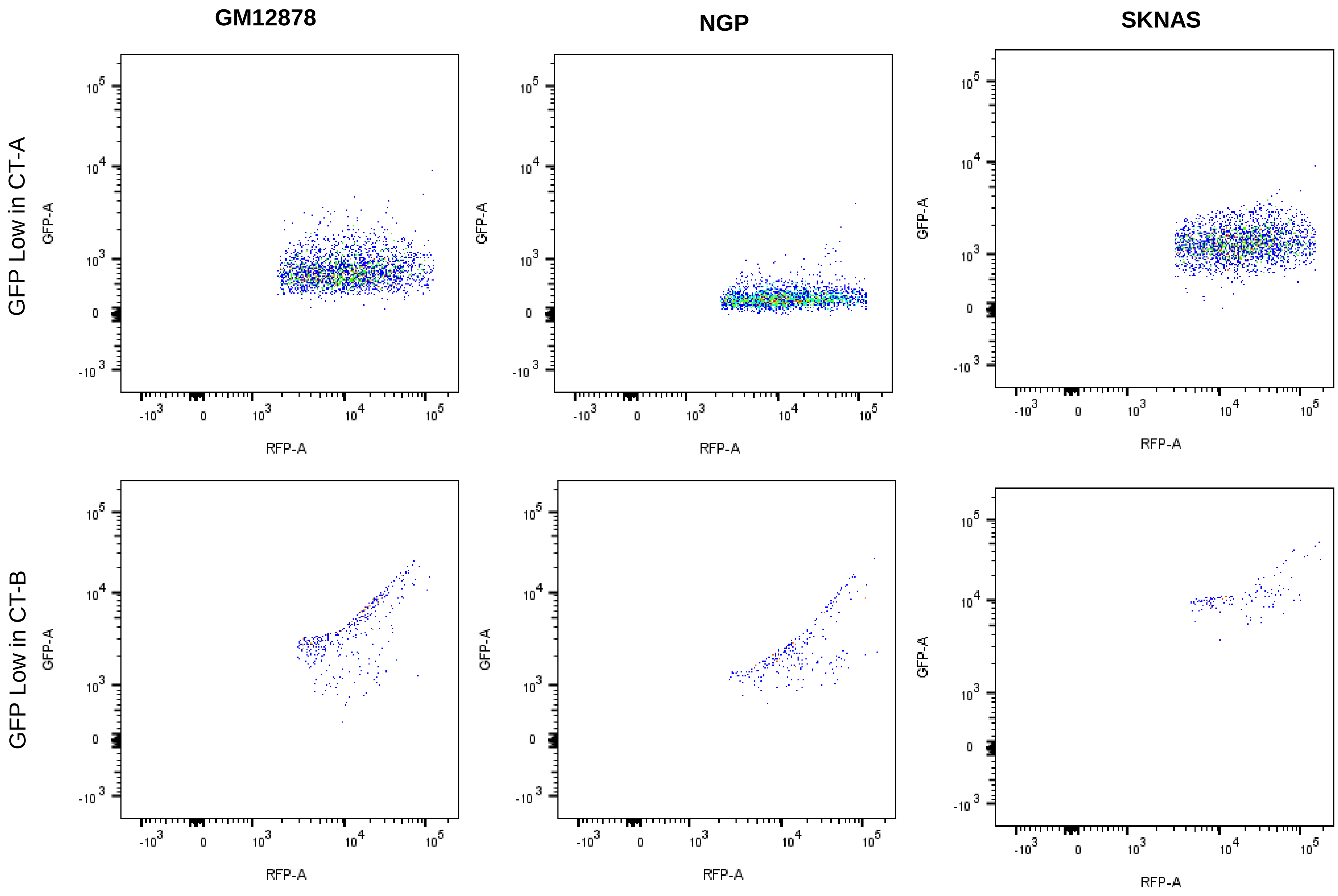

GM12878
NGP
SKNAS
GFP Low in CT-A
GFP Low in CT-B
GFP-A
RFP-A

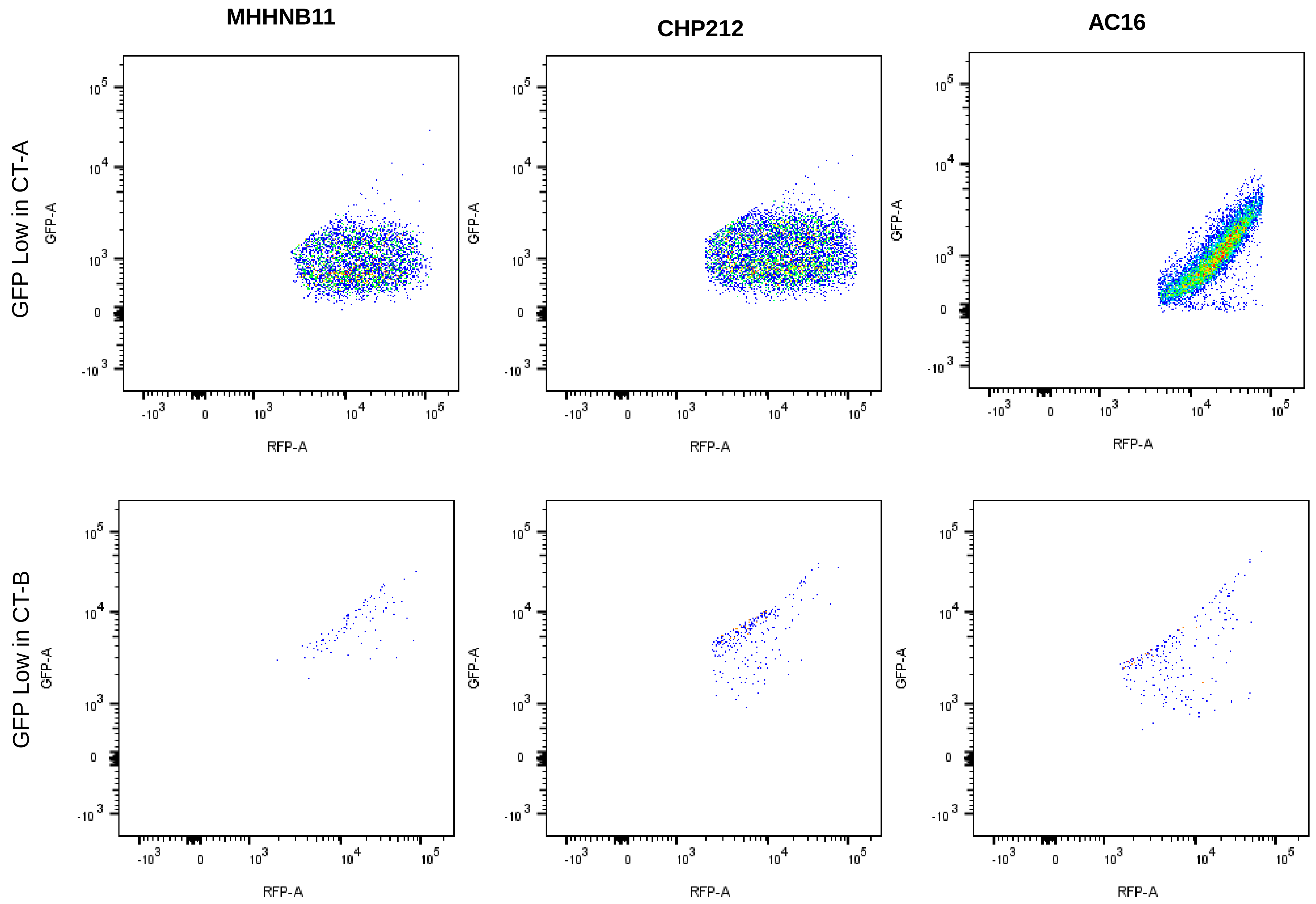
MHHNB11
CHP212
AC16
GFP Low in CT-A
GFP Low in CT-B
GFP-A
RFP-A
10^5
10^4
10^3
0
-10^3

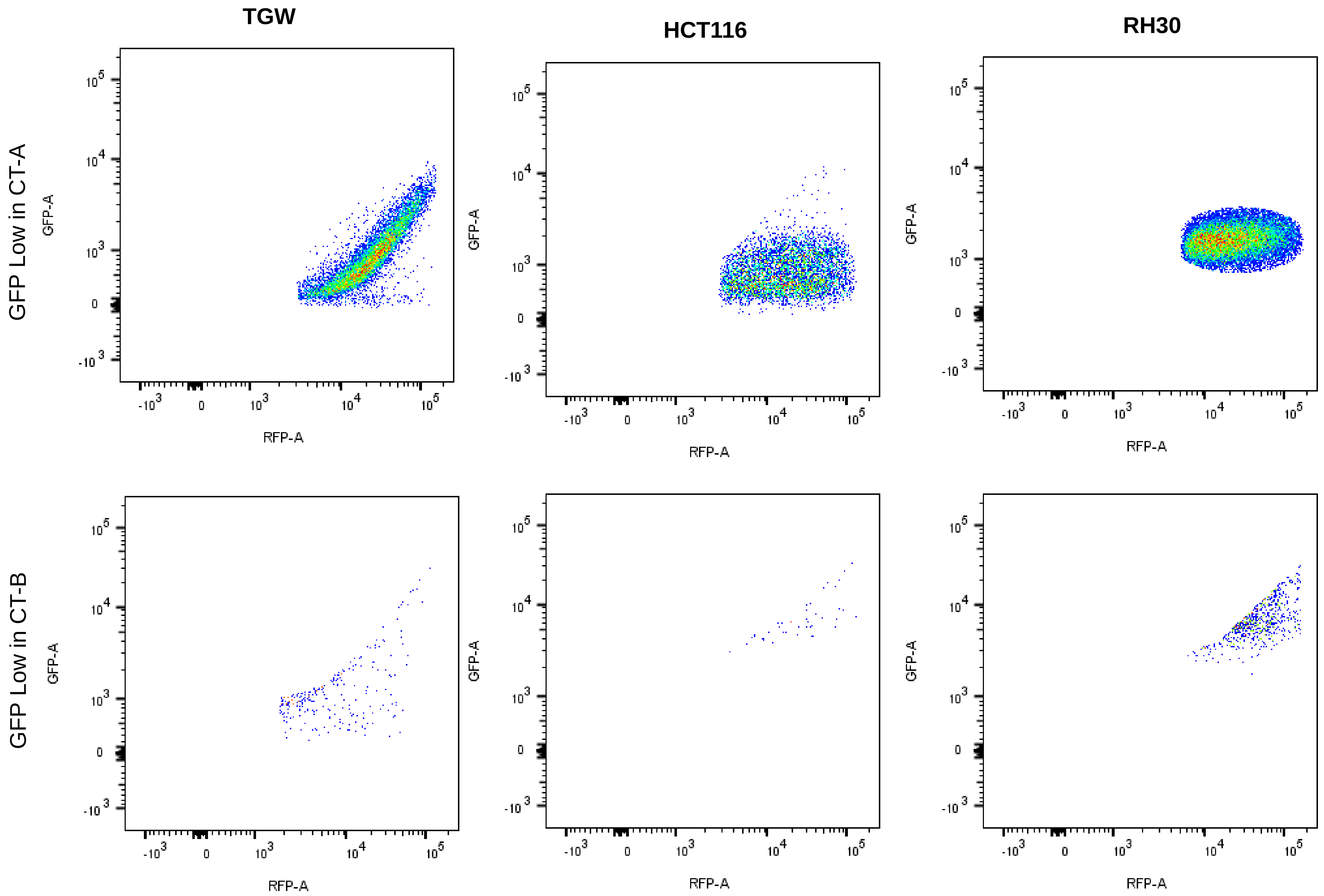
TGW
HCT116
RH30
GFP Low in CT-A
GFP Low in CT-B
GFP-A
RFP-A

Supplementary Figure 12. Flow cytometry of GFP negative cells in Cas9 expressing cell lines

Comparing cell population with GFP low or negative in CT-A and CT-B directly provides Cas9 activity (see Methods). Associated data sets are “Extended Data Table 15 Cas9 activity.xlsx”

a

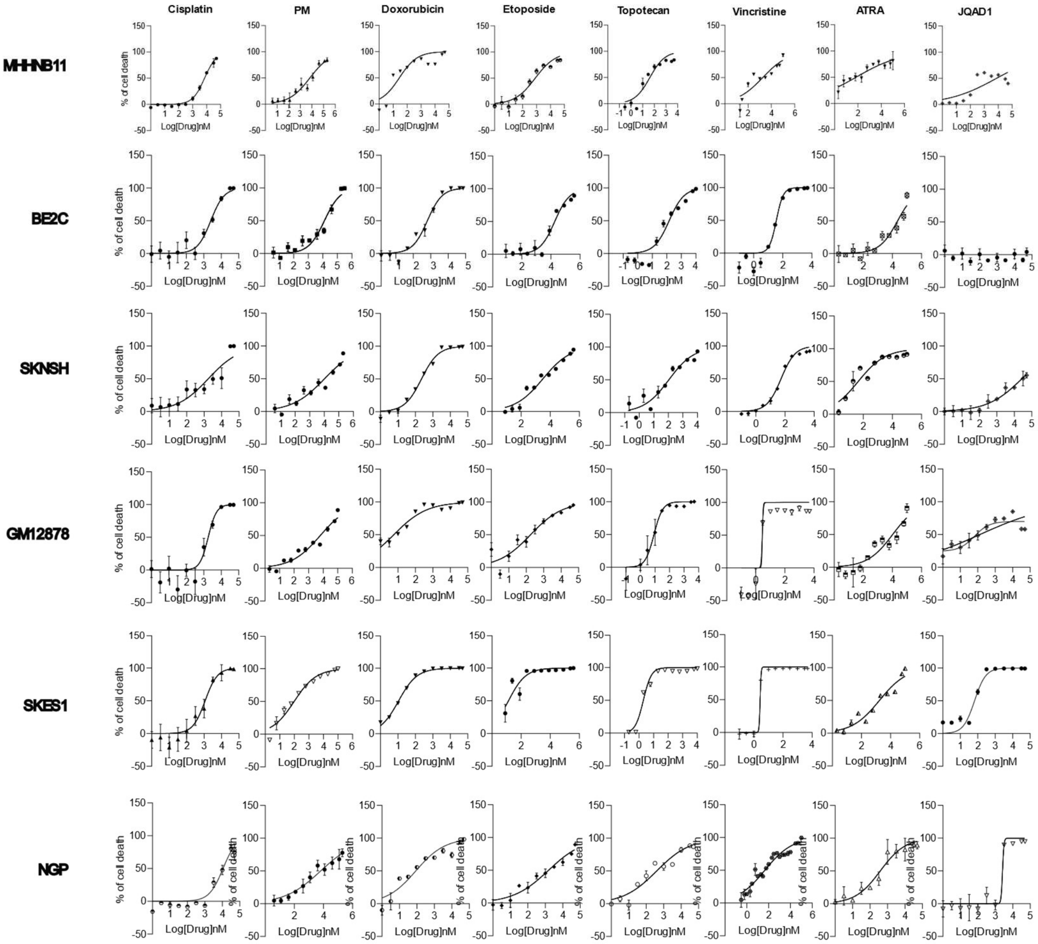
Cisplatin
PM
Doxorubicin
Etoposide
Topotecan
Vincristine
ATRA
JQAD1
MHHNB11
BE2C
SKNSH
GM12878
SKES1
NGP
% of cell death
Log[Drug]nM

b

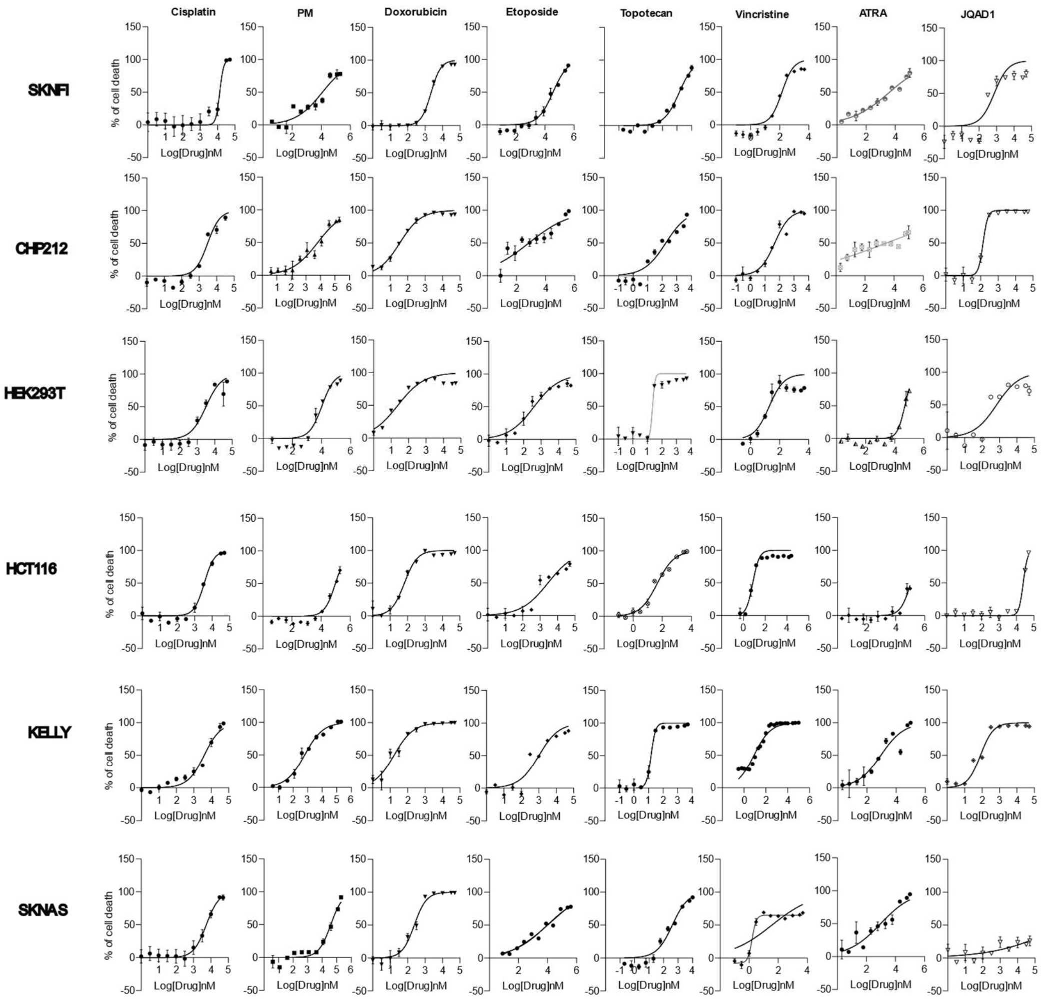
Cisplatin
PM
Doxorubicin
Etoposide
Topotecan
Vincristine
ATRA
JQAD1
SKNFI
CHP212
HEK293T
HCT116
KELLY
SKNAS
% of cell death
Log[Drug]nM

c

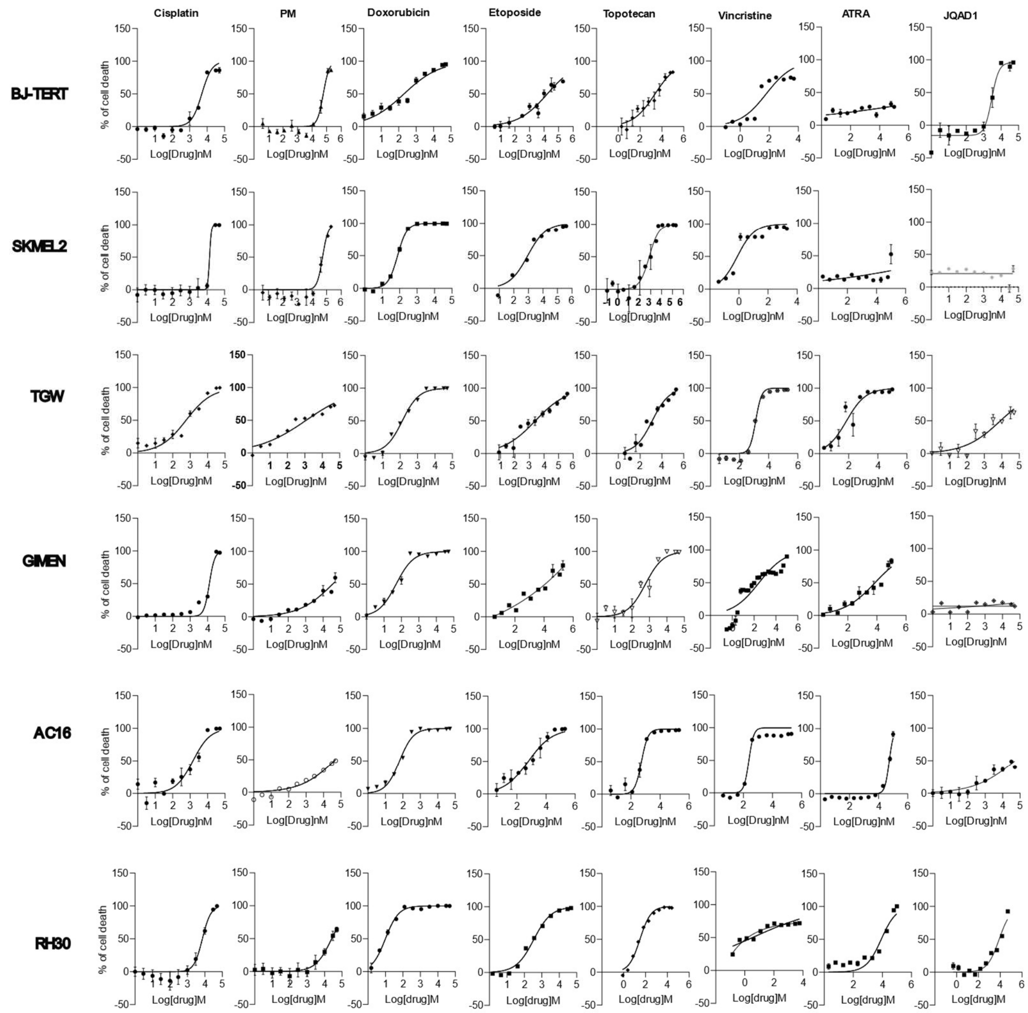

Supplementary Figure 13. Dose response of 8 drugs in 18 Cas9 expressing cell lines

(a-c) Dose dependent curves of 8 drugs and 18 Cas9 expressing cell lines to determine IC_{20}-IC_{30} for CRISPR screen.

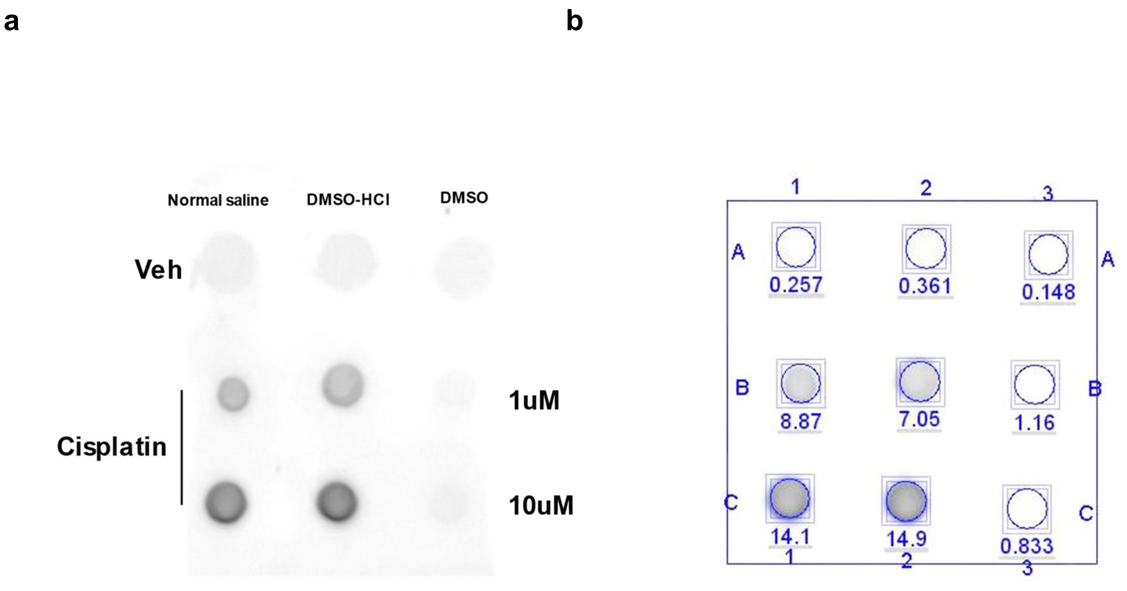

Supplementary Figure 14. Cisplatin-DNA adduct

(a) Dot blot of cisplatin-DNA adduct. (b) Measured intensity of each dot. DMSO-HCl overcomes instability of cisplatin in normal saline and activity loss of cisplatin in DMSO alone.

a

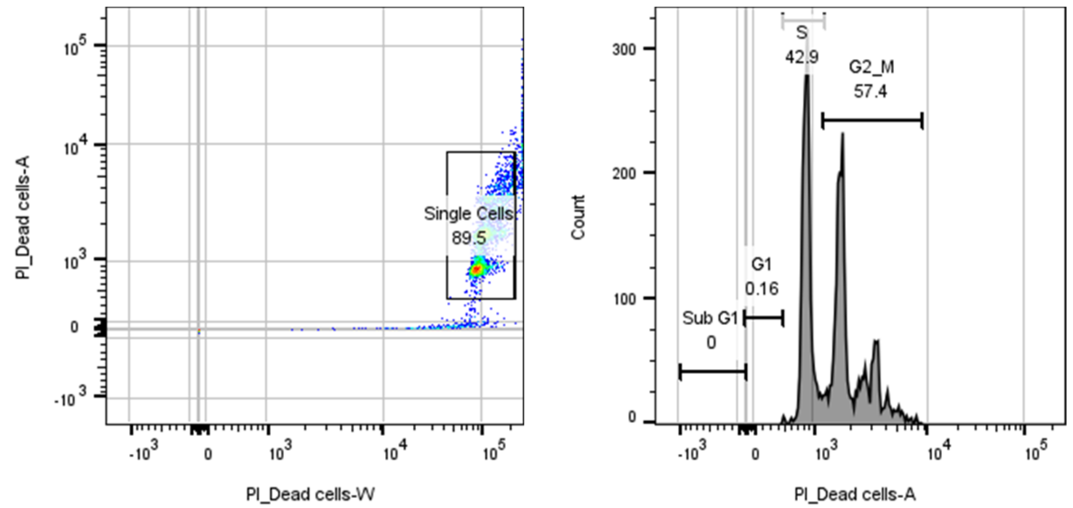
PI_Dead cells-A
PI_Dead cells-W
Single Cells
89.5
Count
Sub G1
0
G1
0.16
S
42.9
G2_M
57.4
PI_Dead cells-A

b

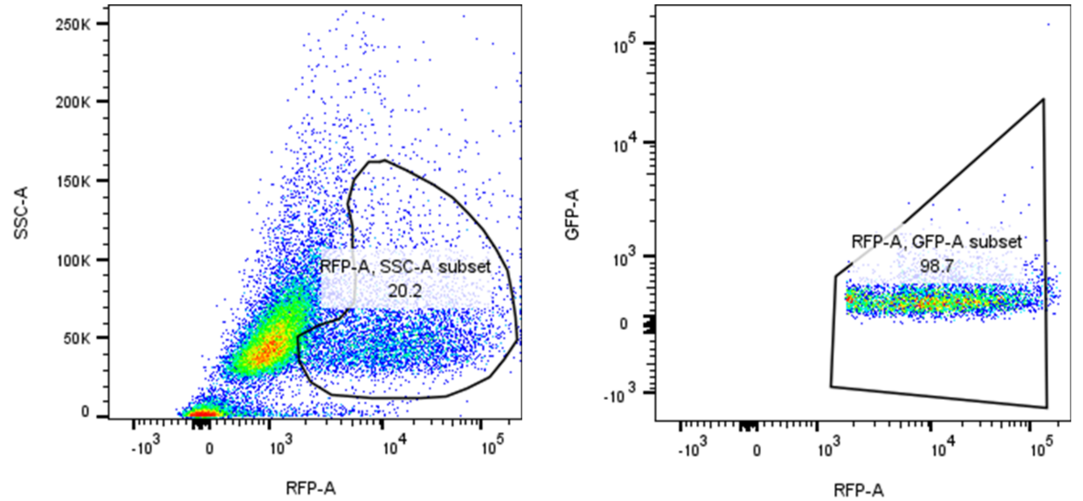
SSC-A
RFP-A
RFP-A, SSC-A subset
20.2
GFP-A
RFP-A
RFP-A, GFP-A subset
98.7

c

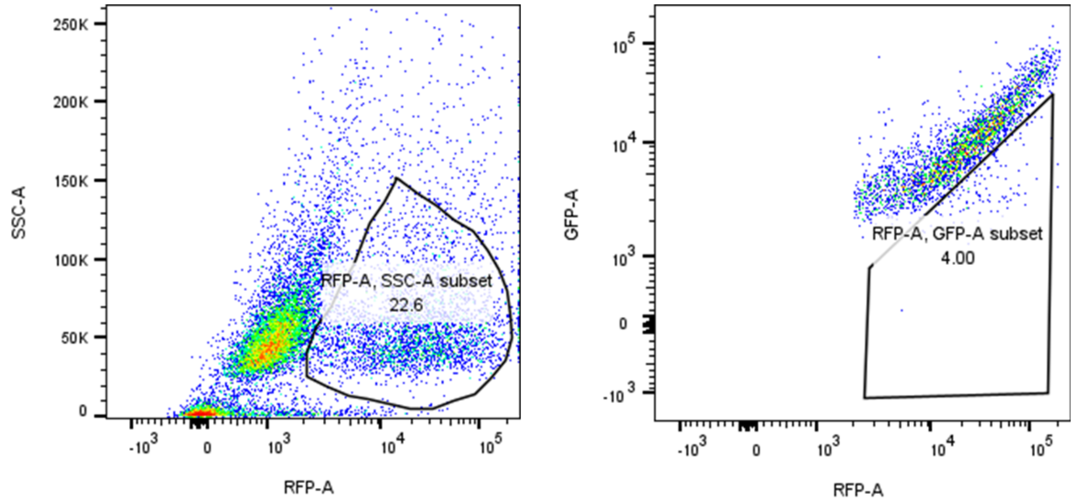
SSC-A
RFP-A
RFP-A, SSC-A subset
22.6
GFP-A
RFP-A
RFP-A, GFP-A subset
4.00

Supplementary Figure 15. FACS strategies

(a) FACS strategy for figure 6e and supplementary figure 7. Single cells were isolated by gating PI_dead cells-W vs. PI_dead cells-A (left). % of cell cycle phase was measured in PI_dead-A vs. count (right).
(b, c) FACS strategy for supplementary figure 9. RFP positive cells were isolated by gating RFP-A vs. SSC-A (left). GFP-low area was measured in RFP-A vs. GFP-A (right). (b) and (c) are CT-Active (CT-A, see Methods) and CT-background (CT-B, see Methods), respectively.
