# Supplementary material for: A CRISPR-drug perturbational map for identifying compounds to combine with commonly used chemotherapeutics
Source: Nat Commun. 2023 Nov 13;14:7332. doi: 10.1038/s41467-023-43134-0 (PMC10643606; doi:10.1038/s41467-023-43134-0)
Supplement: Supplementary file 17 — Reporting Summary [file 41467_2023_43134_MOESM17_ESM.pdf]

nature portfolio

Corresponding author(s): Paul Geeleher
Last updated by author(s): Oct 9, 2023

# Reporting Summary

Nature Portfolio wishes to improve the reproducibility of the work that we publish. This form provides structure for consistency and transparency in reporting. For further information on Nature Portfolio policies, see our Editorial Policies and the Editorial Policy Checklist.

## Statistics

For all statistical analyses, confirm that the following items are present in the figure legend, table legend, main text, or Methods section.

| n/a | Confirmed | |
|---|---|---|
| ☐ | ☒ | The exact sample size ($n$) for each experimental group/condition, given as a discrete number and unit of measurement |
| ☐ | ☒ | A statement on whether measurements were taken from distinct samples or whether the same sample was measured repeatedly |
| ☐ | ☒ | The statistical test(s) used AND whether they are one- or two-sided<br>*Only common tests should be described solely by name; describe more complex techniques in the Methods section.* |
| ☐ | ☒ | A description of all covariates tested |
| ☐ | ☒ | A description of any assumptions or corrections, such as tests of normality and adjustment for multiple comparisons |
| ☐ | ☒ | A full description of the statistical parameters including central tendency (e.g. means) or other basic estimates (e.g. regression coefficient) AND variation (e.g. standard deviation) or associated estimates of uncertainty (e.g. confidence intervals) |
| ☐ | ☒ | For null hypothesis testing, the test statistic (e.g. $F$, $t$, $r$) with confidence intervals, effect sizes, degrees of freedom and $P$ value noted<br>*Give P values as exact values whenever suitable.* |
| ☐ | ☒ | For Bayesian analysis, information on the choice of priors and Markov chain Monte Carlo settings |
| ☒ | ☐ | For hierarchical and complex designs, identification of the appropriate level for tests and full reporting of outcomes |
| ☐ | ☒ | Estimates of effect sizes (e.g. Cohen's $d$, Pearson's $r$), indicating how they were calculated |

*Our web collection on statistics for biologists contains articles on many of the points above.*

## Software and code

Policy information about availability of computer code

| | |
|---|---|
| Data collection | The code for this manuscript is available at https://osf.io/d9xgn/ (code used for analysis of data in the manuscript) and at https://github.com/wcwr/casava (code used to create the web app to explore the results). Both are accessible publicly/anonymously. |
| Data analysis | GraphPad Prism 9.1, R 4.2.1, OpenComet tool 1.3.1, BD DIVA 8.0, CellProfiler 4.2.4, Incucyte SX5 2022B, FlowJo 10.9.0, Mageck-Vispr/0.5.7, calc_auc_v1.1.py (https://github.com/mhegde/), count_spacers.py |

For manuscripts utilizing custom algorithms or software that are central to the research but not yet described in published literature, software must be made available to editors and reviewers. We strongly encourage code deposition in a community repository (e.g. GitHub). See the Nature Portfolio guidelines for submitting code & software for further information.

## Data

Policy information about availability of data

All manuscripts must include a data availability statement. This statement should provide the following information, where applicable:

- Accession codes, unique identifiers, or web links for publicly available datasets
- A description of any restrictions on data availability
- For clinical datasets or third party data, please ensure that the statement adheres to our policy

The raw sequencing data was generated for this study and have been deposited in GEO (GSE223991: https://www.ncbi.nlm.nih.gov/geo/query/acc.cgi?acc=GSE223991). All remaining data can be found in the Article, Supplementary, and Source Data files. Source data are provided with this paper (Source data file.xlsx).

The code for this manuscript is available at https://osf.io/d9xgn/ (code used for analysis of data in the manuscript) and at https://github.com/wcwr/casava (code used to create the web app to explore the results). Both are accessible publicly/anonymously.

Data and code availability is stated in the manuscript.

## Human research participants

Policy information about studies involving human research participants and Sex and Gender in Research.

| | |
|---|---|
| Reporting on sex and gender | N/A |
| Population characteristics | N/A |
| Recruitment | N/A |
| Ethics oversight | N/A |

Note that full information on the approval of the study protocol must also be provided in the manuscript.

# Field-specific reporting

Please select the one below that is the best fit for your research. If you are not sure, read the appropriate sections before making your selection.

[x] Life sciences  [ ] Behavioural & social sciences  [ ] Ecological, evolutionary & environmental sciences

For a reference copy of the document with all sections, see nature.com/documents/nr-reporting-summary-flat.pdf

# Life sciences study design

All studies must disclose on these points even when the disclosure is negative.

**Sample size**

The sample size was not calculated.
At least triplicates allowed to perform statistical analysis.

CRISPR-drug screen: one vehicle-treated & 8 single drug-treated in 18 cell lines (9 samples per cell line): No replicas
CRISPR-drug screen data analysis: 1) analyzed all 18 cell lines per drug, 2) analyzed by grouping 10 neuroblastoma vs. 8 controls
Cas9 activity test: two infected (CTA or CTB) cell lines in 18 cell lines: No replicas
shRNA efficiency test: one or two cell lines per shRNA: Knockdown efficiency of three shRNAs (4 genes = 12) was confirmed by western blot.
Knockdown test: one shRNA per one cell line (10-11), and four different shRNAs were confirmed.
Combocat: 3 replicas per dose in 18 cell lines
Figure 6a, b: 3 replicas
Figure 6c: two independent immunofluorescence, analyzed 3 images per treatment
Figure 6d: two independent comet assays, analyzed 5 images per treatment
Figure 6e: one experiment, two sets per treatment
Figure 6f: two independent tracking, analyzed 16-25 images per treatment
Figure 6g, h: one experiment, 4-5 mice per treatment

**Data exclusions**

None

**Replication**

CRISPR-drug screen: one vehicle-treated & 8 single drug-treated in 18 cell lines (9 samples per cell line)
Cas9 activity test: two infected cell lines in 18 cell lines, reproducible
shRNA efficiency test: one or two cell lines per shRNA (three shRNA/gene (total 4 genes)), reproducible
Knockdown test: one shRNA (best efficient one after shRNA efficiency test) per one cell line (x 10 - 11), reproducible
Combocat: single screening using 3 replicas per dose, per drug in 18 cell lines
Figure 6a.3 replicas, reproducible, except detecting N-terminal fragment
Figure 6b. 3 replicas, reproducible
Figure 6c: two independent immunofluorescence staining, analyzed 3 images per treatment, reproducible
Figure 6d: two independent comet assays, analyzed 5 images per treatment, reproducible
Figure 6e: one experiment, two sets per treatment
Figure 6f: two independent tracking, analyzed 25 images per treatment, reproducible
Figure 6g, h: one experiment, 4-5 mice per treatment

**Randomization**

Sample size was not calculated before experiments
CRISPR-drug screen: Individual 18 Cas9-expressing cells were pooled, not clonal.
To analyzed, a) CRISPR-drug screen data analysis: 1) analyzed all 18 cell lines per drug, 2) analyzed by grouping 10 neuroblastoma vs. 8

| | |
|---|---|
| | controls, b) all samples were grouped by vehicle control (2-3 replicas) vs. drug-treated samples (2-3 replicas)<br>Figure 6g, h: 4-5 mice per treatment were randomized, but all 5-week old female mice. |
| Blinding | The investigators were blinded to group allocation. |

# Reporting for specific materials, systems and methods

We require information from authors about some types of materials, experimental systems and methods used in many studies. Here, indicate whether each material, system or method listed is relevant to your study. If you are not sure if a list item applies to your research, read the appropriate section before selecting a response.

## Materials & experimental systems

| n/a | Involved in the study |
|---|---|
| ☐ | ☒ Antibodies |
| ☐ | ☒ Eukaryotic cell lines |
| ☒ | ☐ Palaeontology and archaeology |
| ☐ | ☒ Animals and other organisms |
| ☒ | ☐ Clinical data |
| ☒ | ☐ Dual use research of concern |

## Methods

| n/a | Involved in the study |
|---|---|
| ☒ | ☐ ChIP-seq |
| ☐ | ☒ Flow cytometry |
| ☒ | ☐ MRI-based neuroimaging |

## Antibodies

Antibodies used

Anti-Actin Sigma A2228
Anti-cisplatin-DNA Millipore MABE416
Anti-PRKDC Cell signal technology 38168S
Anti-phospho PRKDC (S2056) Cell signal technology 68716S
Anti-HDAC2 Santacruz Biotech sc-9959
Anti-MET Cell signal technology 8198S
Anti-KEAP1 Cell signal technology 8047S
Phospho-Histone H2A.X (S139) Cell signal technology 80312S

Secondary Anti-mouse, IRDye680 Licor 926-68070
Anti-rabbit, IRDye800 Licor 926-32211
Anti-rat, IRDye680 Licor 926-68076
Anti-rat, HRP Jackson Immuno 112-035-143
Anti-mouse, Alexa488 Invitrogen A31620
Anti-rabbit, Alexa594 Invitrogen A31632

Validation

Anti-Actin for Western blot: https://www.sigmaaldrich.com/US/en/product/sigma/a5316?gclid=CjwKCAjwyNSoBhA9EiwA5aYlb-GB5WlgEwI_uS8H_KUMd0vbO206-phjiqGu7KXusr6SIrpk13Ze8RoCkzgQAvD_BwE

Anti-cisplatin-DNA for dot blot: https://www.emdmillipore.com/US/en/product/Anti-Cisplatin-DNA-Adducts-Antibody-clone-ICR4,MM_NF-MABE416

Anti-PRKDC Cell for Western blot: https://www.cellsignal.com/products/primary-antibodies/dna-pkcs-e6u3a-rabbit-mab/38168?_requestid=4613694

Anti-phospho PRKDC (S2056) for Western blot: https://www.cellsignal.com/products/primary-antibodies/phospho-dna-pkcs-ser2056-e9j4g-rabbit-mab/68716

Anti-HDAC2 for Western blot: https://www.scbt.com/p/hdac2-antibody-c-8?gclid=CjwKCAjwyNSoBhA9EiwA5aYlbzA93srQnwH3RMaE_Q5Dgv0GEFqo_mDLLTz5KlgmobjPjz0fHRTE9RoCa4wQAvD_BwE

Anti-MET Cell signal technology 8198S: https://www.cellsignal.com/products/primary-antibodies/met-d1c2-xp-rabbit-mab/8198

Anti-KEAP1 Cell signal technology 8047S: https://www.cellsignal.com/products/primary-antibodies/keap1-d6b12-rabbit-mab/8047

Phospho-Histone H2A.X (S139) for immunofluorescence: https://www.cellsignal.com/products/primary-antibodies/phospho-histone-h2a-x-ser139-d7t2v-mouse-mab/80312

## Eukaryotic cell lines

Policy information about cell lines and Sex and Gender in Research

| | |
|---|---|
| Cell line source(s) | Neuroblastoma cell lines<br>MHHNB11 DSMZ ACC157<br>BE2C Easton@St.Jude In-house<br>NGP DSMZ ACC676<br>KELLY Sigma #92110411<br>CHP212 Shelat@St.Jude In-house<br>GIMEN DSMZ ACC654<br>SKNAS ATCC CRL-2137<br>TGW JCRB JCRB0618<br>SKNFI ATCC CRL-2142<br>SKNSH Sigma #86012802<br><br>Cancer cell lines<br>SKES1 Dyer@St.jude In-house<br>SKMEL2 Dyer@St.jude In-house<br>RH30 ATCC CRL-2061<br>HCT116 NCI<br><br>Non-cancer cell lines<br>HEK293T Chen@St.jude In-house<br>AC16 Millipore SCC109<br>BJ-TERT ATCC CRL-4001<br>GM12878 Easton@St.Jude In-house |
| Authentication | Neuroblastoma cell lines<br>MHHNB11 DSMZ ACC157: authenticated (STR)<br>BE2C Easton@St.Jude In-house: Not tested authentication<br>NGP DSMZ ACC676: authenticated (STR)<br>KELLY Sigma #92110411: authenticated (STR)<br>CHP212 Shelat@St.Jude In-house: Not tested authentication<br>GIMEN DSMZ ACC654: authenticated (STR)<br>SKNAS ATCC CRL-2137: authenticated (STR)<br>TGW JCRB JCRB0618: authenticated (STR)<br>SKNFI ATCC CRL-2142: authenticated (STR)<br>SKNSH Sigma #86012802: authenticated (STR)<br><br>Cancer cell lines<br>SKES1 Dyer@St.jude In-house: authenticated (STR)<br>SKMEL2 Dyer@St.jude In-house: authenticated (STR)<br>RH30 ATCC CRL-2061: authenticated (STR)<br>HCT116 NCI : authenticated (STR)<br><br>Non-cancer cell lines<br>HEK293T Chen@St.jude In-house: Not tested authentication<br>AC16 Millipore SCC109: authenticated (STR)<br>BJ-TERT ATCC CRL-4001: authenticated (STR)<br>GM12878 Easton@St.Jude In-house: authenticated (STR) |
| Mycoplasma contamination | All cell lines tested negative for mycoplasma contamination. |
| Commonly misidentified lines<br>(See ICLAC register) | No commonly misidentified cell lines were used in the study. |

## Animals and other research organisms

Policy information about studies involving animals; ARRIVE guidelines recommended for reporting animal research, and Sex and Gender in Research

| | |
|---|---|
| Laboratory animals | 5-week old female NSG mice (NOD.Cg-Prkdc scid Il2rg tm1Wjl /SzJ) were housed 12/12h light and dark, ambient temperature and humidity, pathogen-free conditions with food and water provided ad libitum. |
| Wild animals | No wild animals were used. |

| | |
|---|---|
| Reporting on sex | Female |
| Field-collected samples | No field collected samples were used. |
| Ethics oversight | All murine experiments were done in accordance with a protocol approved by the Institutional Animal Care and Use Committee of St. Jude Children's Research Hospital. |

Note that full information on the approval of the study protocol must also be provided in the manuscript.

# Flow Cytometry

## Plots

Confirm that:

- [x] The axis labels state the marker and fluorochrome used (e.g. CD4-FITC).
- [ ] The axis scales are clearly visible. Include numbers along axes only for bottom left plot of group (a 'group' is an analysis of identical markers).
- [ ] All plots are contour plots with outliers or pseudocolor plots.
- [x] A numerical value for number of cells or percentage (with statistics) is provided.

## Methodology

| | |
|---|---|
| Sample preparation | 1. 18 Cas9-expressing cells were infected by CT-active [CT-A] or CT-background [CT-B] premade lentiviruses and maintained the infected cells for 10 days to avoid 100% confluence. After 10 days, the cells were harvested to measure the changes in GFP levels by flow cytometry.<br><br>2. Two neuroblastoma cell lines, BE2C and GIMEN were incubated with serum free medium for 48 hrs to arrest G0/G1. 48 hr after starvation, the cells were released by adding fresh complete growth medium. BE2C and GIMEN were treated with 100ng/mL of nocodazole (NOC) for 18 hrs to arrest G2/M phase. 18 hrs after treatment, the cells were released by washing out NOC three times and replaced with fresh complete growth medium. 24 hrs after release of each synchronization, the cells were treated with vehicle (DMSO) or drugs for 72 hrs. The treated cells were harvested at 1000RPM for 10 min, fixed with 70% ethanol at -20C for 2 hrs, then washed with 1x PBS, and staining with propidium iodide (50ug/mL) for FACS. |
| Instrument | BD Flow cytometer EQ |
| Software | BD DIVA 8.0, FlowJo 10.9.0 |
| Cell population abundance | 1. Isolated GFP positive cell population<br><br>2. Separated cell cycle phases based on propidium iodide (PI) |
| Gating strategy | 1. RFP positive / SSC, followed by RFP positive / GFP positive<br><br>2. PI-W/PI-A (=single cell), followed by histogram of PI-A |

- [x] Tick this box to confirm that a figure exemplifying the gating strategy is provided in the Supplementary Information.
