# Supplementary material for: A CRISPR-drug perturbational map for identifying compounds to combine with commonly used chemotherapeutics
Source: Nat Commun. 2023 Nov 13;14:7332. doi: 10.1038/s41467-023-43134-0 (PMC10643606; doi:10.1038/s41467-023-43134-0)
Supplement: Supplementary file 18 — Description of Additional Supplementary Files [file 41467_2023_43134_MOESM18_ESM.pdf]

Title: Supplementary data 1.

Description: This table contains sgRNA library (SKOOL) information.

Description: sgRNA sequence, gene, exon number, and others. These information is generated by using CRISPick (1, 2)
(1) Nat Biotechnol 34, 184–191 (2016). https://doi.org/10.1038/nbt.3437
(2) Nat Comms 9, 5416 (2018). https://doi.org/10.1038/s41467-018-07901-8"

Non targeting sgRNA: Non targeting sgRNA sequence (Note: Tab "sgRNA" contains the same information). These are gRNA sequence that does not target known human, mouse and rat genes. They serve as a negative control.
Essentiality: List of DepMap essential genes in our targeted 655 genes per each cell line.The DepMap essential genes are highlighted based on the score of DepMap Chronos < (=) -1 (below -1). The numbers are sorted from the smallest (implicating the most essential genes) in each cell line.
Biomarkers: List of known genetic or molecular biomarkers for neuroblastoma in our targeted 655 genes. 8 biomarkers in our targeted CRISPR screen (8 in 655 genes). All biomarkers for neuroblastoma are described at [DOI: 10.1007/s11864-021-00898-1].

Title: Supplementary data 2.

Description: This table contains detailed cell line information and drug dose in CRISPR screen (List of 18 cell lines screened in our study).

CELL_LINE: Name of cell line, DepMap_ID: DepMap ID if available, CaseControl & LINEAGE: Type of cancers, Category: ADR or MES, ADR: Adrenergic MES: Mesenchymal, ADR_MES ratio: Ratio of ADR and MES gene expression level, Expression_Cluster, Dependency_Cluster.
DepMap Copy Number Public 22Q4: Relative copy number extracted from DepMap.
Biomarkers; blue highlighted, MYCN_CN: Copy number of MYCN.
CN Gain/Amp (> 1.25): pink highlighted MYC_CN
CN Loss/ Del (< 0.75): green highlighted TP53_CN
DepMap Expression (Log2(TPM+1)):Gene expression level extracted from DepMap
Relatively high expression (exp), Mutation (mut)
Hotspot mutations: yellow highlighted (TP53_mut: point mutation or amino acid deletion, P53_Function LOF: Loss of function)
Hotspot mutations: yellow highlighted (ALK_mut: point mutation, fs: frame shift, ALK_Function GOF: Gain of function or LOF)
Drugs in CRISPR screen [µM, (nM)] Cisplatin, PM (Phosphoramide mustard), Doxorubicin, Etoposide, Topotecan, Vincristine, ATRA(all trans retinoic acid), JQAD1.

Title: Supplementary data 3.

Description: This table contains CRISPR screen results of comparison in between SKOOL and Brunello using MAGeCK analysis (MAGeCK (Model-based Analysis of Genome-wide CRISPR/Cas9 Knockout).

Gene: List of 655 genes, -Log10(RRA|neg): Negative log robust rank aggregation in negative selection CRISPR screen of CHP-134 & SKOOL or Brunello, P value|neg: P value in negative selection, -Log10(RRA|pos): Negative log robust rank aggregation in positive selection CRISPR

screen of CHP-134 & SKOOL or Brunello, P value|pos: P value in positive selection. LFC: log2 fold change, CHP-134: Neuroblastoma cell line, CX-5461: anchor drug, TOP2 inhibitor, SKOOL: SJ Knockout non-lethal sgRNA library, Brunello: Genome wide CRISPR knockout library.

Title: Supplementary data 4.

Description: This table contains CRISPR screen results of all 18 cell lines (18 tabs) and 8 drugs analyzed using MAGeCK (MAGeCK (Model-based Analysis of Genome-wide CRISPR/Cas9 Knockout).

Cell lines: MHHNB11, BE2C, AC16, BJ-TERT, CHP212, GIMEN, HCT116, HEK293T, KELLY, GM12878(LCL), NGP, RH30, SKES1,SKMEL2, SKNAS, SKNFI, SKNSH, TGW.
Drugs: Cisplatin, PM (Phosphoramide mustard), Doxorubicin, Etoposide, Topotecan, Vincristine, ATRA(all trans retinoic acid), JQAD1.

LFC: log2 fold change, -Log(P value|neg): Negative log P value in negative selection, -Log(P value|pos): Negative log P value in positive selection, -Log(RRA|neg): Negative log robust rank aggregation in negative selection CRISPR screen, -Log(RRA|pos): Negative log robust rank aggregation in positive selection CRISPR screen.

Title: Supplementary data 5.

Description: This table contains model-based gene depletion and enrichment (MAGeCK) for 8 drugs in all 18 cell lines.

Negative logFC indicates gene depletion (sensitizing, on the left of plots) & Positive logFC indicates gene enrichment (resistance, on the right of plots).

Drugs: CDDP (Cisplatin), pm (phosphoramide mustard), Doxorubicin, Etoposide, Topotecan, Vincristine, ATRA (all trans retinoic acid), JQAD1.

Rank: Gene ranking based on logFC, Gene: gene name, logFC: log2 fold change, Senstizing_P: P value in sensitizing, Resistance_P: P value in resistance, Sensitizing_FDR: false discovery rate in sensitizing, Resistance_FDR: false discovery rate in resistance. Ref: Previous reports suggesting potential combination targets with anchor drug.

Title: Supplementary data 6.

Description: This table contains lists of candidate genes of sensitization referenced against previous findings in the literature and clinical trials.

Each numbers Negative Log robust rank aggregation in positive or negative selection (highlighted blue (R; resistance) or yellow (S; sensitizing)). Above 1.8 is statistically significant (P < 0.05), In between 1.4-1.8 is borderline (P = ~0.05), In between 1.3-1.4 is marginal (0.05 < P < 0.06), Below 1.3 is statistically insignificant.

Title: Supplementary data 7.

Description: This data contain gene set analysis data (analyzed by the joint model described in Methods) of 1) mSigDb Hallmark gene sets (first 9 tabs), 2) gene family (next 9 tabs), and 3) genes targeted by the same drug (last 9 tabs).

Hallmark: Hallmark gene sets, gene: Name of genes, Drug: Name of drugs.
geneFamPs: P values in gene set, pAdj: adjusted P value, medianDiff: Median difference between non targeting controls and genes in the gene set. A negative number implies knockout of genes in that gene set causes drug resistance. genesInGeneset: List of screened genes belonging to this gene set.

Title: Supplementary data 8.

Description: This table contains the complete data supporting Figure 3L indicating sensitizers to DNA damaging agents using a joint model.

Gene: List of genes by logFC ranking, including non-targeting, logFC: log2 fold change, Senstizing_P: P value in negative selection, Sensitizing_FDR: false discovery rate in negative selection.

Title: Supplementary data 9.

Description: This table contains the results of a model estimating differential sensitization in the neuroblastoma group vs the outgroup cell lines.

NB_effectSize Neuroblastoma effect size. A negative effect indicates sensitization in the neuroblastoma cell lines. NB_Senstizing_P: P value of neuroblastoma effect size for sensitizing candidates. NB_Resistance_P: P value of neuroblastoma effect size for resistance candidates. NB_Sensitizing_FDR: false discovery rate of neuroblastoma effect size for sensitizing candidates. NB_Resistance_FDR: false discovery rate of neuroblastoma effect size for resistance candidates. differential_effectSize: Effect size estimating the difference between the neuroblastoma group and the outgroup. differential_Senstizing_P: P value in comparing ourgroup effect size for sensitizing candidates. differential_Resistance_P: P value in comparing ourgroup effect size for resistance candidates. differential_Sensitizing_FDR: false discovery rate in comparing outgroup effect size for sensitizing candidates. differential_Resistance_FDR: false discovery rate in comparing outgroup effect size for resistance candidates.

Title: Supplementary data 10.

Description: This table contains the data comparing CRISPR screen (-Log(RRA|neg)) and shRNA knockdown effects with drug treatment.

PRKDC, HDAC2, KEAP1, MET: Target gene of knockdown. Dox: Doxorubicin, Pat: Parental cell line (non-knockdown of target genes). KD: Knockdown of target genes. P: P value, Significance: 95% significance based on unpaired t-test.

Title: Supplementary data 11.

Description: This table contain the data to compare CRISPR screen (-Log(RRA|neg)) and median ZIP (zero interaction potency) score from the drug combination screening.

Index: Series of drug test, d1_conc: Drug 1 concentration, d2_conc: Drug 2 concentration, Neg.log(RRA|neg): Negative log of robust rank aggregation in negative selection CRISPR screen, MED: Median of ZIP.

Title: Supplementary data 12.

Description: This table contains analysis of phosphor-H2AX in BE2C and GIMEN.

Input: image file names
Image info: identification of cell line (BE2C or GIMEN) and treatment

Input	Image info
be ct gr2.tif	BE2C vehicle control
be dox gr.tif	BE2C doxorubicin treated
be azd gr.tif	BE2C AZD7648 treated
be combo gr.tif	BE2C combo treated
gm ct gr2.tif	GIMEN vehicle contro
gm dox gr.tif	GIMEN doxorubicin treated
gm azd gr.tif	GIMEN AZD7648 treated
gm combo gr.tif	GIMEN combo treated

drug	Treatment
Veh	DMSO vehicle
Dox	Doxorubicin
AZD	AZD7648
Combo	Combo: Doxorubicin and AZD7648

Count_Nuc	Number of nuclei
SEM	standard error of mean
Count_gH2AX	Number of of phospho H2AX
Count_pPRKDC	Number of phospho PRKDC
MEAN Int H2AX	Mean intensity of phospho H2AX
MEAN Int Nuc	Mean intensity of nuclei
MEAN Int PK	Mean intensity of phospho PRKDC
MED Int H2AX	Median intensity of phospho H2AX
MED Int Nuc	Median intensity of nuclei
MED Int PK	Median intensity of phospho PRKDC

Title: Supplementary data 13.

Description: This table contains analysis of tumor volume in mice treated with doxorubicin and the PRKDC inhibitor AZD7648.

Mean: average tumor volume in each treatment, df: degree of freedom, P: P value, Adj P: Adjusted P value (unpaired t -test).

Title: Supplementary data 14.

Description: This table contains reagent information.

Title: Supplementary data 15.

Description: This table contains analysis of Cas9 activity in each cell line based on fluorescence change.

GFP Low in CT-A: % of cells without or low GFP in CT-A (GFP-targeted sgRNA), GFP Low in CT-B: % of cells without or low GFP in CT-B (non-targeting sgRNA), Cas9 Activity (%): Calculated Cas9 activity based on % of cells in CT-A and CT-B.
